# 2-Aminobenzaldehydes as Versatile Substrates for Rhodium-Catalyzed Alkyne Hydroacylation: Application to Dihydroquinolone Synthesis**

**DOI:** 10.1002/anie.201308127

**Published:** 2013-11-12

**Authors:** Matthias Castaing, Sacha L Wason, Beatriz Estepa, Joel F Hooper, Michael C Willis

**Affiliations:** Department of Chemistry, University of Oxford, Chemistry Research LaboratoryMansfield Road, Oxford, OX1 3TA (UK)

**Keywords:** aldehydes, amines, dihydroquinolones, enones, rhodium catalysis

Alkene and alkyne hydroacylation reactions are archetypal examples of simple addition processes that display excellent atom economy.^1^ Both reactions result in the formation of a new C–C bond and deliver synthetically useful carbonyl-containing products.^2^ In recent years, there has been considerable interest in converting these processes into synthetically useful transformations. Transition-metal-catalyzed variants represent the largest class of hydroacylation reactions, and amongst these, processes that involve some form of chelation control dominate. The need to employ a chelating substrate stems from the fact that the majority of the metal-catalyzed examples proceed through an inherently unstable acyl metal intermediate **1** (Scheme 1), which can lead to the formation of unwanted side products formed by decarbonylation. A limitation of the chelation-controlled strategy is that the coordinating group, which is present to stabilize the metal–acyl intermediate **2**, will also be present in the product. If this group is not needed in the final product, then it must be removed or converted into an alternative functional group.^3^ Despite this limitation, the advantages of this chelation-controlled process, such as mild reaction conditions, control of enantio- and regioselectivity,^4^, ^5^ and broad substrate scope, have resulted in widespread applications of this approach. One strategy to overcome the innate limitation of a chelation-controlled approach is to develop catalytic methods that function without the need for such coordinating groups; although there are notable examples of success with this approach,2c, 6 significant limitations with regard to substrate scope and enantio- and regioselectivity remain. An alternative strategy is to consider the need for a chelating unit as an opportunity, and to expand the range of effective coordinating groups, so that a large variety of useful functional groups can act as the crucial chelating motif. As synthetic chemistry is generally concerned with the preparation of functionalized molecules, an approach that is tolerant of, or indeed benefits from, as many useful functional groups as possible should find wide application. Herein, we demonstrate that simple and readily available 2-aminobenzaldehydes are excellent substrates for intermolecular Rh-catalyzed alkyne hydroacylation, and in doing so add to the motifs available for use in these valuable processes. Furthermore, the products of these reactions, amino-substituted enones, were directly converted into a series of useful dihydroquinolone heterocycles.

**Scheme 1:**
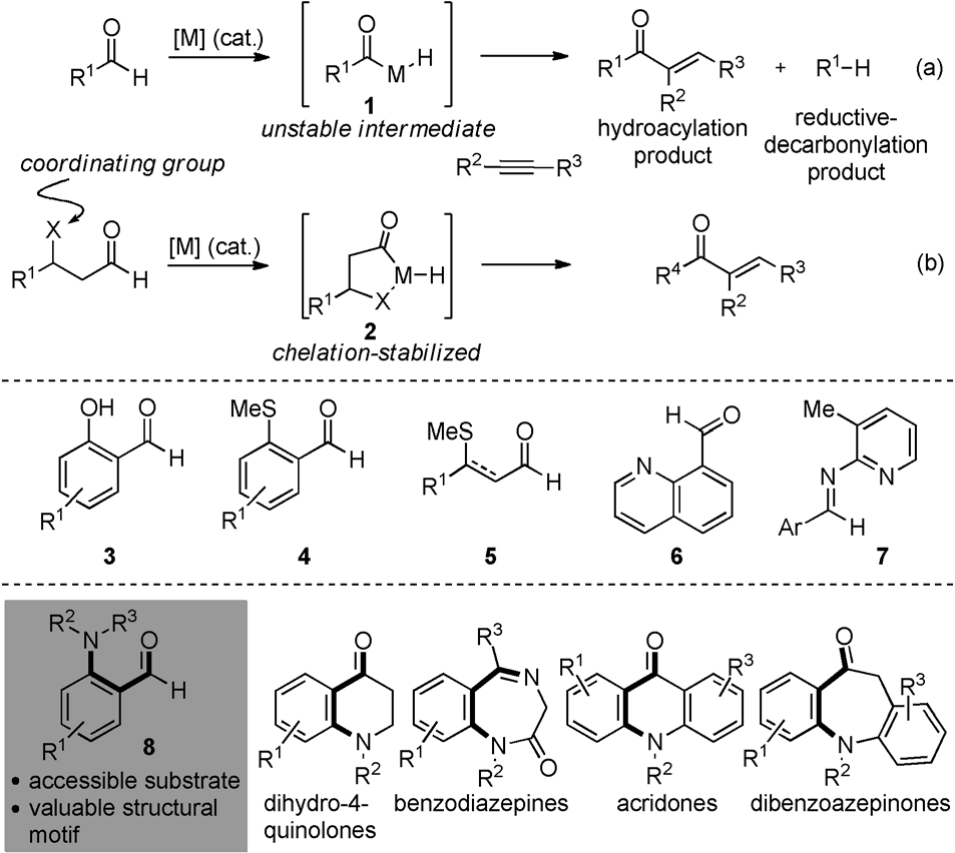
Chelation-free (a) and chelation-controlled intermolecular alkyne hydroacylation (b). The most common chelating aldehyde motifs are also shown (**3**–**7**), along with the 2-aminobenzaldehyde framework **8** and examples demonstrating the ubiquity of the 2-amino-carbonyl unit in biologically significant heterocycles.

The first intermolecular metal-catalyzed alkene hydroacylation, which employed an aldehyde with a coordinating C=C bond, was reported by Lochow and Miller.^7^ Since this initial report, the most popular substrates for chelation-controlled reactions feature heteroatom coordination, and systems that include oxygen (**3**),^8^ sulfur (**4**, **5**),^9^, ^10^ and, to a more limited extent, phosphorus^11^ substituents have all been reported. Although there are also some precedents for the use of nitrogen-based functional groups, examples are scarce and mostly either poor yielding or limited to very specific substrates. Suggs first reported the use of nitrogen chelation when he employed quinoline-8-carboxyaldehyde (**6**) as a substrate in the presence of a stoichiometric amount of Wilkinson’s complex.^12^ Picolyl imines **7** were first used as removable or catalytic chelating groups by Suggs;^13^ significant advances were then achieved by Jun et al. and other groups.^14^ However, these reactions require harsh reaction conditions (typically 130–170 °C) and high catalyst loadings. More recently, Stemmler and Bolm have shown that a single aniline derivative is an effective substrate for intermolecular alkene hydroacylation, but only strained and highly reactive norbornadiene was a suitable reaction partner.^15^ Bendorf et al. have shown that N-tethered aldehydes can be employed in intramolecular reactions.^16^, ^17^ Given the importance of nitrogen-containing molecules to a great number of applications, we were attracted to the idea of developing a simple, readily available family of aldehydes that feature N-chelation for intermolecular hydroacylation reactions.

The *ortho*-amino-carbonyl scaffold is a versatile synthetic unit in its own right,^18^ but this moiety is also embedded in a variety of important heterocycles, such as 4-quinolones,^19^ acridones,^20^ benzodiazepines,^21^ and dibenzoazepinones,^22^ which feature in a number of important medicinal agents and natural products. Given this versatility, we targeted the use of simple *ortho*-aminobenzaldehydes **8** in intermolecular hydroacylation reactions. Using 2-(pyrrolidin-1-yl)benzaldehyde **8 a** and 1-hexyne as the test substrates, we evaluated a number of Rh-based catalysts (Table 1). In agreement with our recent studies on sulfur-chelating aldehydes,^23^ electron-rich diphosphine ligands with a small bite angle were found to generate the most active catalysts; a complex that incorporates dcpm as the ligand achieved complete conversion of the aldehyde after 30 min in acetone at 55 °C (entries 1–5). In line with our previous reports, the addition of small quantities of MeCN allowed the catalyst loading to be reduced; the reactions still proceeded efficiently with only 2 mol % of the rhodium precursor (entries 7–9).^23^

**Table 1:** Catalyst screening for the hydroacylation of 1-hexyne with aldehyde 8 a[a] 
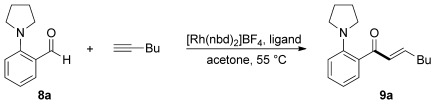

Entry	Ligand	[Rh] [mol %]	MeCN [mol %]	*t* [h]	Conv.[b] [%]
1	DPEphos	10	0	16	25
2	dppm	10	0	16	94
3	dppe	10	0	16	78
4	dppp	10	0	16	8
5	dcpm	10	0	0.5	100 (82)[c]
6	dcpm	5	0	2	95
7	dcpm	5	10	2	100
8	dcpm	2	0	2	11
9	dcpm	2	4	2	93

[a] Reaction conditions: **8 a** (1.0 equiv), 1-hexyne (1.5 equiv), [Rh(nbd)_2_]BF_4_, ligand, acetone (0.15 m), 55 °C.

[b] Determined by ^1^H NMR spectroscopy.

[c] Yield of isolated product. nbd=norbornadiene. 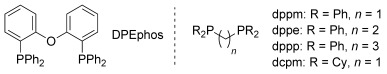

With suitable reaction conditions in hand, we next explored the variation of the 2-aminobenzaldehyde component in reactions with phenylacetylene (Scheme 2). The reaction was found to proceed well with a number of different tertiary amines at the 2 position (**9 b**–**e**). Benzaldehydes substituted with secondary or primary amines were also competent substrates for this transformation (**9 f**, **g**). With *N*-methyl-*N*-3,4-dimethoxybenzyl (DMB)-derived aldehydes, we were able to establish that both electron-donating (**9 h**, **i**) and electron-withdrawing (**9 k**, **l**) substituents could be positioned around the arene core. Only substitution at the C6 position was not tolerated (**9 j**). Pleasingly, it was possible to employ a heterocyclic aldehyde, as a thiophene-derived aldehyde delivered the corresponding enone **9 m** in excellent yield. In all cases, we only observed formation of the linear isomer. For pragmatic reasons, all of the examples presented in Scheme 2 were performed using 5 mol % of the catalyst. However, we were also able to significantly reduce this loading. For example, enone **9 e**, which features DMB and Me substituents on the nitrogen atom, was efficiently prepared using either 2 mol % (2 h, 95 % yield) or 1 mol % (4 h, 84 %) of catalyst. When the loading was further reduced to 0.5 mol %, a maximum yield of 45 % was obtained.

**Scheme 2:**
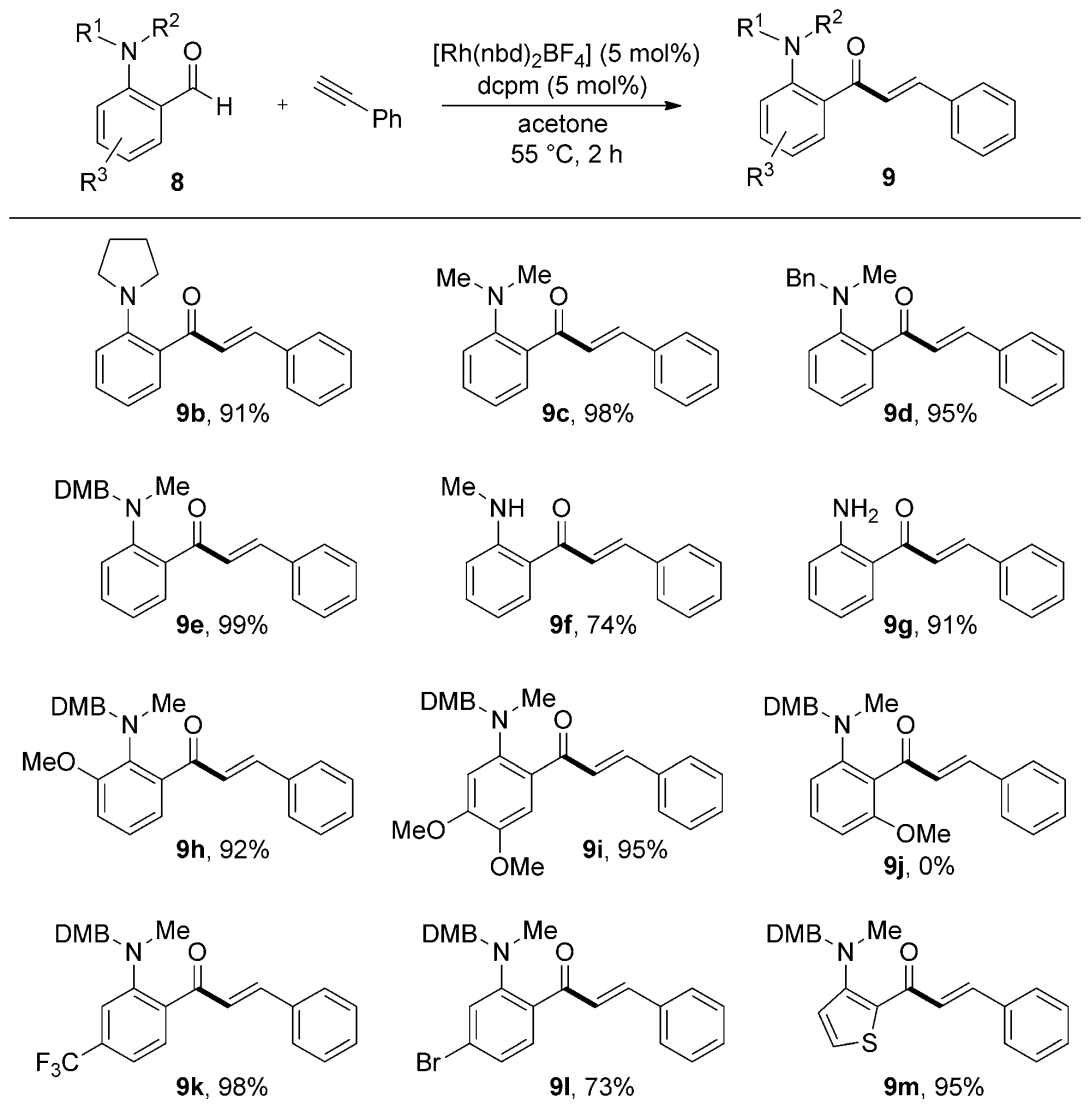
Variation of the 2-aminobenzaldehyde coupling partner in Rh-catalyzed hydroacylation reactions with phenylacetylene. Reaction conditions: **8** (1.0 equiv), phenylacetylene (1.5 equiv), [Rh(nbd)_2_]BF_4_ (5 mol %), dcpm (5 mol %), MeCN (10 mol %), acetone (0.15 m), 55 °C. Yields of isolated products are given. DMB=3,4-dimethoxybenzyl.

We next evaluated the variation of the alkyne component and initially selected *N*-DMB-*N*-Me-2-aminobenzaldehyde **8 b** as the standard coupling partner (Scheme 3). A number of alkyl acetylenes were efficiently transformed under the standard reaction conditions (**9 n**, **o**), including a sterically demanding derivative with a *tert*-butyl substituent (**9 p**). A variety of functional groups, such as chloro- or cyano-substituents (**9 q**, **r**) as well as a vinyl silane (**9 s**) and a ferrocenyl unit (**9 t**), were also smoothly incorporated into the corresponding products. A fluoro-substituted aryl acetylene could also be employed (**9 u**). It was also possible to use internal alkynes, with 1-phenyl-1-propyne delivering enone **9 v** essentially as a single isomer. We were pleased to discover that functionalized alkynes could be effectively combined with the parent, primary amine-derived, 2-aminobenzaldehyde (**8 c**); reaction with 1-octyne, cyclohexylacetylene, and 3-hexyne all furnished the expected enones (**9 w**–**y**) in good yields.

**Scheme 3:**
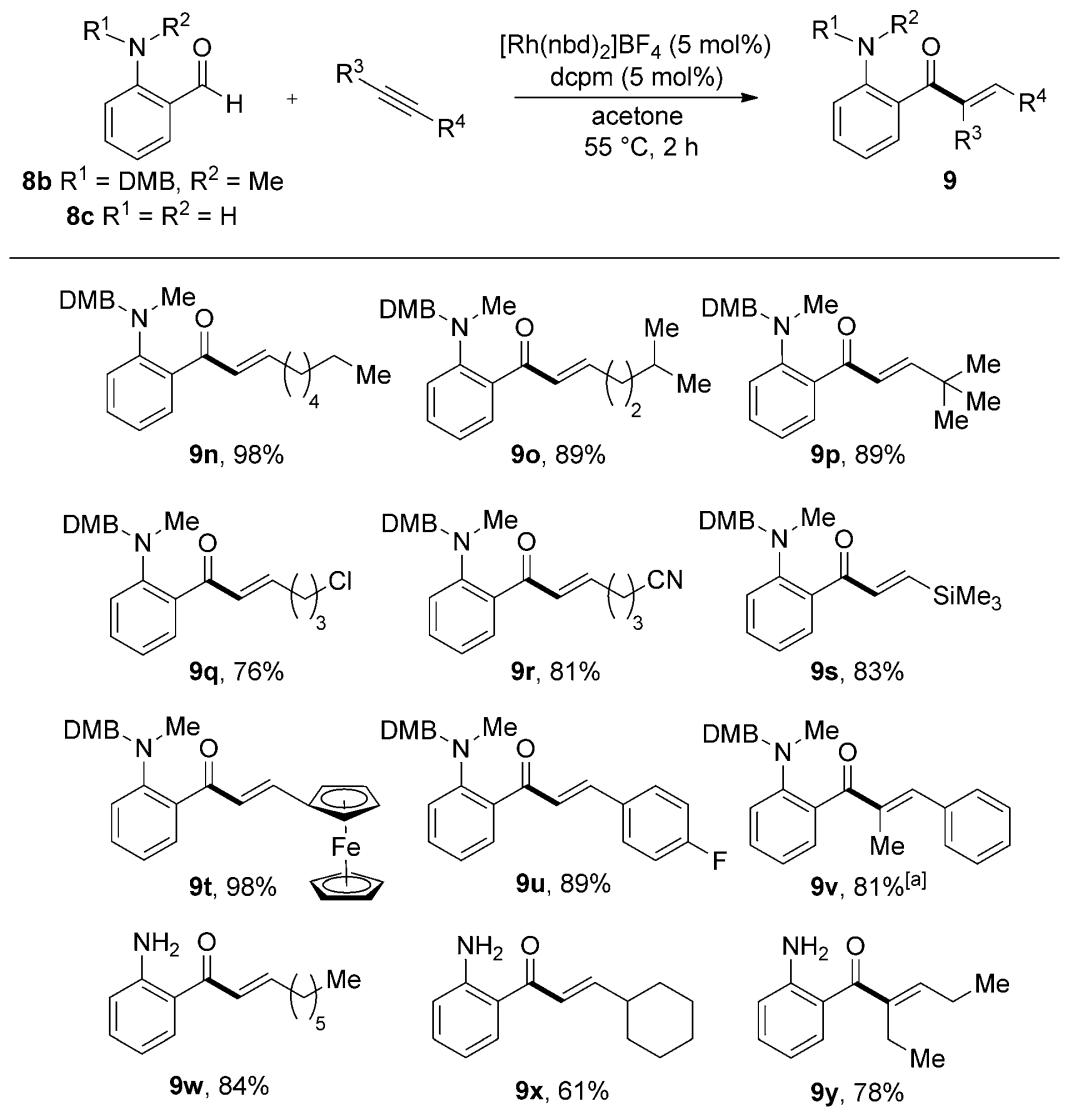
Variation of the alkyne coupling partner in Rh-catalyzed hydroacylation reactions with aminobenzaldehydes **8 b** and **8 c**. Reaction conditions: **8 b** or **8 c** (1.0 equiv), alkyne (1.5 equiv), [Rh(nbd)_2_]BF_4_ (5 mol %), dcpm (5 mol %), MeCN (10 mol %), acetone (0.15 m), 55 °C. Yields of isolated products are given. [a] Regioisomeric ratio >20:1, determined by ^1^H NMR spectroscopy.

Having established efficient conditions for the preparation of a broad range of 2-aminoaryl enones using hydroacylation chemistry, we explored the conversion of these functionalized building blocks into useful heterocyclic products. Dihydro-4-quinolones are a family of heterocycles that display a variety of biological properties, including antibacterial and antitumor activities.^24^ They have also been used as building blocks in the synthesis of natural products.^25^ For products that bear primary amines (**9 g**, **9** **w**–**y**), cyclization to the dihydroquinolones was achieved by treatment with antimony trichloride, following a procedure by Maiti and co-workers (Scheme 4).^26^, ^27^ Good yields were obtained with aryl- and alkyl-substituted enones (**10 a** and **10 b**, **c**, respectively). Cyclization of 2,3-disubstituted enone **9 y** provided the corresponding dihydroquinolone as a 3:1 mixture of diastereomers (**10 d**). Treatment of the DMB-protected hydroacylation products with triflic acid resulted in smooth removal of the DMB group and conversion into the secondary anilines, which were directly employed in the cyclization reactions. With the exception of the trimethylsilyl-substituted enone (**9 s**→**10 k**), the hydroacylation products could all be converted into the corresponding dihydroquinolones using antimony trichloride. Pleasingly, the thiophene-derived enone also cyclized efficiently to provide the interesting thienyl dihydropyridone **10 s**. All of these results demonstrate that a combined hydroacylation/cyclization approach allows the efficient synthesis of dihydroquinolones in two operations from commercially available or readily prepared starting materials with substituents possible at every position bar one.

**Scheme 4:**
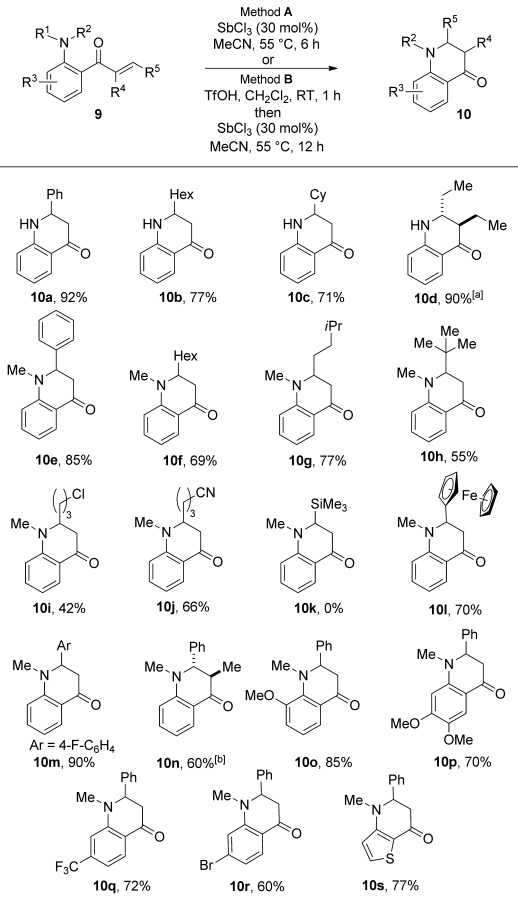
Conversion of amino-substituted enones **9** into 4-quinolones **10**. Method **A**: 2-amino-aryl enone (1.0 equiv), SbCl_3_ (0.3 equiv), MeCN (0.3 m), 55 °C. Method **B**: 2-amino-aryl enone (1.0 equiv), TfOH (3.0 equiv), CH_2_Cl_2_ (0.1 m), 25 °C; followed by SbCl_3_ (0.3 equiv), MeCN (0.3 m), 55 °C. [a] d.r.=3:1, determined by ^1^H NMR spectroscopy. [b] d.r.=4:1, determined by ^1^H NMR spectroscopy. Cy=cyclohexyl.

In summary, we have developed an efficient and versatile method for the hydroacylation of a wide range of alkynes with 2-aminobenzaldehyde derivatives, the products of which can be easily cyclized to give dihydroquinolones, an important class of heterocycles. The hydroacylation reactions proceeded in high yields with low catalyst loadings and short reaction times, and a commercially available catalyst was employed. The effective use of 2-aminobenzaldehydes provides another example of chelation-assisted strategies for hydroacylation reactions, which now encompass a broad range of substrates and lead to diverse and synthetically useful products.

## Supporting Information

As a service to our authors and readers, this journal provides supporting information supplied by the authors. Such materials are peer reviewed and may be re-organized for online delivery, but are not copy-edited or typeset. Technical support issues arising from supporting information (other than missing files) should be addressed to the authors.

miscellaneous_information
