# Supplementary material for: 2-Aminobenzaldehydes as Versatile Substrates for Rhodium-Catalyzed Alkyne Hydroacylation: Application to Dihydroquinolone Synthesis
Source: Angew Chem Int Ed Engl. 2013 Nov 12;52(50):13280–3. doi: 10.1002/anie.201308127 (PMC4227557; doi:10.1002/anie.201308127)

Angewandte Chemie
Eine Zeitschrift der Gesellschaft Deutscher Chemiker

Supporting Information

© Wiley-VCH 2013

69451 Weinheim, Germany

2-Aminobenzaldehydes as Versatile Substrates for Rhodium-Catalyzed Alkyne Hydroacylation: Application to Dihydroquinolone Synthesis**

*Matthias Castaing, Sacha L. Wason, Beatriz Estepa, Joel F. Hooper, and Michael C. Willis**

anie_201308127_sm_miscellaneous_information.pdf

Contents

I. General Considerations S3

II. General Procedure A for the preparation of 2-aminobenzaldehydes S4

III. General Procedure B for hydroacylation S10

IV. General Procedures C and D for cyclization S20

V. References S31

VI. Spectral Data S32

General Considerations

Reactions were conducted in oven-dried glassware, in anhydrous solvents with continuous magnetic stirring under an inert nitrogen atmosphere, unless otherwise stated. Glassware was dried at >200 °C for a minimum of 16 h prior to use and allowed to cool to room temperature under a flow of nitrogen. Cooling of reaction mixtures to 0 °C was achieved using an icewater bath. Cooling to -78 °C was achieved using a dry ice-acetone bath. All inorganic bases were dried in a vacuum drying pistol (120 °C, 10 mbar) for 16 h prior to use and subsequently stored under nitrogen.

Analytical thin layer chromatography was carried out using pre-coated aluminium backed silica plates (Merck Kieselgel 60F254). Plates were visualised under ultraviolet light (254 nm) and/or by staining with potassium permanganate. Flash column chromatography was carried out using

Apollo scientific silica gel 60 (0.040 – 0.063 nm). Nitrogen pressure was applied at the column head.

^{1}H and ^{13}C nuclear magnetic resonance experiments were carried out using Bruker DQX-400, AVN-400 or AVC-500 spectrometers. Chemical shifts (**δ**) are given in parts per million (ppm) and coupling constants (**J**) in Hertz (Hz). Proton multiplicities are assigned using the following abbreviations: singlet (s), doublet (d), triplet (t), quartet (q), quintet (qu), multiplet (m),

broad (br) and apparent (app). Low resolution mass spectra were recorded using a Fisons Platform spectrometer (ESI). High resolution mass spectra were recorded using a Bruker MicroTOF spectrometer by the internal service at the University of Oxford. Melting points were determined using a Leica Galen III hot-stage microscope. Infrared measurements (neat, thin film) were carried out using a Bruker Tensor 27 FT-IR with internal calibration in the range 4000-600 cm^{-1}.

Dry THF, DCM and toluene were collected fresh from an in-house Innovative Technology Inc. PS-400-7 solvent purification system having been passed through anhydrous alumina

columns. Acetone was distilled from Drierite and stored over 3 Å molecular sieves. All other solvents were used as purchased from Rathburn or

Fischer at HPLC grade. Petroleum ether refers to light petroleum boiling in the range 40-60 °C. All chemicals were purchased from Sigma Aldrich or Alfa Aesar and used without further purification, with the exception of alkynes, which were purified via distillation prior to use.

3,4-Dimethoxy-*N*-methylbenzylamine (S1)

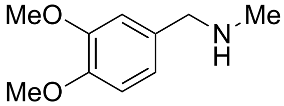

Prepared following a procedure adapted from Philippe *et al.*[1] To a stirring solution of veratraldehyde (3.66 g, 22.0 mmol) in methanol (50 mL) was added methylamine (40% aq, 2.44 mL, 28.0 mmol). After stirring at room temperature for 15 minutes, the solution was cooled down to 0 °C and sodium borohydride (0.410 g, 11.0 mmol) was added portionwise. The resulting solution was left to stir at room temperature for 1 h. Water (50 mL) was added, and the methanol was evaporated under reduced pressure. The resulting aqueous phase was extracted with DCM (3 × 50 mL), then the combined organic layers were dried over magnesium sulfate and the solvent removed *in vacuo*, affording the title compound as a colourless oil (1.85 g; 93%). The product was used in the next step without further purification. ^{1}H NMR (400 MHz, $CDCl_3$): δ = 6.80 (s, 1H, *Ar-H*), 6.76–6.71 (m, 2H, *Ar-H*), 3.79 (s, 3H, OCH_3), 3.77 (s, 3H, OCH_3), 3.59 (s, 2H, NCH_2Ph), 2.36 (s, 3H, NCH_3), 1.37 (br s, 1H, N*H*). ^{13}C NMR (100 MHz, $CDCl_3$): δ = 148.6, 147.6, 132.5, 119.9, 111.0, 110.6, 55.6, 55.5, 55.4, 35.7. LRMS (ESI): *m/z* 182 (100%, $[M+H]^+$). Data consistent with previously reported values.[2]

GENERAL PROCEDURE A: MAKING 2-AMINOBENZALDEHYDES

2-(Pyrrolidin-1-yl)benzaldehyde (8a)

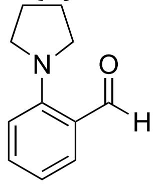

Potassium carbonate (2.8 g, 20 mmol) and pyrrolidine (1.7 mL, 20 mmol) were added to 2-fluorobenzaldehyde (1.4 mL, 16 mmol) in DMF (12 mL), and the solution was heated to 85°C under N_2. After 48 hours, the reaction was cooled down to room temperature and quenched with a saturated aqueous solution of potassium carbonate (20 mL). The water phase was extracted with DCM (3 × 20 mL) and the combined organic layers were washed with lithium chloride (3 × 20 mL) then dried over magnesium sulfate. The solvent was removed *in vacuo*, and flash chromatography (10% Et_2O in petrol) afforded the title compound as an orange oil (1.94 g; 69%). ^{1}H NMR (400 MHz, $CDCl_3$): δ = 10.10 (s, 1H, C*H*O), 7.71 (dd, *J* = 8 Hz, *J* = 2 Hz, 1H, *Ar-H*), 7.39 (ddd, *J* = 8.5 Hz, *J* = 7 Hz, *J* = 2 Hz, 1H, *Ar-H*), 6.86–6.79 (m, 2H, *Ar-H*), 3.37 (dt, *J* = 6.5 Hz, *J* = 4 Hz, 4H, N(CH_2CH_2)$_2$), 2.00 (dt, *J* = 6.5 Hz, *J* = 3.5 Hz, 4H, N(CH_2CH_2)$_2$). ^{13}C NMR (100 MHz, $CDCl_3$): δ = 190.1, 149.9, 134.2, 133.1, 123.0, 116.4, 114.5, 52.7, 25.9. IR: ν_{max} (cm^{-1}) 2970, 2871, 1676, 1599, 1478. Data consistent with previously reported values.[3]

2-(Dimethylamino)benzaldehyde (8b)

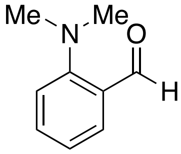

Prepared following general procedure A from 2-fluorobenzaldehyde (0.85 mL, 8.0 mmol), dimethylamine (40% aq, 1.7 mL, 10 mmol), potassium carbonate (1.4 g, 10 mmol) and DMF (8 mL). Flash chromatography (10% Et_2O in petrol) afforded the title compound as a dark yellow liquid (1.09 g; 91%). ^{1}H NMR (400 MHz, $CDCl_3$): δ = 10.25 (s, 1H, C*H*O), 7.79 (dd, *J* = 7.5 Hz, *J* = 2 Hz, 1H, *Ar-H*), 7.48 (ddd, *J* = 8.5 Hz, *J* = 7.5 Hz, *J* = 2 Hz, 1H, *Ar-H*), 7.09–6.98 (m, 2H, *Ar-H*), 2.94 (s, 6H, N(C*H*$_3$)$_2$). ^{13}C NMR (100 MHz, $CDCl_3$): δ = 190.7, 157.4, 134.3, 130.5, 126.6, 120.3, 117.3, 45.2. LRMS (ESI): *m/z* 150 (100%, $[M+H]^+$), 182 (75%, $[M+MeOH+H]^+$). Data consistent with previously reported values.[4]

2-(Benzyl(methyl)amino)benzaldehyde (8c)

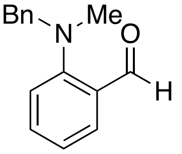

Prepared following general procedure A from 2-fluorobenzaldehyde (1.7 mL, 16. mmol), *N*-(methyl)benzylamine (2.6 mL, 20 mmol), potassium carbonate (2.8 g, 20 mmol) and DMF (15 mL). Flash chromatography (10% Et_2O in petrol) afforded the title compound as a yellow oil (2.88 g; 80%). ^{1}H NMR (400 MHz, $CDCl_3$): δ = 10.39 (s, 1H, C*H*O), 7.92–7.80 (m, 1H, *Ar-H*), 7.66–7.54 (m, 1H, *Ar-H*), 7.54–7.44 (m, 1H, *Ar-H*), 7.36–7.25 (m, 4H, *Ar-H*), 7.22–7.04 (m, 2H, *Ar-H*), 4.34 (s, 2H, NC*H*$_2$), 2.82 (s, 3H, NC*H*$_3$). ^{13}C NMR (100 MHz, $CDCl_3$): δ = 191.3, 155.7, 137.4, 136.3, 134.7, 130.1, 128.5, 128.0, 127.4, 121.6, 119.5, 62.3, 42.3. IR: ν_{max} (cm^{-1}) 2855, 1683, 1596, 1482, 1274. Data is consistent with previously reported values.[5]

2-((3,4-Dimethoxybenzyl)(methyl)amino)benzaldehyde (8d)

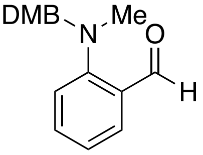

Prepared following general procedure A from 2-fluorobenzaldehyde (1.7 mL, 16. mmol), 3,4-dimethoxy-*N*-methylbenzylamine **S1** (3.6 g, 20 mmol), potassium carbonate (2.8 g, 20 mmol) and DMF (15 mL). Flash chromatography (10% Et_2O in petrol) afforded the title compound as a yellow solid (3.47 g; 76%). ^{1}H NMR (400 MHz, $CDCl_3$): δ = 10.39 (s, 1H, C*H*O), 7.82 (dd, *J* = 7.5 Hz, *J* = 2 Hz, 1H, *Ar-H*), 7.48 (ddd, *J* = 8 Hz, *J* = 7.5 Hz, *J* = 2 Hz, 1H, *Ar-H*), 7.07 (app d, *J* = 8 Hz, 2H, *Ar-H*), 6.81 (app d, *J* = 1 Hz, 2H, *Ar-H*), 6.73 (s, 1H, *Ar-H*), 4.27 (s, 2H, NC*H*$_2$Ph), 3.87 (s, 3H, OC*H*$_3$), 3.80 (s, 3H, OC*H*$_3$), 2.80 (s, 3H, NC*H*$_3$). ^{13}C NMR (100 MHz, $CDCl_3$): δ = 191.2, 155.5, 148.9, 148.3, 134.6, 130.2, 129.7, 128.1, 121.6, 120.3, 119.6, 111.0, 110.9, 62.2, 55.8, 55.7, 42.0. IR: ν_{max} (cm^{-1}) 2835, 2360, 1684, 1595, 1515, 1453, 1261, 1139, 1028, 765. LRMS (ESI): *m/z* 308 (100%, $[M+Na]^+$), 593 (80%, $[2M+Na]^+$). HRMS (ESI): Found 308.1252 ($M+Na^+$), calculated for $C_{17}H_{19}NNaO_3$ is 308.1257. M.p. 55–57 °C (CH_2Cl_2/petrol).

2-(Methylamino)benzaldehyde (8e)

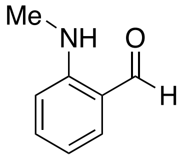

Prepared following the procedure described by Baum *et al.*[6] Anhydrous $ZnCl_2$ (1.70 g, 12.5 mmol) was stirred in THF (35 mL) under nitrogen, and the mixture was cooled to 0 °C. MeLi (1.6 M in Et_2O, 6.3 mL, 10 mmol), was added slowly, and the mixture allowed to warm to RT. 2,1-benzisoxazole (0.60 g, 10 mmol) in THF (5 mL) was added, followed by $Ni(acac)_2$ (26 mg, 0.20 mmol) in THF (5 mL). After 3 h stirring at RT, the mixture was quenched by the addition of 6 M HCl (3 mL) and poured into water (50 mL). The aqueous phase was extracted with Et_2O (3 × 20 mL) and the combined organic layers were washed with brine (1 × 20 mL) then dried over magnesium sulfate. The solvent was removed *in vacuo*, and flash chromatography (10% Et_2O in petrol) afforded the title compound as a yellow oil (490 mg; 73%). ^1H NMR (400 MHz, $CDCl_3$): δ = 9.81 (s, 1H, C*H*O), 8.25, (br s, 1H, N*H*), 7.45 (dd, *J* = 7.5 Hz, *J* = 1.5 Hz, 1H, *Ar-H*), 7.41 (ddd, *J* = 8 Hz, *J* = 7.5 Hz, *J* = 1.5 Hz, 1H, *Ar-H*), 6.70–6.65 (m, 2H, *Ar-H*), 2.92 (d, *J* = 5 Hz, 3H, NCH_3). ^{13}C NMR (100 MHz, $CDCl_3$): δ = 193.8, 151.5, 136.5, 135.7, 118.3, 114.6, 110.3, 29.0. LRMS (ESI): *m/z* 136 (100%, $[M+H]^+$). Data consistent with previously reported values.[6]

2-Aminobenzaldehyde (8f)

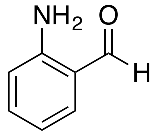

2-Aminobenzyl alcohol (1.23 g, 10.0 mmol) was dissolved in DCM (25 mL). Manganese(IV) oxide (1.82 g, 21.0 mmol) was added under nitrogen and the solution was left to stir at RT for 48 h. Manganese oxide was filtered off and the resulting filtrate was concentrated under vacuum. Flash chromatography (100% DCM) afforded the title compound as a yellow solid (1.08 g; 89%). ^1H NMR (400 MHz, $CDCl_3$): δ = 9.87 (s, 1H, C*H*O), 7.48 (dd, *J* = 8 Hz, *J* = 1.5 Hz, 1H, *Ar-H*), 7.32 (ddd, *J* = 8.5 Hz, *J* = 7 Hz, *J* = 1.5 Hz, 1H, *Ar-H*), 6.75 (ddd, *J* = 8 Hz, *J* = 7 Hz, *J* = 1.0 Hz, 1H, *Ar-H*), 6.66 (app d, *J* = 8.5 Hz, 1H, *Ar-H*), 6.19 (br s, 2H, NH_2). ^{13}C NMR (100 MHz, $CDCl_3$): δ = 193.9, 149.8, 135.6, 135.1, 118.6, 116.2, 115.9. LRMS (ESI): *m/z* 122 (100%, $[M+H]^+$). Data consistent with previously reported values.[7]

2-((3,4-Dimethoxybenzyl)(methyl)amino)-3-methoxybenzaldehyde (8g)

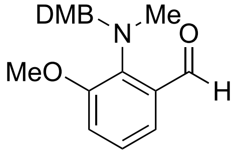

Prepared following general procedure A from 2-fluoro-3-methoxybenzaldehyde (0.46 g, 3.0 mmol), 3,4-dimethoxy-*N*-methylbenzylamine **S1** (0.72 g, 4.0 mmol), potassium carbonate (0.55 g, 4.0 mmol) and DMF (3 mL). Flash chromatography (10% Et_2O in petrol) afforded the title compound as a yellow solid (250 mg; 27%). ^1H NMR (400 MHz, $CDCl_3$): δ = 10.64 (s, 1H, C*H*O), 7.39 (dd, *J* = 7.5 Hz, *J* = 1.5 Hz, 1H, *Ar-H*), 7.21 (td, *J* = 8 Hz, *J* = 0.5 Hz, 1H, *Ar-H*), 7.12 (dd, *J* = 8 Hz, *J* = 1.5 Hz, 1H, *Ar-H*), 6.84–6.81 (m, 2H, *Ar-H*), 6.78 (d, *J* = 8 Hz, 1H, *Ar-H*), 4.23 (s, 2H,

NCH_2), 3.93 (s, 3H, OCH_3), 3.87 (s, 3H, OCH_3), 3.84 (s, 3H, OCH_3), 2.84 (s, 3H, NCH_3). ^{13}C NMR (100 MHz, $CDCl_3$): δ = 193.4, 158.7, 148.7, 148.0, 144.2, 134.8, 131.9, 126.0, 120.8, 119.4, 117.0, 111.9, 110.7, 60.0, 55.8, 55.7, 55.4, 42.2. IR: ν_{max} (cm^{-1}) 2835, 1687, 1606, 1549, 1513, 1465, 1418, 1261, 1184, 1138, 1026, 975, 854, 785, 724. LRMS (ESI): *m/z* 316 (100%, M+H]$^+$), 348 (95%, [M+MeOH+H]$^+$). HRMS (ESI): Found 338.1368 (M+Na$^+$), calculated for $C_{18}H_{21}NNaO_4$ is 338.1363. M.p. 75–77 °C (CH_2Cl_2/petrol).

2-((3,4-Dimethoxybenzyl)(methyl)amino)-4,5-dimethoxybenzaldehyde (8h)

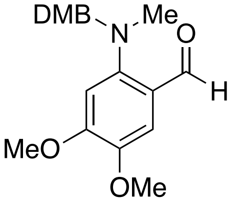

Prepared following general procedure A from 2-fluoro-4,5-dimethoxybenzaldehyde (0.37 g, 2.0 mmol), 3,4-dimethoxy-*N*-methylbenzylamine **S1** (0.45 g, 2.5 mmol), potassium carbonate (0.35 g, 2.5 mmol) and DMF (2 mL). Flash chromatography (10% Et_2O in petrol) afforded the title compound as a yellow oil (250 mg; 36%). ^{1}H NMR (400 MHz, $CDCl_3$): δ = 10.33 (s, 1H, C*H*O), 7.26 (s, 1H, *Ar-H*), 6.73 (s, 2H, *Ar-H*), 6.70 (s, 1H, *Ar-H*), 6.55 (s, 1H, *Ar-H*), 4.12 (s, 2H, NCH_2), 3.84 (s, 3H, OCH_3), 3.81 (s, 3H, OCH_3), 3.78 (s, 3H, OCH_3), 3.74 (s, 3H, OCH_3), 2.70 (s, 3H, NCH_3). ^{13}C NMR (100 MHz, $CDCl_3$): δ = 189.5, 154.4, 152.2, 148.7, 148.1, 144.9, 129.7, 121.9, 120.3, 111.1, 110.7, 109.4, 103.3, 62.6, 55.8 (2C), 55.6, 55.5, 42.8. IR: ν_{max} (cm^{-1}) 2847, 2360, 1683, 1592, 1515, 1461, 1411, 1261, 1139, 1028, 764. LRMS (ESI): *m/z* 368 (75%, [M+Na]$^+$), 713 (100%, [2M+Na]$^+$). HRMS (ESI): Found 368.1459 (M+Na$^+$), calculated for $C_{19}H_{23}NNaO_5$ is 368.1468.

2-((3,4-Dimethoxybenzyl)(methyl)amino)-6-methoxybenzaldehyde (8i)

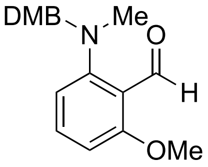

Prepared following general procedure A from 2-fluoro-6-methoxybenzaldehyde (0.46 g, 3.0 mmol), 3,4-dimethoxy-*N*-methylbenzylamine **S1** (0.72 g, 4.0 mmol), potassium carbonate (0.55 g, 4.0 mmol) and DMF (3 mL). Flash chromatography (10% Et_2O in petrol) afforded the title compound as a yellow oil (925 mg; 98%). ^{1}H NMR (400 MHz, $CDCl_3$): δ = 10.44 (s, 1H, C*H*O), 7.37 (t, *J* = 8.5 Hz, 1H, *Ar-H*), 6.82–6.76 (m, 3H, *Ar-H*), 6.62 (d, *J* = 8.5 Hz, 1H, *Ar-H*), 6.52 (d, *J* = 8 Hz, 1H, *Ar-H*), 4.31 (s, 2H, NCH_2), 3.92 (s, 3H, OCH_3), 3.87 (s, 3H, OCH_3), 3.82 (s, 3H, OCH_3), 2.77 (s, 3H, NCH_3). ^{13}C NMR (100 MHz, $CDCl_3$): δ = 188.9, 162.9, 155.3, 148.9, 148.0, 134.8, 130.1, 120.1, 116.1, 111.3, 111.0, 110.7, 102.7, 60.7, 55.9, 55.8, 55.7, 41.8. IR: ν_{max} (cm^{-1}) 2836, 1666, 1591, 1560, 1514, 1467, 1410, 1260, 1137, 1072, 1028, 816, 782, 726. LRMS (ESI): *m/z* 316 (100%, M+H]$^+$), 348 (85%, [M+MeOH+H]$^+$). HRMS (ESI): Found 338.1353 (M+Na$^+$), calculated for $C_{18}H_{21}NNaO_4$ is 338.1363.

2-((3,4-Dimethoxybenzyl)(methyl)amino)-4-trifluoromethylbenzaldehyde (8j)

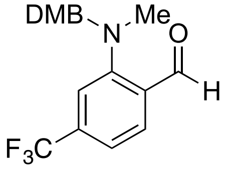

Prepared following general procedure A from 2-fluoro-4-(trifluoromethyl)benzaldehyde (0.38 g, 2.0 mmol), 3,4-dimethoxy-*N*-methylbenzylamine **S1** (0.45 g, 2.5 mmol), potassium carbonate (0.35 g, 2.5 mmol) and DMF (2 mL). Flash chromatography (10% Et_2O in petrol) afforded the title compound as a yellow solid (552 mg, 78%). ^1H NMR (400 MHz, $CDCl_3$): δ = 10.34 (s, 1H, C*H*O), 7.89 (d, *J* = 8 Hz, 1H, *Ar-H*), 7.31 (s, 1H, *Ar-H*), 7.28 (d, *J* = 8.5 Hz, 1H, *Ar-H*), 6.83 (d, *J* = 8 Hz, 1H, *Ar-H*), 6.80 (dd, *J* = 8 Hz, *J* = 1.5 Hz, 1H, *Ar-H*), 6.70 (d, *J* = 1.5 Hz, 1H, *Ar-H*), 4.32 (s, 2H, NCH_2), 3.87 (s, 3H, OCH_3), 3.80 (s, 3H, OCH_3), 2.75 (s, 3H, NCH_3). ^{13}C NMR (100 MHz, $CDCl_3$): δ = 190.2, 155.3, 149.2, 148.6, 135.7 (q, $^2J_{CF_3}$ = 32 Hz), 131.1, 129.9, 129.1, 123.6 (q, $^1J_{CF_3}$ = 273 Hz), 120.3, 117.7 (q, $^3J_{CF_3}$ = 4 Hz), 116.3 (q, $^3J_{CF_3}$ = 4 Hz), 111.1, 110.8, 62.1, 55.9, 55.8, 41.9. IR: ν_{max} (cm^{-1}) 2847, 2360, 1683, 1592, 1515, 1461, 1411, 1261, 1139, 1028, 764. LRMS (ESI): *m/z* 376 (100%, $[M+Na]^+$). HRMS (ESI): Found 376.1130 ($M+Na^+$), calculated for $C_{18}H_{18}F_3NNaO_3$ is 376.1131. M.p. 79–81°C (CH_2Cl_2/petrol).

4-Bromo-2-((3,4-dimethoxybenzyl)(methyl)amino)benzaldehyde (8k)

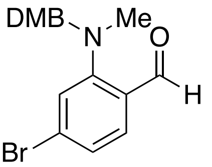

Prepared following general procedure A from 2-fluoro-4-bromobenzaldehyde (0.61 g, 3.0 mmol), 3,4-dimethoxy-*N*-methylbenzylamine **S1** (0.72 g, 4.0 mmol), potassium carbonate (0.55 g, 4.0 mmol) and DMF (3 mL). Flash chromatography (10% Et_2O in petrol) afforded the title compound as an off-white solid (1.04 g; 95%). ^1H NMR (400 MHz, $CDCl_3$): δ = 10.24 (s, 1H, C*H*O), 7.62 (d, *J* = 8.5 Hz, 1H, *Ar-H*), 7.19 (d, *J* = 1.5 Hz, 1H, *Ar-H*), 7.14 (ddd, *J* = 8.5 Hz, *J* = 2 Hz, *J* = 0.5 Hz, 1H, *Ar-H*), 6.80 (d, *J* = 8 Hz, 1H, *Ar-H*), 6.77 (dd, *J* = 8 Hz, *J* = 2 Hz, 1H, *Ar-H*), 6.70 (d, *J* = 2 Hz, 1H, *Ar-H*), 4.25 (s, 2H, NCH_2), 3.84 (s, 3H, OCH_3), 3.79 (s, 3H, OCH_3), 2.77 (s, 3H, NCH_3). ^{13}C NMR (100 MHz, $CDCl_3$): δ = 189.7, 155.8, 148.9, 148.3, 131.6, 129.4, 129.0, 126.3, 124.3, 122.5, 120.0, 110.9, 110.7, 61.8, 55.7, 55.6, 41.5. IR: ν_{max} (cm^{-1}) 2835, 2345, 1675, 1580, 1514, 1451, 1259, 1235, 1182, 1137, 1081, 1026, 916, 840, 807, 766, 729, 668, 645. LRMS (ESI): *m/z* 386 (95%, [[^{79}Br] $M+Na]^+$), 388 (100%, [[^{81}Br] $M+Na]^+$), 396 (85%, [[^{79}Br] $M+MeOH+H]^+$), 398 (85%, [[^{81}Br] $M+MeOH+H]^+$). HRMS (ESI): Found 386.0367 ([^{79}Br] $M+Na^+$), 388.0348 ([^{81}Br] $M+Na^+$), calculated for $C_{17}H_{18}{}^{79}BrNNaO_3$ is 386.0362, calculated for $C_{17}H_{18}{}^{81}BrNNaO_3$ is 388.0343. M.p. 91–92 °C (CH_2Cl_2/petrol).

3-((3,4-Dimethoxybenzyl)(methyl)amino)thiophene-2-carbaldehyde (8l)

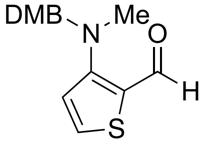

Prepared following general procedure A from 3-bromothiophene-2-carboxaldehyde (0.57 mg, 3.0 mmol), 3,4-dimethoxy-*N*-methylbenzylamine **S1** (0.72 g, 4.0 mmol), potassium carbonate (0.55 g, 4.0 mmol) and DMF (3 mL). Flash chromatography (10% Et_2O in petrol) afforded the title compound as a brown solid (883 mg; 95%). ^{1}H NMR (400 MHz, $CDCl_3$): δ = 9.84 (s, 1H, C*H*O), 7.53 (dd, *J* = 5.5 Hz, *J* = 1 Hz, 1H, *Ar-H*), 6.84 (d, *J* = 8.0 Hz, 1H, *Ar-H*), 6.77–6.73 (m, 1H, *Ar-H*), 6.71–6.68 (m, 2H, *Ar-H*), 4.62 (s, 2H, NC*H$_2$*), 3.87 (s, 3H, OC*H$_3$*), 3.84 (s, 3H, OC*H$_3$*), 3.21 (s, 3H, NC*H$_3$*). ^{13}C NMR (100 MHz, $CDCl_3$): δ = 180.9, 156.0, 149.5, 148.5, 136.0, 128.8, 120.0, 119.4, 118.6, 111.4, 109.6, 60.1, 55.9(3), 55.9(0), 42.8. IR: ν_{max} (cm^{-1}) 2934, 1605, 1544, 1505, 1414, 1377, 1257, 1236, 1164, 1138, 1027, 807, 750, 665. LRMS (ESI): *m/z* 314 (100%, [M+Na]$^+$). M.p. 83–84°C (CH_2Cl_2/petrol).

***(E)*-1-(2-(Pyrrolidin-1-yl)phenyl)hept-2-en-1-one (9a)**

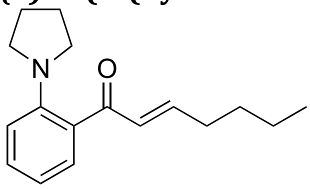

[Rh(nbd)$_2$]BF_4 (5.6 mg, 0.015 mmol) and dcpm (6.1 mg, 0.015 mmol) were dissolved in distilled acetone (1.5 mL). Hydrogen gas was then bubbled through the solution for 2 minutes and purged with nitrogen for 30 seconds. To this solution was added 2-(pyrrolidin-1-yl)benzaldehyde **8a** (25.9 mg, 0.15 mmol) and 1-hexyne (26 µL, 0.225 mmol) in distilled acetone (0.5 mL). The solution was heated at 55 °C for 30 minutes. Ether (5 mL) was then added and the solution filtered through a silica plug, and then concentrated *in vacuo*. Flash chromatography (10% Et2O in petrol) afforded the title compound as a bright yellow oil (32 mg, 82%). ^{1}H NMR (400 MHz, $CDCl_3$): δ = 7.37 (dd, *J* = 7.5 Hz, *J* = 1.5 Hz, 1H, *Ar-H*), 7.31 (ddd, *J* = 8.5 Hz, *J* = 7.0 Hz, *J* = 1.5 Hz, 1H, *Ar-H*), 6.86 (dt, *J* = 15.5 Hz, *J* = 7.0 Hz, 1H, COC*H*), 6.79 (1H, d, *J* = 8.5 Hz, *Ar-H*), 6.75–6.69 (m, 1H, *Ar-H*), 6.53 (dt, *J* = 15.5 Hz, *J* = 1.5 Hz, 1H, COCHC*H*), 3.15 (dt, *J* = 6.5 Hz, *J* = 4.0 Hz, 4H, (CH_2C*H$_2$*)$_2$N), 2.28 (ddd, *J* = 14.5 Hz, *J* = 7.0 Hz, *J* = 1.5 Hz, 2H, COCHCHC*H*$_2$), 1.93 (dt, *J* = 6.5, 3.5 Hz, 4H, (C*H$_2$*CH_2)$_2$N), 1.52–1.26 (m, 4H, (C*H$_2$*)$_2$$CH_3$), 0.93 (t, *J* = 7.5 Hz, 3H, (CH_2)$_2$C*H$_3$*). ^{13}C NMR (100 MHz, $CDCl_3$): δ = 195.7, 149.8, 147.5, 131.2, 130.8, 130.3, 125.7, 115.3, 113.9, 51.2, 32.2, 30.2, 25.8, 22.3, 13.8. IR: ν_{max} (cm^{-1}) 2959, 2929, 2870, 1647, 1597, 1446. LRMS (ESI): *m/z* 258 (35%, [M+H]$^+$), 280 (25%, [M+Na]$^+$), 537 (100%, [2M+Na]$^+$). HRMS (ESI): Found 258.1848 (M+H$^+$), calculated for $C_{17}H_{24}NO$ is 258.1852.

GENERAL PROCEDURE B: MAKING 2-AMINO ARYL ENONES

(*E*)-3-Phenyl-1-(2-(pyrrolidin-1-yl)phenyl)prop-2-en-1-one (9b)

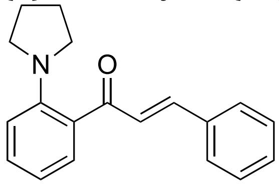

$[Rh(nbd)_2]BF_4$ (9.4 mg, 25 µmol) and dcpm (10.2 mg, 25 µmol) were dissolved in distilled acetone (3 mL), using a sonicator to dissolve if necessary. Hydrogen gas was then bubbled through the solution until a colour change was observed (1–2 minutes) and purged with nitrogen for 30 seconds. MeCN (2.5 µL, 0.050 mmol) in DCE (122.5 µL) was added, and this solution was added to a separate microwave tube containing 2-(pyrrolidin-1-yl)benzaldehyde **8a** (88 mg, 0.50 mmol) and phenylacetylene (85 µL, 0.75 mmol) in distilled acetone (1.0 mL). The solution was heated at 55 °C for 2 h. Flash chromatography (5% Et_2O in petrol) afforded the title compound as a dark yellow oil (127 mg; 91%). ^1H NMR (400 MHz, $CDCl_3$): δ = 7.64 (d, *J* = 15.5 Hz, 1H, C*H*Ph), 7.61–7.55 (m, 2H, *Ar-H*), 7.49 (d, *J* = 7.5 Hz, 1H, *Ar-H*), 7.44–7.34 (m, 4H, *Ar-H*), 7.23 (d, *J* = 15.5 Hz, 1H, COC*H*), 6.86 (d, *J* = 8.5 Hz, 1H, *Ar-H*), 6.78 (t, *J* = 7.5 Hz, 1H, *Ar-H*), 3.26–3.15 (m, 4H, $N(CH_2CH_2)_2$), 1.93 (dt, *J* = 6.5 Hz, *J* = 3.5 Hz, 4H, $N(CH_2CH_2)_2$). ^{13}C NMR (100 MHz, $CDCl_3$): δ = 194.8, 153.4, 144.1, 134.9, 133.0, 131.5, 130.2, 128.9, 128.3, 127.1, 126.0, 115.6, 114.1, 51.5, 25.9. IR: ν_{max} (cm^{-1}) 2969, 2869, 1656, 1445, 740. LRMS (ESI): *m/z* 278 (50%, $[M+H]^+$), 316 (100%, $[M+K]^+$), 593 (80%, $[2M+K]^+$). HRMS (ESI): Found 278.1538 ($M+H^+$), calculated for $C_{19}H_{20}NO$ is 278.1539.

(*E*)-1-(2-(Dimethylamino)phenyl)-3-phenylprop-2-en-1-one (9c)

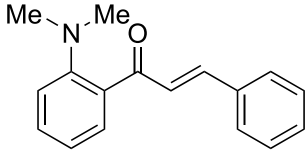

Prepared following general procedure B from 2-(dimethylamino)benzaldehyde **8b** (75 mg, 0.50 mmol), phenylacetylene (85 µL, 0.75 mmol), $[Rh(nbd)_2]BF_4$ (9.4 mg, 25 µmol), dcpm (10.2 mg, 25 µmol), MeCN (2% in DCE, 125 µL, 50 µmol) in acetone (4 mL). Flash chromatography (10% Et_2O in petrol) afforded the title compound as a dark yellow oil (122 mg; 97%). ^1H NMR (400 MHz, $CDCl_3$): δ = 7.78 (d, *J* = 16 Hz, 1H, C*H*Ph), 7.62–7.58 (m, 2H, *Ar-H*), 7.43 (d, *J* = 16 Hz, 1H, COC*H*) and 7.44–7.39 (m, 5H, *Ar-H*) overlapping, 7.06 (d, *J* = 8.5 Hz, 1H, *Ar-H*), 6.99 (t, *J* = 7.5 Hz, 1H, *Ar-H*), 2.85 (s, 6H, $N(CH_3)_2$). ^{13}C NMR (100 MHz, $CDCl_3$): δ = 194.6, 152.0, 142.2, 134.9, 131.7, 131.3, 130.2, 129.9, 128.6, 128.0, 125.8, 119.8, 116.6, 44.1. LRMS (ESI): *m/z* 252 (100%, $[M+H]^+$). Data consistent with previously reported values.[8]

(*E*)-1-(2-(Benzyl(methyl)amino)phenyl)-3-phenylprop-2-en-1-one (9d)

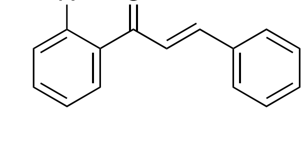

Prepared following general procedure B from 2-(benzyl(methyl)amino)benzaldehyde **8c** (112 mg, 0.5 mmol), phenylacetylene (85 μL, 0.75 mmol), $[Rh(nbd)_2]BF_4$ (9.4 mg, 25 μmol), dcpm (10.2 mg, 25 μmol), MeCN (2% in DCE, 125 μL, 50 μmol) in acetone (4 mL). Flash chromatography (10% Et_2O in petrol) afforded the title compound as a yellow solid (156 mg; 95%). ^1H NMR (400 MHz, $CDCl_3$): δ = 7.72 (d, *J* = 16 Hz, 1H, C*H*Ph), 7.63–7.57 (m, 3H, *Ar-H*), 7.45 (d, *J* = 16 Hz, 1H, COC*H*) and 7.46–7.41 (m, 4H, *Ar-H*) overlapping, 7.22 (m, 5H, *Ar-H*), 7.11 (dd, *J* = 8 Hz, *J* = 0.5 Hz, 1H, *Ar-H*), 7.07 (td, *J* = 7.5 Hz, *J* = 1 Hz, 1H, *Ar-H*), 4.28 (s, 2H, NCH_2), 2.72 (s, 3H, NCH_3). ^{13}C NMR (100 MHz, $CDCl_3$): δ = 195.2, 151.8, 142.7, 137.5, 135.1, 132.9, 131.8 (2C), 130.3, 130.2, 128.9, 128.3, 127.2, 126.5, 121.1, 118.7, 60.6, 41.9. IR: ν_{max} (cm^{-1}) 3027, 1660, 1600, 1575, 1486, 1449, 1330, 1205, 1103, 1020, 978, 745, 699. LRMS (ESI): *m/z* 328 (35%, $[M+H]^+$), 350 (60%, $[M+Na]^+$), 677 (100%, $[2M+Na]^+$). HRMS (ESI): Found 328.1692 ($M+H^+$), calculated for $C_{23}H_{22}NO$ is 328.1696. M.p. 107–109 °C.

(E)-1-(2-((3,4-Dimethoxybenzyl)(methyl)amino)phenyl)-3-(phenyl)prop-2-en-1-one (9e)

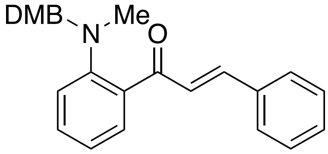

Prepared following general procedure B from 2-((3,4-dimethoxybenzyl)(methyl)amino)benzaldehyde **8d** (143 mg, 0.5 mmol), phenylacetylene (85 μL, 0.75 mmol), $[Rh(nbd)_2]BF_4$ (9.4 mg, 25 μmol), dcpm (10.2 mg, 25 μmol), MeCN (2% in DCE, 125 μL, 50 μmol) in acetone (4 mL). Flash chromatography (10% Et_2O in petrol) afforded the title compound as a yellow oil (193 mg; 99%). ^1H NMR (400 MHz, $CDCl_3$): δ = 7.70 (d, *J* = 16 Hz, 1H, C*H*Ph), 7.59–7.52 (m, 3H, *Ar-H*), 7.45 (d, *J* = 16 Hz, 1H, COC*H*) and 7.45–7.36 (m, 4H, *Ar-H*), 7.10–7.01 (m, 2H, *Ar-H*), 6.76–6.66 (m, 3H, *Ar-H*), 4.15 (s, 2H, NCH_2), 3.80 (s, 3H, OCH_3), 3.63 (s, 3H, OCH_3), 2.69 (s, 3H, NCH_3). ^{13}C NMR (100 MHz, $CDCl_3$): δ = 195.2, 151.7, 148.8, 148.1, 142.7, 135.0, 133.1, 131.7, 130.2, 129.9 (2C), 128.8, 128.2, 126.4, 121.2, 120.6, 119.0, 111.2, 110.6, 60.9, 55.7, 55.5, 41.0. IR: ν_{max} (cm^{-1}) 2935, 1659, 1597, 1514, 1449, 1421, 1330, 1260, 1237, 1139, 1028, 915, 857, 810, 761, 700. LRMS (ESI): *m/z*: 388 (70%, $[M+H]^+$), 410 (35%, $[M+Na]^+$), 797 (100%, $[2M+Na]^+$). HRMS (ESI): Found 410.1720 ($M+Na^+$), calculated for $C_{25}H_{25}NNaO_3$ is 410.1727.

(*E*)-1-(2-(Methylamino)phenyl)-3-phenylprop-2-en-1-one (9f)

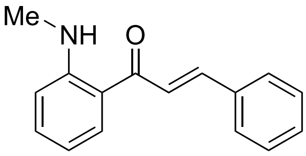

Prepared following general procedure B from 2-(methylamino)benzaldehyde **8e** (68 mg, 0.50 mmol), phenylacetylene (85 μL, 0.75 mmol), $[Rh(nbd)_2]BF_4$ (9.4 mg, 25 μmol), dcpm (10.2 mg, 25 μmol), MeCN (2% in DCE, 125 μL, 50 μmol) in acetone (4 mL). Flash chromatography (10% Et_2O in petrol) afforded the title compound as a yellow oil (88 mg; 74%). ^1H NMR (400 MHz, $CDCl_3$): δ = 9.02 (br s, 1H, N*H*), 7.92 (dd, *J* = 8 Hz, *J* = 1.5 Hz, 1H, *Ar-H*), 7.72 (d, *J* = 15.5 Hz, 1H, C*H*Ph),

7.66 (d, *J* = 15.5 Hz, 1H, COC*H*), 7.65–7.62 (m, 2H, *Ar-H*), 7.44–7.36 (m, 4H, *Ar-H*), 6.75 (dd, *J* = 8.5 Hz, *J* = 1 Hz, 1H, *Ar-H*), 6.65 (ddd, *J* = 8 Hz, *J* = 7 Hz, *J* = 1 Hz, 1H, *Ar-H*), 2.96 (s, 3H, NCH_3). ^{13}C NMR (100 MHz, $CDCl_3$): δ = 191.5, 152.7, 142.4, 135.4, 135.0, 131.6, 129.9, 128.9, 128.2, 123.3, 118.2, 114.0, 111.4, 29.4. LRMS (ESI): *m/z* 238 (100%, $[M+H]^+$), 260 (70%, $[M+Na]^+$). Data consistent with previously reported values.[9]

(*E*)-1-(2-aminophenyl)-3-phenylprop-2-en-1-one (9g)

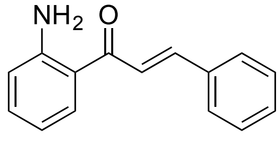

Prepared following general procedure B from 2-aminobenzaldehyde **8f** (61 mg, 0.50 mmol), phenylacetylene (85 µL, 0.75 mmol), $[Rh(nbd)_2]BF_4$ (9.4 mg, 25 µmol), dcpm (10.2 mg, 25 µmol), MeCN (2% in DCE, 125 µL, 50 µmol) in acetone (4 mL). Flash chromatography (10% Et_2O in petrol) afforded the title compound as an orange solid (102 mg; 91%). ^{1}H NMR (400 MHz, $CDCl_3$): δ = 7.79 (dd, *J* = 8.5 Hz, *J* = 1.5 Hz, 1H, *Ar-H*), 7.67 (d, *J* = 15.5 Hz, 1H, C*H*Ph), 7.58–7.52 (m, 3H, *Ar-H* and COC*H*), 7.36–7.29 (m, 3H, *Ar-H*), 7.21 (ddd, *J* = 8.5 Hz, *J* = 7 Hz, *J* = 1.5 Hz, 1H, *Ar-H*), 6.64–6.60 (m, 2H, *Ar-H*), 6.26 (br s, 2H, –NH_2). ^{13}C NMR (100 MHz, $CDCl_3$): δ = 191.6, 151.0, 142.9, 135.2, 134.3, 131.0, 130.0, 128.9, 128.2, 123.1, 119.0, 117.3, 115.8. LRMS (ESI): *m/z* 224 (100%, $[M+H]^+$), 246 (40%, $[M+Na]^+$). M.p. 70–71 °C. Data consistent with previously reported values.[10]

(*E*)-1-(2-((3,4-Dimethoxybenzyl)(methyl)amino)-3-methoxyphenyl)-3-phenylprop-2-en-1-one (9h)

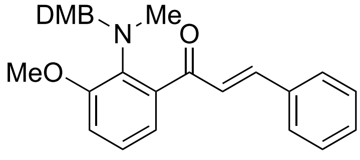

Prepared following general procedure B from 2-((3,4-dimethoxybenzyl)(methyl)amino)-3-methoxybenzaldehyde **8g** (158 mg, 0.50 mmol), phenylacetylene (85 µL, 0.75 mmol), $[Rh(nbd)_2]BF_4$ (9.4 mg, 25 µmol), dcpm (10.2 mg, 25 µmol), MeCN (2% in DCE, 125 µL, 50 µmol) in acetone (4 mL). Flash chromatography (10% Et_2O in petrol) afforded the title compound as a yellow oil (191 mg; 92%). ^{1}H NMR (400 MHz, $CDCl_3$): δ = 7.52–7.49 (m, 2H, *Ar-H*), 7.43 (d, *J* = 16 Hz, 1H, C*H*Ph), 7.41–7.35 (m, 3H, *Ar-H*), 7.22 (dd, *J* = 8 Hz, *J* = 7.5 Hz, 1H, *Ar-H*), 7.11 (d, *J* = 16 Hz, 1H, COC*H*), 7.03 (dd, *J* = 8 Hz, *J* = 1.5 Hz, 1H, *Ar-H*), 6.98 (dd, *J* = 7.5 Hz, *J* = 1.5 Hz, 1H, *Ar-H*), 6.91 (d, *J* = 2 Hz, 1H, *Ar-H*), 6.81 (dd, *J* = 8 Hz, *J* = 2 Hz, 1H, *Ar-H*), 6.69 (d, *J* = 8 Hz, 1H, *Ar-H*), 4.11 (s, 2H, NCH_2), 3.94 (s, 3H, OCH_3), 3.82 (s, 3H, OCH_3), 3.76 (s, 3H, OCH_3), 2.70 (s, 3H, NCH_3). ^{13}C NMR (100 MHz, $CDCl_3$): δ = 196.9, 158.3, 148.7, 147.7, 143.5, 140.6, 139.3, 134.7, 132.0, 130.2, 128.8, 128.1, 127.8, 125.8, 120.6, 119.7, 113.3, 111.6, 110.4, 60.0, 55.7, 55.5, 55.3, 40.2. IR: ν_{max} (cm^{-1}) 2943, 1648, 1602, 1576, 1513, 1464, 1418, 1312, 1259, 1234, 1139, 1074, 1029, 978, 912, 861, 806, 763, 730, 685, 647. LRMS (ESI): *m/z* 418 (100%, $[M+H]^+$). HRMS (ESI): Found 440.1837 ($M+Na^+$), calculated for $C_{26}H_{27}NNaO_4$ is 440.1832.

(E)-1-(2-((3,4-Dimethoxybenzyl)(methyl)amino)-4,5-dimethoxyphenyl)-3-phenylprop-2-en-1-one (9i)

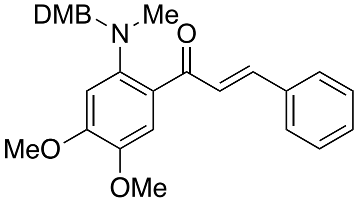

Prepared following general procedure B from 2-((3,4-dimethoxybenzyl)(methyl)amino)-4,5-dimethoxybenzaldehyde **8h** (173 mg, 0.50 mmol), phenylacetylene (85 µL, 0.75 mmol), [Rh(nbd)$_2$]BF$_4$ (9.4 mg, 25 µmol), dcpm (10.2 mg, 25 µmol), MeCN (2% in DCE, 125 µL, 50 µmol) in acetone (4 mL). Flash chromatography (10% Et$_2$O in petrol) afforded the title compound as a yellow oil (219 mg; 98%). ^{1}H NMR (400 MHz, CDCl$_3$): δ = 7.74 (d, *J* = 16 Hz, 1H, C*H*Ph), 7.67 (d, *J* = 16 Hz, 1H, COC*H*), 7.54–7.52 (m, 2H, *Ar-H*), 7.38–7.33 (m, 3H, *Ar-H*), 7.24 (s, 1H, *Ar-H*), 6.72–6.60 (m, 4H, *Ar-H*), 4.07 (s, 2H, NC*H$_2$*), 3.89 (s, 3H, OC*H$_3$*), 3.88 (s, 3H, OC*H$_3$*), 3.80 (s, 3H, OC*H$_3$*), 3.60 (s, 3H, OC*H$_3$*), 2.68 (s, 3H, NC*H$_3$*). ^{13}C NMR (100 MHz, CDCl$_3$): δ = 192.7, 152.1, 148.7, 148.1, 147.7, 144.3, 141.2, 135.3, 129.8, 129.7, 128.8, 128.1, 126.6, 125.9, 121.0, 112.8, 111.6, 110.6, 103.4, 62.1, 56.0, 55.9, 55.7, 55.5, 41.8. IR: ν_{max} (cm^{-1}) 2950, 1655, 1592, 1509, 1455, 1415, 1261, 1239, 1163, 828, 765. LRMS (ESI): *m/z*: 448 (30%, [M+H]$^+$), 470 (100%, [M+Na]$^+$). HRMS (ESI): Found 470.1927 (M+Na$^+$), calculated for $C_{27}H_{29}NNaO_5$ is 470.1937.

(*E*)-1-(2-((3,4-Dimethoxybenzyl)(methyl)amino)-4-(trifluoromethyl)phenyl)-3-phenylprop-2-en-1-one (9k)

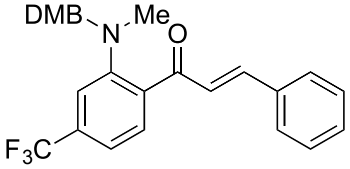

Prepared following general procedure B from 2-((3,4-dimethoxybenzyl)(methyl)amino)-4-trifluoromethylbenzaldehyde **8j** (177 mg, 0.50 mmol), phenylacetylene (85 µL, 0.75 mmol), [Rh(nbd)$_2$]BF$_4$ (9.4 mg, 25 µmol), dcpm (10.2 mg, 25 µmol), MeCN (2% in DCE, 125 µL, 50 µmol) in acetone (4 mL). Flash chromatography (10% Et$_2$O in petrol) afforded the title compound as a yellow oil (225 mg; 98%). ^{1}H NMR (400 MHz, CDCl$_3$): δ = 7.57 (d, *J* = 16 Hz, 1H, C*H*Ph), 7.51 (dd, *J* = 8 Hz, *J* = 0.5 Hz, 1H, *Ar-H*), 7.48–7.44 (m, 2H, *Ar-H*), 7.34–7.29 (m, 3H, *Ar-H*), 7.20 (d, *J* = 16 Hz, 1H, COC*H*) and 7.22–7.16 (m, 2H, *Ar-H*) overlapping, 6.65–6.57 (m, 3H, *Ar-H*), 4.12 (s, *2*H, N*CH$_2$*), 3.73 (s, 3H, O*CH$_3$*), 3.56 (s, 3H, O*CH$_3$*), 2.65 (s, 3H, N*CH$_3$*). ^{13}C NMR (100 MHz, CDCl$_3$): δ = 194.5, 151.5, 148.9, 148.3, 144.2, 135.3, 134.6, 133.1 (q, $^2J_{CF_3}$ = 32 Hz), 130.6, 129.3, 128.9, 128.4, 125.6, 123.7 (q, $^1J_{CF_3}$ = 274 Hz), 120.5, 117.2 (q, $^3J_{CF_3}$ = 4 Hz), 115.3 (q, $^3J_{CF_3}$ = 4 Hz), 111.0, 110.8, 60.4, 55.7, 55.5, 40.9. IR: ν_{max} (cm^{-1}) 2950, 1655, 1592, 1509, 1455, 1415, 1261, 1239, 1163, 828, 765. LRMS (ESI): *m/z* 456 (100%, [M+H]$^+$), 478 (15%, [M+Na]$^+$). HRMS (ESI): Found 478.1584 (M+Na$^+$), calculated for $C_{26}H_{24}F_3NNaO_3$ is 478.1600.

(*E*)-1-(4-Bromo-2-((3,4-dimethoxybenzyl)(methyl)amino)phenyl)-3-phenylprop-2-en-1-one (9l)

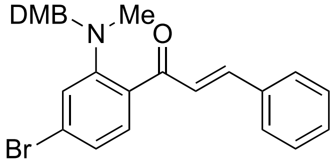

Prepared following general procedure B from 4-bromo-2-((3,4-dimethoxybenzyl)(methyl)amino)benzaldehyde **8k** (182 mg, 0.50 mmol), phenylacetylene (85 µL, 0.75 mmol), $[Rh(nbd)_2]BF_4$ (9.4 mg, 25 µmol), dcpm (10.2 mg, 25 µmol), MeCN (2% in DCE, 125 µL, 50 µmol) in acetone (4 mL). Flash chromatography (10% Et_2O in petrol) afforded the title compound as a yellow oil (171 mg; 73%). ^1H NMR (400 MHz, $CDCl_3$): δ = 7.67 (d, *J* = 16 Hz, 1H, C*H*Ph), 7.57–7.52 (m, 2H, *Ar-H*), 7.42–7.38 (m, 4H, *Ar-H*), 7.33 (d, *J* = 16 Hz, 1H, COC*H*), 7.19 (d, *J* = 1.5 Hz, 1H, *Ar-H*), 7.15 (dd, *J* = 8 Hz, *J* = 2 Hz, 1H, *Ar-H*), 6.70 (s, 2H, *Ar-H*), 6.63 (s, 1H, *Ar-H*), 4.16 (s, *2*H, N*CH₂*), 3.82 (s, 3H, O*CH₃*), 3.66 (s, 3H, O*CH₃*), 2.69 (s, 3H, N*CH₃*). ^{13}C NMR (100 MHz, $CDCl_3$): δ = 194.1, 152.6, 148.9, 148.3, 143.5, 134.8, 131.7, 131.1, 130.4, 129.3, 128.9, 128.3, 126.1, 125.9, 123.8, 121.9, 120.6, 111.2, 110.8, 60.6, 55.8, 55.6, 40.9. IR: ν_{max} (cm^{-1}) 2932, 1642, 1607, 1557, 1510, 1449, 1417, 1261, 1237, 1185, 1139, 1029, 856, 771, 735, 694. LRMS (ESI): *m/z* 466 (100%, [[^{79}Br] M+H]$^+$), 468 (100%, [[^{81}Br] M+H]$^+$). HRMS (ESI): Found 488.0829 ([^{79}Br] M+Na$^+$), 490.0815 ([^{81}Br] M+Na$^+$), calculated for $C_{25}H_{24}{}^{79}BrNNaO_3$ is 488.0832, calculated for $C_{25}H_{24}{}^{81}BrNNaO_3$ is 490.0813.

***(E)*-1-(3-((3,4-Dimethoxybenzyl)(methyl)amino)thiophen-2-yl)-3-phenylprop-2-en-1-one (9m)**

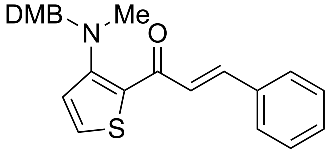

Prepared following general procedure B from 3-((3,4-dimethoxybenzyl)(methyl)amino)thiophene-2-carbaldehyde **8l** (145 mg, 0.50 mmol), phenylacetylene (85 µL, 0.75 mmol), $[Rh(nbd)_2]BF_4$ (9.4 mg, 25 µmol), dcpm (10.2 mg, 25 µmol), MeCN (2% in DCE, 125 µL, 50 µmol) in acetone (4 mL). Flash chromatography (10% Et_2O in petrol) afforded the title compound as a yellow oil (187 mg; 95%). ^1H NMR (400 MHz, $CDCl_3$): δ = 7.79 (d, *J* = 15.5 Hz, 1H, C*H*Ph), 7.61 (d, *J* = 15.5 Hz, 1H, COC*H*), 7.58–7.56 (m, 2H, *Ar-H*), 7.47 (d, *J* = 5.5 Hz, 1H, SC*H*), 7.42–7.35 (m, 3H, *Ar-H*), 6.88 (d, *J* = 5.5 Hz, 1H, SCHC*H*), 6.81 (s, 2H, *Ar-H*), 6.79 (s, 1H, *Ar-H*), 4.43 (s, 2H, NC*H₂*), 3.86 (s, 3H, OC*H₃*), 3.78 (s, 3H, OC*H₃*), 2.89 (s, 3H, NC*H₃*). ^{13}C NMR (100 MHz, $CDCl_3$): δ = 181.0, 156.7, 148.9, 148.2, 141.7, 135.2, 131.7, 129.9, 128.8, 128.1, 124.8, 122.7, 122.1, 120.3, 111.0, 110.8, 60.5, 55.8, 55.7, 42.5. IR: ν_{max} (cm^{-1}) 2972, 2850, 1683, 1594, 1484, 1466, 1277, 1226, 1190, 1169, 1091, 727, 684. LRMS (ESI): *m/z*: 394 (70%, [M+H]$^+$), 416 (100%, [M+Na]$^+$). HRMS (ESI): Found 416.1284 (M+Na$^+$), calculated for $C_{23}H_{23}NNaO_3S$ is 416.1291.

***(E)*-1-(2-((3,4-Dimethoxybenzyl)(methyl)amino)phenyl)-non-2-en-1-one (9n)**

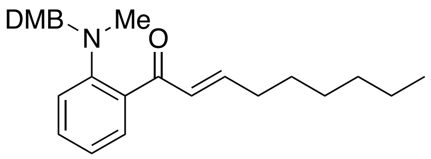

Prepared following general procedure B from 2-((3,4-dimethoxybenzyl)(methyl)amino)benzaldehyde **8d** (143 mg, 0.50 mmol), 1-octyne (110 µL, 0.75 mmol), $[Rh(nbd)_2]BF_4$ (9.4 mg, 25 µmol), dcpm (10.2 mg, 25 µmol), MeCN (2% in DCE, 125 µL, 50 µmol) in acetone (4 mL). Flash chromatography (10% Et_2O in petrol) afforded the title compound as a yellow oil (194 mg; 98%).

^{1}H NMR (400 MHz, $CDCl_3$): δ = 7.42 (dd, *J* = 7.5 Hz, *J* = 1.5 Hz, 1H, *Ar-H*), 7.34 (td, *J* = 7.5 Hz, *J* = 1.5 Hz, 1H, *Ar-H*), 7.00–6.95 (m, 2H, *Ar-H*), 6.90 (dt, *J* = 15.5 Hz, *J* = 7 Hz, 1H, C*H*$(CH_2)_5CH_3$), 6.78–6.67 (m, 4H, *Ar-H* and COC*H* overlapping), 4.12 (s, 2H, NC*H₂*), 3.84 (s, 3H, OC*H₃*), 3.79 (s, 3H, OC*H₃*), 2.65 (s, 3H, NC*H₃*), 2.24 (q, *J* = 7 Hz, 2H, $CHCH_2(CH_2)_4CH_3$), 1.46 (qu, *J* = 7 Hz, 2H, $CH_2(CH_2)_3CH_3$), 1.35–1.20 (m, 6H, $CH_2(CH_2)_3CH_3$), 0.87 (t, *J* = 7 Hz, 3H, $(CH_2)_5CH_3$). ^{13}C NMR (100 MHz, $CDCl_3$): δ = 195.9, 151.2, 148.7(1), 148.6(5), 148.0, 132.6, 131.2, 130.1, 130.0, 129.9, 120.6, 120.4, 118.8, 111.2, 110.6, 60.6, 55.7, 55.6, 40.6, 32.5, 31.5, 28.8, 28.1, 22.4, 14.0. IR: ν_{max} (cm^{-1}) 2929, 2855, 1666, 1615, 1593, 1515, 1421, 1261, 1029, 763. LRMS (ESI): *m/z*: 396 (70%, $[M+H]^+$), 418 (20%, $[M+Na]^+$), 813 (100%, $[2M+Na]^+$). HRMS (ESI): Found 396.2520 ($M+H^+$), calculated for $C_{25}H_{34}NO_3$ is 396.2533.

(E)-1-(2-((3,4-Dimethoxybenzyl)(methyl)amino)phenyl)-6-methylhept-2-en-1-one (9o)

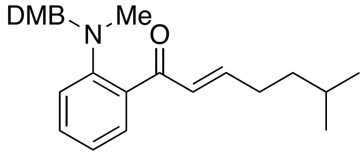

Prepared following general procedure B from 2-((3,4-dimethoxybenzyl)(methyl)amino)benzaldehyde **8d** (143 mg, 0.50 mmol), 5-methyl-1-hexyne (100 µL, 0.75 mmol), $[Rh(nbd)_2]BF_4$ (9.4 mg, 25 µmol), dcpm (10.2 mg, 25 µmol), MeCN (2% in DCE, 125 µL, 50 µmol) in acetone (4 mL). Flash chromatography (10% Et_2O in petrol) afforded the title compound as a yellow oil (170 mg; 89%). ^{1}H NMR (400 MHz, $CDCl_3$): δ = 7.41 (d, *J* = 7.5 Hz, 1H, *Ar-H*), 7.33 (dd, *J* = 8 Hz, *J* = 7.5 Hz, 1H, *Ar-H*), 6.99–6.94 (m, 2H, *Ar-H*), 6.89 (dt, *J* = 15.5 Hz, *J* = 7.5 Hz, 1H, COCHC*H*), 6.77–6.67 (m, 4H, *Ar-H* and COC*H* overlapping), 4.11 (s, 2H, NC*H₂*), 3.83 (s, 3H, OC*H₃*), 3.79 (s, 3H, OC*H₃*), 2.64 (s, 3H, NC*H₃*), 2.24 (q, *J* = 7.5 Hz, 2H, $(CH)_2CH_2CH_2$), 1.57 (m, 1H, C*H*$(CH_3)_2$) 1.33 (q, *J* = 7.5 Hz, 2H, $CH_2CH(CH_3)_2$), 0.88 (d, *J* = 6.5 Hz, 6H, $CH(CH_3)_2$). ^{13}C NMR (100 MHz, $CDCl_3$): δ = 195.9, 151.1, 148.7, 148.6, 148.0, 132.5, 131.2, 130.0, 129.8 (2C), 120.6, 120.3, 118.7, 111.2, 110.5, 60.6, 55.6(5), 55.5(9), 40.6, 37.1, 30.4, 27.4, 22.2. IR: ν_{max} (cm^{-1}) 2935, 2856, 1666, 1615, 1597, 1514, 1421, 1259, 1030, 765. LRMS (ESI): *m/z*: 382 (100%, $[M+H]^+$), 404 (65%, $[M+Na]^+$). HRMS (ESI): Found 382.2371 ($M+H^+$), calculated for $C_{24}H_{32}NO_3$ is 382.2377.

(*E*)-1-(2-((3,4-dimethoxybenzyl)(methyl)amino)phenyl)-4,4-dimethylpent-2-en-1-one (9p)

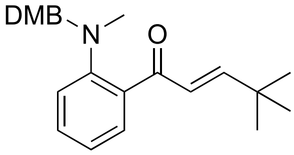

Prepared following general procedure B from 2-((3,4-dimethoxybenzyl)(methyl)amino)benzaldehyde **8d** (143 mg, 0.50 mmol), 3,3-dimethyl-1-butyne (92 µL, 0.75 mmol), $[Rh(nbd)_2]BF_4$ (9.4 mg, 25 µmol), dcpm (10.2 mg, 25 µmol), MeCN (2% in DCE, 125 µL, 50 µmol) in acetone (4 mL). Flash chromatography (10% Et_2O in petrol) afforded the title compound as a yellow oil (170 mg; 93%). ^1H NMR (400 MHz, $CDCl_3$): δ = 7.45 (dd, *J* = 8 Hz, *J* = 1.5 Hz, 1H, *Ar-H*), 7.35 (ddd, *J* = 8 Hz, *J* = 7.5 Hz, *J* = 1.5 Hz, 1H, *Ar-H*), 7.00–6.96 (m, 2H, *Ar-H*), 6.91 (d, *J* = 16 Hz, 1H, COC*H*), 6.77 (d, *J* = 8 Hz, 1H, *Ar-H*), 6.73 (dd, *J* = 8 Hz, *J* = 2 Hz, 1H, *Ar-H*), 6.63 (d, *J* = 16 Hz, 1H, COCHC*H*) and 6.62 (d, *J* = 2 Hz, 1H, *Ar-H*) overlapping, 4.11 (s, 2H, NCH_2), 3.85 (s, 3H, OCH_3), 3.78 (s, 3H, OCH_3) 2.67 (s, 3H, NCH_3), 1.10 (s, 9H, $C(CH_3)_3$). ^{13}C NMR (100 MHz, $CDCl_3$): δ = 196.4, 158.0, 151.2, 148.7, 148.1, 132.6, 131.3, 130.1, 129.9, 125.1, 120.6, 120.5, 118.9, 111.4, 110.7, 60.7, 55.8, 55.7, 40.5, 33.8, 28.8. IR: ν_{max} (cm^{-1}) 2957, 1664, 1612, 1593, 1514, 1485, 1449, 1421, 1364, 1312, 1260, 1238, 1139, 1027, 983, 923, 844, 811, 761, 695, 653. LRMS (ESI): *m/z* 368 (100%, $[M+H]^+$). HRMS (ESI): Found 390.2032 ($M+Na^+$), calculated for $C_{23}H_{29}NNaO_3$ is 390.2040.

(*E*)-6-chloro-1-(2-((3,4-dimethoxybenzyl)(methyl)amino)phenyl)hex-2-en-1-one (9q)

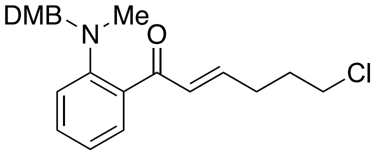

Prepared following general procedure B from 2-((3,4-dimethoxybenzyl)(methyl)amino)benzaldehyde **X** (143 mg, 0.50 mmol), 5-chloro-1-pentyne (80 µL, 0.75 mmol), $[Rh(nbd)_2]BF_4$ (9.4 mg, 25 µmol), dcpm (10.2 mg, 25 µmol), MeCN (2% in DCE, 125 µL, 50 µmol) in acetone (4 mL). Flash chromatography (10% Et_2O in petrol) afforded the title compound as a yellow oil (147 mg, 75%). ^1H NMR (400 MHz, $CDCl_3$): δ = 7.45 (dd, *J* = 8 Hz, *J* = 1.5 Hz, 1H, *Ar-H*), 7.33 (ddd, *J* = 8 Hz, *J* = 7.5 Hz, *J* = 1.5 Hz, 1H, *Ar-H*), 7.02–6.93 (m, 2H, *Ar-H*), 6.88–6.63 (m, 6H, 4 × *Ar-H* + COC*H* + COCHC*H* overlapping), 4.12 (s, 2H, NCH_2), 3.85 (s, 3H, OCH_3), 3.77 (s, 3H, OCH_3), 3.54 (t, *J* = 6.5 Hz, 2H, CH_2Cl), 2.66 (s, 3H, NCH_3), 2.42 (q, *J* = 6.5 Hz, 2H, $CH_2(CH_2)_2Cl$), 1.93 (qu, *J* = 6.5 Hz, 2H, CH_2CH_2Cl). ^{13}C NMR (100 MHz, $CDCl_3$): δ = 195.2, 151.3, 148.7, 148.1, 145.4, 132.4, 131.5, 130.8, 130.1, 129.8, 120.8, 120.5, 118.9, 111.4, 110.7, 60.8, 55.8, 55.7, 44.0, 40.7, 30.8, 29.4. IR: ν_{max} (cm^{-1}) 2936, 1666, 1593, 1514, 1448, 1421, 1260, 1237, 1138, 1027, 867, 811, 762, 654. LRMS (ESI): *m/z*: 388 (5%, $[M+H]^+$), 410 (100%, $[M+Na]^+$). HRMS (ESI): Found 410.1487 ($M+Na^+$), calculated for $C_{22}H_{26}ClNNaO_3$ is 410.1493.

(*E*)-7-(2-((3,4-dimethoxybenzyl)(methyl)amino)phenyl)-7-oxohept-5-enenitrile (9r)

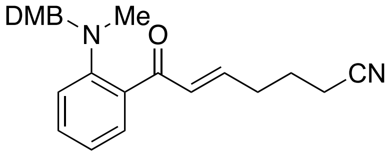

Prepared following general procedure B from 2-((3,4-dimethoxybenzyl)(methyl)amino)benzaldehyde **X** (143 mg, 0.50 mmol), 5-hexynenitrile (79 µL, 0.75 mmol), $[Rh(nbd)_2]BF_4$ (9.4 mg, 25 µmol), dcpm (10.2 mg, 25 µmol), MeCN (2% in DCE, 125 µL, 50 µmol) in acetone (4 mL). Flash chromatography (10% Et_2O in petrol) afforded the title compound as a yellow oil (147 mg, 75%). ^1H NMR (400 MHz, $CDCl_3$): δ = 7.47 (dd, *J* = 8 Hz, *J* = 1.5 Hz, 1H, *Ar-H*), 7.37 (ddd, *J* = 8 Hz, *J* = 7.5 Hz, *J* = 1.5 Hz, 1H, *Ar-H*), 7.02–6.97 (m, 2H, *Ar-H*), 6.85–6.77 (m, 3H, *Ar-H* + COC*H* + COCHC*H* overlapping), 6.71 (dd, *J* = 8 Hz, *J* = 2 Hz, 1H, *Ar-H*), 6.59 (d, *J* = 2 Hz, 1H, *Ar-H*), 4.11 (s, 2H, NCH_2), 3.85 (s, 3H, OCH_3), 3.77 (s, 3H, OCH_3), 2.66 (s, 3H, NCH_3), 2.45–2.39 (m, 2H, C$H_2$$(CH_2)_2$CN), 2.37 (t, *J* = 7 Hz, 2H, CH_2CN), 1.83 (qu, *J* = 7 Hz, 2H, C$H_2$$CH_2$CN). ^{13}C NMR (100 MHz, $CDCl_3$): δ = 194.9, 151.5, 148.7, 148.2, 143.8, 132.3, 131.8, 131.3, 130.2, 129.7, 120.9, 120.6, 119.4, 119.0, 111.5, 110.7, 61.0, 55.8(4), 55.7(9), 40.7, 30.9, 24.0, 16.6. IR: ν_{max} (cm^{-1}) 2937, 1665, 1615, 1593, 1515, 1486, 1449, 1421, 1260, 1237, 1139, 1027, 813, 764. LRMS (ESI): *m/z*: 379 (100%, $[M+H]^+$), 401 (20%, $[M+Na]^+$). HRMS (ESI): Found 401.1823 ($M+Na^+$), calculated for $C_{23}H_{26}N_2NaO_3$ is 401.1836.

(E)-1-(2-((3,4-Dimethoxybenzyl)(methyl)amino)phenyl)-3-(trimethylsilyl)prop-2-en-1-one (9s)

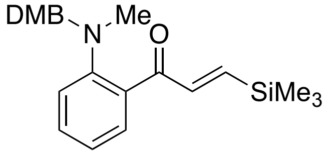

Prepared following general procedure B from 2-((3,4-dimethoxybenzyl)(methyl)amino)benzaldehyde **8d** (143 mg, 0.50 mmol), trimethylsilylacetylene (110 µL, 0.75 mmol), $[Rh(nbd)_2]BF_4$ (9.4 mg, 25 µmol), dcpm (10.2 mg, 25 µmol), MeCN (2% in DCE, 125 µL, 50 µmol) in acetone (4 mL). Flash chromatography (10% Et_2O in petrol) afforded the title compound as a yellow oil (159 mg, 83%).. ^1H NMR (400 MHz, $CDCl_3$): δ = 7.48 (dd, *J* = 7.5 Hz, *J* = 1.5 Hz, 1H, *Ar-H*), 7.34 (dd, *J* = 8 Hz, *J* = 1.5 Hz, 1H, *Ar-H*), 7.16 (d, *J* = 15.5 Hz, 1H, C*H*$SiMe_3$), 7.09 (d, *J* = 15.5 Hz, 1H, COC*H*), 7.02–6.98 (m, 2H, *Ar-H*), 6.78–6.70 (m, 2H, *Ar-H*), 6.59 (s, 1H, *Ar-H*), 4.09 (s, 2H, NCH_2), 3.85 (s, 3H, OCH_3), 3.77 (s, 3H, OCH_3), 2.67 (s, 3H, NCH_3), 0.16 (s, 9H, Si$(CH_3)_3$). ^{13}C NMR (100 MHz, $CDCl_3$): δ = 195.0, 151.6, 148.7, 148.1, 146.6, 141.9, 132.1, 131.6, 130.3, 129.7, 120.8, 120.4, 119.1, 111.4, 110.7, 61.0, 55.8, 55.7, 40.3, -1.7. IR: ν_{max} (cm^{-1}) 2956, 1656, 1594, 1515, 1485, 1449, 1421, 1235, 1139, 1029, 1008, 848, 762. LRMS (ESI): *m/z*: 384 (100%, $[M+H]^+$), 406 (45%, $[M+Na]^+$). HRMS (ESI): Found 384.1976 ($M+H^+$), calculated for $C_{22}H_{30}NO_3Si$ is 384.1989.

(E)-1-(2-((3,4-Dimethoxybenzyl)(methyl)amino)phenyl)-3-ferrocenylprop-2-en-1-one (9t)

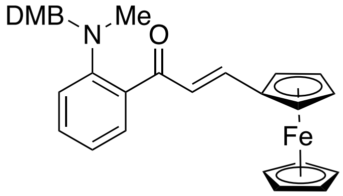

Prepared following general procedure B from 2-((3,4-dimethoxybenzyl)(methyl)amino)benzaldehyde **8d** (143 mg, 0.5 mmol), ethynylferrocene (158 mg, 0.75 mmol), [Rh(nbd)$_2$]BF_4 (9.4 mg, 25 μmol), dcpm (10.2 mg, 25 μmol), MeCN (2% in DCE, 125 μL, 50 μmol) in acetone (4 mL). Flash chromatography (10% Et_2O in petrol) afforded the title compound as a red oil (244 mg, 98%). ^{1}H NMR (400 MHz, $CDCl_3$): δ = 7.57 (d, *J* = 15.5 Hz, 1H, C*H*C_5H_5), 7.51 (dd, 1H, *J* = 8 Hz, *J* = 1.5 Hz, *Ar-H*), 7.39 (ddd, *J* = 8 Hz, *J* = 7.5 Hz, *J* = 1.5 Hz, 1H, *Ar-H*), 7.04 (m, 2H, *Ar-H*), 6.97 (d, *J* = 16 Hz, 1H, COC*H*), 6.76 (s, 2H, *Ar-H*), 6.70 (s, 1H, *Ar-H*), 4.51 (t, *J* = 2 Hz, 2H, *Fc-H*), 4.45 (t, *J* = 2 Hz, 2H, *Fc-H*), 4.17 (s, 2H, NCH_2), 4.12 (s, 5H, *Fc-H*), 3.85 (s, 3H, OCH_3), 3.73 (s, 3H, OCH_3), 2.70 (s, 3H, NCH_3). ^{13}C NMR (100 MHz, $CDCl_3$): δ = 195.1, 151.3, 148.8, 148.1, 145.2, 133.1, 131.2, 130.1, 130.0, 123.9, 120.8, 120.6, 118.7, 111.3, 110.7, 79.2, 71.1, 69.6, 68.8, 60.7, 55.8, 55.7, 40.7. IR: ν_{max} (cm^{-1}) 2936, 2362, 1653, 1595, 1515, 1421, 1359, 1308, 1140, 1105, 1026, 818, 763. LRMS (ESI): *m/z*: 496 (100%, [M+H]$^+$). HRMS (ESI): Found 496.1554 (M+H$^+$), calculated for $C_{29}H_{30}FeNO_3$ is 496.1570.

(*E*)-1-(2-((3,4-Dimethoxybenzyl)(methyl)amino)phenyl)-3-(4-fluorophenyl)prop-2-en-1-one (9u)

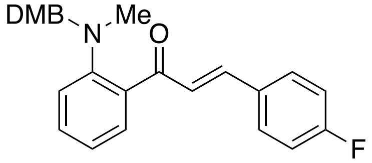

Prepared following general procedure B from 2-((3,4-dimethoxybenzyl)(methyl)amino)benzaldehyde **8d** (143 mg, 0.5 mmol), 1-fluoro-4-ethynylbenzene (90 mg, 0.75 mmol), [Rh(nbd)$_2$]BF_4 (9.4 mg, 25 μmol), dcpm (10.2 mg, 25 μmol), MeCN (2% in DCE, 125 μL, 50 μmol) in acetone (4 mL). Flash chromatography (10% Et_2O in petrol) afforded the title compound as a red oil (183 mg, 90%). ^{1}H NMR (400MHz, $CDCl_3$): δ = 7.65 (d, *J* = 16 Hz, 1H, COCHC*H*), 7.57–7.52 (m, 3H, *Ar-H*), 7.42 (ddd, *J* = 8 Hz, *J* = 7.5 Hz, *J* = 1.5 Hz, 1H, *Ar-H*), 7.34 (d, *J* = 16 Hz, 1H, COC*H*), 7.11–7.04 (m, 4H, *Ar-H*), 6.74–6.65 (m, 4H, *Ar-H*), 4.16 (s, 2H, NCH_2), 3.83 (s, 3H, OCH_3), 3.67 (s, 3H, OCH_3), 2.70 (s, 3H, NCH_3). ^{13}C NMR (400MHz, $CDCl_3$): δ = 195.0, 165.1, 148.8, 148.2, 141.3, 133.0, 131.8, 131.3 (d, ^4J_F = 3 Hz), 130.2, 130.1 (d, ^3J_F = 8.5 Hz), 129.8, 126.2, 121.2, 120.6, 119.0, 116.0 (d, ^2J_F = 21 Hz), 111.4, 110.7, 61.0, 55.8, 55.6, 41.0. IR: ν_{max} (cm^{-1}) 2936, 1659, 1597, 1509, 1449, 1417, 1322, 1260, 1232, 1157, 1025, 832, 763. LRMS (ESI): *m/z*: 406 (90%, [M+H]$^+$), 428 (60%, [M+Na]$^+$), 833 (100%, [2M+Na]$^+$). HRMS (ESI): Found 406.1814 (M+H$^+$), calculated for $C_{25}H_{25}FNO_3$ is 406.1813.

(*E*)-1-(2-((3,4-dimethoxybenzyl)(methyl)amino)phenyl)-2-methyl-3-phenylprop-2-en-1-one (9v)

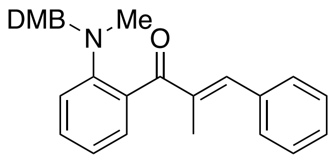

Prepared following general procedure B from 2-((3,4-dimethoxybenzyl)(methyl)amino)benzaldehyde **X** (143 mg, 0.50 mmol), 1-phenyl-1-propyne (94 µL, 0.75 mmol), $[Rh(nbd)_2]BF_4$ (9.4 mg, 25 µmol), dcpm (10.2 mg, 25 µmol), MeCN (2% in DCE, 125 µL, 50 µmol) in acetone (4 mL). Flash chromatography (10% Et_2O in petrol) afforded the title compound as a yellow oil (162 mg, 81%). ^1H NMR (400 MHz, $CDCl_3$): δ = 7.41–7.31 (m, 6H, *Ar-H*), 7.29 (dd, *J* = 7.5 Hz, *J* = 1.5 Hz, 1H, *Ar-H*), 7.24 (d, *J* = 1 Hz, 1H, C*H*Ph), 7.09 (d, *J* = 8 Hz, 1H, *Ar-H*), 7.03 (t, *J* = 7.5 Hz, 1H, *Ar-H*), 6.76–6.69 (m, 3H, *Ar-H*), 4.11 (s, 2H, NCH_2), 3.82 (s, 3H, OCH_3), 3.66 (s, 3H, OCH_3), 2.63 (s, 3H, NCH_3), 2.22 (d, *J* = 1 Hz, 3H, COC(CH_3)). ^{13}C NMR (100 MHz, $CDCl_3$): δ = 201.2, 151.0, 148.9, 148.0, 142.8, 137.4, 136.0, 132.9, 130.7, 130.5, 129.8, 129.5, 128.6, 128.4, 121.0, 120.2, 119.1, 111.0, 110.6, 60.3, 55.8, 55.6, 40.7, 13.4. IR: ν_{max} (cm^{-1}) 2935, 1646, 1593, 1513, 1447, 1421, 1359, 1260, 1139, 1028, 1011, 916, 811, 760, 696. LRMS (ESI): *m/z*: 402 (100%, $[M+H]^+$), 424 (5%, $[M+Na]^+$). HRMS (ESI): Found 424.1872 ($M+Na^+$), calculated for $C_{26}H_{27}NNaO_3$ is 424.1883.

(*E*)-1-(2-Aminophenyl)non-2-en-1-one (9w)

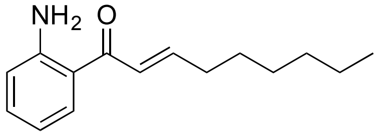

Prepared following general procedure B from 2-aminobenzaldehyde **8f** (61 mg, 0.50 mmol), 1-octyne (110 µL, 0.75 mmol), $[Rh(nbd)_2]BF_4$ (9.4 mg, 25 µmol), dcpm (10.2 mg, 25 µmol), MeCN (2% in DCE, 125 µL, 50 µmol) in acetone (4 mL). Flash chromatography (10% Et_2O in petrol) afforded the title compound as an orange solid (97 mg; 84%). ^1H NMR (400 MHz, $CDCl_3$): δ = 7.75 (dd, *J* = 8.5 Hz, *J* = 1.5 Hz, 1H, *Ar-H*), 7.25 (ddd, *J* = 8.5 Hz, *J* = 7 Hz, *J* = 1.5 Hz, 1H, *Ar-H*), 7.02–6.92 (m, 2H, C*H*$(CH_2)_5CH_3$ + *Ar-H*), 6.67–6.63 (m, 2H, COC*H* + *Ar-H*), 6.27 (br s, *2*H, –*NH$_2$*), 2.32–2.27 (m, 2H, CHC$H_2(CH_2)_4CH_3$), 1.51 (qu, *J* = 7 Hz, 2H, $CHCH_2CH_2(CH_2)_3CH_3$), 1.39–1.25 (m, 6H, $CHCH_2CH_2(CH_2)_3CH_3$), 0.90 (t, *J* = 7 Hz, 3H, $CH(CH_2)_5CH_3$). ^{13}C NMR (100 MHz, $CDCl_3$): δ = 192.2, 150.8, 147.6, 134.0, 131.1, 126.4, 118.6, 117.1, 115.6, 32.7, 31.6, 28.9, 28.2, 22.5, 14.0. IR: ν_{max} (cm^{-1}) 3457, 3336, 2926, 2856, 1651, 1614, 1578, 1544, 1483, 1448, 1293, 1220, 1160, 970, 908, 854, 746, 659. LRMS (ESI): *m/z* 232 (100%, $[M+H]^+$). HRMS (ESI): Found 254.1518 ($M+Na^+$), calculated for $C_{15}H_{21}NNaO$ is 254.1515.

(*E*)-1-(2-Aminophenyl)-3-cyclohexylprop-2-en-1-one (9x)

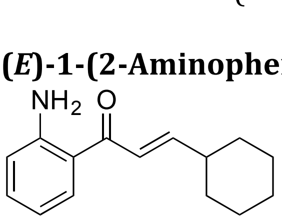

Prepared following general procedure B from 2-aminobenzaldehyde **8f** (61 mg, 0.50 mmol), cyclohexylacetylene (98 µL, 0.75 mmol), $[Rh(nbd)_2]BF_4$ (9.4 mg, 25 µmol), dcpm (10.2 mg, 25 µmol), MeCN (2% in DCE, 125 µL, 50 µmol) in acetone (4 mL). Flash chromatography (10% Et_2O in petrol) afforded the title compound

as a yellow oil (70 mg; 61%). ^{1}H NMR (400 MHz, $CDCl_3$): δ = 7.75 (dd, *J* = 8.5 Hz, *J* = 1.5 Hz, 1H, *Ar-H*), 7.26 (ddd, *J* = 8.5 Hz, *J* = 7 Hz, *J* = 1.5 Hz, 1H, *Ar-H*), 6.96–6.87 (m, 2H, C*H*Cy + *Ar-H*), 6.67–6.63 (m, 2H, COC*H* + *Ar-H*), 6.24 (br s, *2*H, –*NH$_2$*), 2.23 (m, 1H, *Cy-H*), 1.85–1.15 (m, 10H, *Cy-H*). ^{13}C NMR (100 MHz, $CDCl_3$): δ = 192.7, 152.6, 150.8, 134.0, 131.2, 124.0, 118.9, 117.2, 115.7, 40.9, 32.0, 26.0, 25.8. IR: ν_{max} (cm^{-1}) 3454, 3334, 2925, 2851, 1651, 1615, 1579, 1545, 1483, 1448, 1336, 1259, 1231, 1161, 1007, 964, 747, 662. LRMS (ESI): *m/z* 230 (100%, $[M+H]^+$), 252 (10%, $[M+Na]^+$). HRMS (ESI): Found 252.1362 ($M+Na^+$), calculated for $C_{15}H_{19}NNaO$ is 252.1359.

(*E*)-1-(2-Aminophenyl)-2-ethylpent-2-en-1-one (9y)

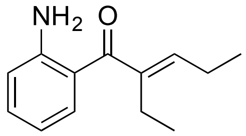

Prepared following general procedure B from 2-aminobenzaldehyde **8f** (61 mg, 0.50 mmol), 3-hexyne (85 μL, 0.75 mmol), $[Rh(nbd)_2]BF_4$ (9.4 mg, 25 μmol), dcpm (10.2 mg, 25 μmol), MeCN (2% in DCE, 125 μL, 50 μmol) in acetone (4 mL). Flash chromatography (10% Et_2O in petrol) afforded the title compound as a yellow oil (89 mg; 78%). ^{1}H NMR (400 MHz, $CDCl_3$): δ = 7.53 (dd, *J* = 8 Hz, *J* = 1.5 Hz, 1H, *Ar-H*), 7.23 (ddd, *J* = 8.5 Hz, *J* = 7 Hz, *J* = 1.5 Hz, 1H, *Ar-H*), 6.66 (dd, *J* = 8.5 Hz, *J* = 1 Hz, 1H, *Ar-H*), 6.61 (ddd, *J* = 8 Hz, *J* = 7 Hz, *J* = 1 Hz, 1H, *Ar-H*), 5.87 (t, *J* = 7.5 Hz, 1H, C*H*Et), 5.77 (br s, *2*H, –*NH$_2$*), 2.47 (q, *J* = 7.5 Hz, 2H, CC*H$_2$*Me), 2.25 (qu, *J* = 7.5 Hz, 2H, CHC*H$_2$*Me), 1.04 (t, *J* = 7.5 Hz, 6H, 2 × CH_2C*H$_3$*). ^{13}C NMR (100 MHz, $CDCl_3$): δ = 201.5, 150.1, 142.0, 141.1, 133.5, 133.3, 119.5, 116.7, 115.3, 21.5, 20.7, 13.6, 13.4. IR: ν_{max} (cm^{-1}) 3468, 3350, 2966, 1613, 1581, 1548, 1479, 1449, 1304, 1236, 1158, 1048, 984, 871, 813, 751, 643. LRMS (ESI): *m/z* 204 (100%, $[M+H]^+$), 226 (25%, $[M+Na]^+$). HRMS (ESI): Found 226.1194 ($M+Na^+$), calculated for $C_{13}H_{17}NNaO$ is 226.1202.

GENERAL PROCEDURE C: CYCLIZATION OF PRIMARY 2-AMINO ARYL ENONES

2-Phenyl-2,3-dihydroquinolin-4(1*H*)-one (10a)

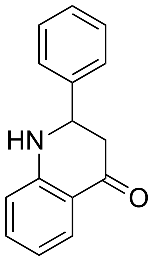

(*E*)-1-(2-aminophenyl)-3-phenylprop-2-en-1-one **9g** (67 mg, 0.30 mmol) was dissolved in dry MeCN (1 mL). Antimony(III) chloride (21 mg, 0.090 mmol) was added, and the reaction was heated to 55°C and left to stir for 6 hours. It was then cooled down to room temperature, and water (3 mL) was added. The water phase was extracted with DCM (3 × 5 mL) and the combined organic layers were washed with brine (3 × 5 mL) then dried over magnesium sulfate. The solvent was removed *in vacuo*, and flash chromatography (10% Et_2O in petrol) afforded the title compound as a yellow solid (62 mg, 92%). ^{1}H NMR (400MHz, $CDCl_3$): δ = 7.88 (dd, *J* = 8 Hz, *J* = 1.5 Hz, 1H, *Ar-H*), 7.48–7.32 (m, 6H, *Ar-H*), 6.80 (ddd, *J* = 8 Hz, *J* = 7 Hz, *J* = 1 Hz, 1H, *Ar-H*), 6.71 (app d, *J* = 8 Hz, 1H, *Ar-H*), 4.76 (dd, *J* = 13.5 Hz, *J* = 4 Hz, 1H, C*H*Ph), 4.51 (br s, 1H, –N*H*), 2.90 (dd, *J* = 16 Hz, *J* = 13.5 Hz, 1H, COC*H*H), 2.64 (ddd, *J* = 16 Hz, *J* = 4 Hz, *J* = 1 Hz, 1H, COCH*H*). ^{13}C NMR (400MHz, $CDCl_3$): δ = 193.2, 151.5, 141.0, 135.4, 129.0, 128.5, 127.6, 126.6, 119.1, 118.5, 115.9, 58.5, 46.5. LRMS (ESI): *m/z* 224 (100%, $[M+H]^+$), 246 (70%, $[M+Na]^+$), 469 (75%, $[2M+Na]^+$). M.p. 148–149 °C. Data consistent with previously reported values.[11]

2-Hexyl-2,3-dihydroquinolin-4(1*H*)-one (10b)

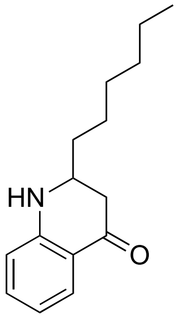

Prepared following general procedure C from (*E*)-1-(2-aminophenyl)non-2-en-1-one **9w** (69 mg, 0.30 mmol) and antimony(III) chloride (21 mg, 0.090 mmol) in MeCN (1 mL). Flash chromatography (20% Et_2O in petrol) afforded the title compound an off-white solid (53 mg, 77%).

^{1}H NMR (400MHz, $CDCl_3$): δ = 7.82 (dd, *J* = 8 Hz, *J* = 1.5 Hz, 1H, *Ar-H*), 7.30 (ddd, *J* = 8 Hz, *J* = 7 Hz, *J* = 1.5 Hz, 1H, *Ar-H*), 6.73 (ddd, *J* = 8 Hz, *J* = 7 Hz, *J* = 1 Hz, 1H, *Ar-H*), 6.67 (dd, *J* = 8 Hz, *J* = 0.5 Hz, 1H, *Ar-H*), 4.31 (br s, 1H, –N*H*), 3.63 (dtd, *J* = 12.5 Hz, *J* = 6.5 Hz, *J* = 3.5 Hz, 1H, C*H*$(CH_2)_5CH_3$), 2.68 (dd, *J* = 16 Hz, *J* = 3.5 Hz, 1H, COC*H*H), 2.48 (dd, *J* = 16 Hz, *J* = 12.5 Hz, 1H, COCH*H*), 1.69–1.57 (m, 2H, CH$\mathit{H_2}(CH_2)_4CH_3$), 1.43–1.26 (m, 8H, $CHCH_2\mathit{(CH_2)_4}CH_3$), 0.90 (t, *J* = 7 Hz, 3H, $CH(CH_2)_5\mathit{CH_3}$). ^{13}C NMR (400MHz, $CDCl_3$): δ = 194.1, 151.4, 135.1, 127.5, 119.1, 117.9, 115.7, 53.3, 44.0, 35.2, 31.7, 29.1, 25.3, 22.6, 14.0. IR: ν_{max} (cm^{-1}) 3457,

3336, 2926, 2856, 1651, 1614, 1578, 1544, 1483, 1448, 1293, 1220, 1160, 970, 908, 854, 746, 659. LRMS (ESI): *m/z* 232 (100%, $[M+H]^+$), 254 (35%, $[M+Na]^+$), 485 (50%, $[2M+Na]^+$). HRMS (ESI): Found 254.1518 ($M+Na^+$), calculated for $C_{15}H_{21}NNaO$ is 254.1515.

2-Cyclohexyl-2,3-dihydroquinolin-4(1*H*)-one (10c)

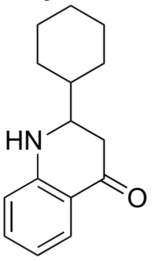

Prepared following general procedure C from (*E*)-1-(2-aminophenyl)-3-cyclohexylprop-2-en-1-one **9x** (69 mg, 0.30 mmol) and antimony(III) chloride (21 mg, 0.090 mmol) in MeCN (1 mL). Flash chromatography (20% Et_2O in petrol) afforded the title compound as an off-white solid (49 mg, 71%). ^{1}H NMR (400 MHz, $CDCl_3$): δ = 7.80 (dd, *J* = 8 Hz, *J* = 1.5 Hz, 1H, *Ar-H*), 7.29 (ddd, *J* = 8.5 Hz, *J* = 7 Hz, *J* = 1.5 Hz, 1H, *Ar-H*), 6.71 (ddd, *J* = 8 Hz, *J* = 7 Hz, *J* = 1 Hz, 1H, *Ar-H*), 6.66 (app d, *J* = 8.5 Hz, 1H, *Ar-H*), 4.34 (br s, 1H, N*H*), 3.42 (dt, *J* = 12 Hz, *J* = 4.5 Hz, 1H, C*H*Cy), 2.64 (ddd, *J* = 16 Hz, *J* = 4 Hz, *J* = 1.5 Hz, 1H, COC*H*H), 2.55 (dd, *J* = 16 Hz, *J* = 12 Hz, 1H, COCH*H*), 1.83–1.70 (m, 4H, *Cy-H*), 1.53 (m, 1H, *Cy-H*), 1.33–0.99 (m, 6H, *Cy-H*). ^{13}C NMR (100 MHz, $CDCl_3$): δ = 194.5, 151.6, 135.1, 127.4, 119.0, 117.7, 115.7, 58.1, 41.5, 41.0, 28.9, 28.6, 26.3, 26.1. IR: ν_{max} (cm^{-1}) 3350, 2925, 2852, 1655, 1611, 1510, 1482, 1447, 1341, 1255, 1212, 1157, 907, 759, 732. LRMS (ESI): *m/z* 230 (100%, $[M+H]^+$), 252 (45%, $[M+Na]^+$), 481 (90%, $[2M+Na]^+$). HRMS (ESI): Found 252.1356 ($M+Na^+$), calculated for $C_{15}H_{19}NNaO$ is 252.1359. M.p. 115–117 °C. The NMR data does not exactly match that reported in the literature.[12] We propose an acid impurity as a possible explanation for the discrepancy.

2,3-Diethyl-2,3-dihydroquinolin-4(1*H*)-one (10d and 10d')

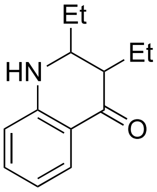

Prepared following general procedure C from (*E*)-1-(2-Aminophenyl)-2-ethylpent-2-en-1-one **9y** (61 mg, 0.30 mmol) and antimony(III) chloride (21 mg, 0.090 mmol) in MeCN (1 mL). Flash chromatography (20% Et_2O in petrol) afforded the title compound as as a 3:1 mixture of diastereomers (55 mg, 90%). Pure diastereomers were available for characterization purposes. **10d:** ^{1}H NMR (400 MHz, $CDCl_3$): δ = 7.79 (dd, *J* = 8 Hz, *J* = 1.5 Hz, 1H, *Ar-H*), 7.27 (ddd, *J* = 8.5 Hz, *J* = 7 Hz, *J* = 1.5 Hz, 1H, *Ar-H*), 6.68 (ddd, *J* = 8 Hz, *J* = 7 Hz, *J* = 1 Hz, 1H, *Ar-H*), 6.61 (app d, *J* = 8 Hz, 1H, *Ar-H*), 4.42 (br s, 1H, N*H*), 3.36 (td, *J* = 7 Hz, *J* = 5 Hz, 1H, NHC*H*Et), 2.31 (td, *J* = 7 Hz, *J* = 5 Hz, 1H, COC*H*Et), 1.83–1.50 (m, 4H, 2 × C*H*$_2$CH$_3$),

0.97 (t, J = 7.5 Hz, 3H, CH_2CH_3) and 0.96 (t, J = 7.5 Hz, 3H, CH_2CH_3) overlapping. ^{13}C NMR (100 MHz, $CDCl_3$): δ = 196.4, 149.1, 135.1, 127.6, 117.8, 117.3, 115.4, 56.9, 52.1, 25.5, 21.9, 11.4, 10.3. IR: ν_{max} (cm^{-1}) 3354, 2964, 1658, 1611, 1507, 1483, 1344, 1305, 1239, 1155, 998. LRMS (ESI): *m/z* 204 (100%, $[M+H]^+$), 226 (75%, $[M+Na]^+$). HRMS (ESI): Found 226.1199 ($M+Na^+$), calculated for $C_{13}H_{17}NNaO$ is 226.1202. M.p. 80–83 °C. **10d'**: ^{1}H NMR (400 MHz, $CDCl_3$): δ = 7.80 (dd, J = 8 Hz, J = 1.5 Hz, 1H, *Ar-H*), 7.28 (ddd, J = 8.5 Hz, J = 7 Hz, J = 1.5 Hz, 1H, *Ar-H*), 6.71 (ddd, J = 8 Hz, J = 7 Hz, J = 1 Hz, 1H, *Ar-H*), 6.63 (app d, J = 8 Hz, 1H, *Ar-H*), 4.33 (br s, 1H, N*H*), 3.52 (td, J = 7.5 Hz, J = 3.5 Hz, 1H, NHC*H*Et), 2.47 (td, J = 7.5 Hz, J = 3.5 Hz, 1H, COC*H*Et), 1.76–1.53 (m, 4H, 2 × CH_2CH_3), 0.98 (t, J = 7.5 Hz, 3H, CH_2CH_3), 0.94 (t, J = 7.5 Hz, 3H, CH_2CH_3). ^{13}C NMR (100 MHz, $CDCl_3$): δ = 196.9, 150.2, 134.8, 127.8, 118.5, 117.6, 115.3, 57.5, 51.2, 22.6, 16.6, 11.7, 10.4. IR: ν_{max} (cm^{-1}) 3354, 2964, 1658, 1611, 1507, 1483, 1344, 1305, 1239, 1155, 998, 752. LRMS (ESI): *m/z* 204 (100%, $[M+H]^+$), 226 (20%, $[M+Na]^+$). HRMS (ESI): Found 226.1199 ($M+Na^+$), calculated for $C_{13}H_{17}NNaO$ is 226.1202. The major diastereomer (**10d**) was assigned as the *trans* diastereomer on the basis of the coupling constant.

General Procedure D: Deprotection and cyclization of 2-amino aryl enones

1-Methyl-2-phenyl-2,3-dihydroquinolin-4(1H)-one (10e)

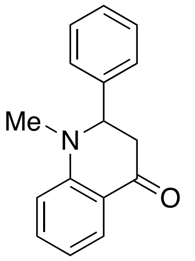

(E)-1-(2-((3,4-Dimethoxybenzyl)(methyl)amino)phenyl)-3-(phenyl)prop-2-en-1-one **9e** (120 mg, 0.30 mmol) was dissolved in dry DCM (3 mL), and trifluoromethanesulfonic acid (80 μL, 0.90 mmol) was added, and the solution was left to stir at room temperature for 1 hour, then quenched with a saturated aqueous solution of K_2CO_3 (3 mL). The water phase was extracted with DCM (3 × 3 mL) and the combined organic layers were washed with brine (3 mL) then dried over magnesium sulfate. The solvent was removed *in vacuoo*. The resulting oil was dissolved in dry MeCN (1 mL) and antimony(III) chloride (21 mg, 0.090 mmol) was added. The reaction was heated to 55°C and left to stir for 12 hours. It was then cooled down to room temperature, and water (3 mL) was added. The water phase was extracted with DCM (3 × 5 mL) and the combined organic layers were washed with brine (3 × 5 mL) then dried over magnesium sulfate. The solvent was removed *in vacuo*, and flash chromatography (10% Et_2O in petrol) afforded the title compound as a yellow oil (61 mg, 85%). ^{1}H NMR (400 MHz, $CDCl_3$): δ = 7.87 (dd, J = 8 Hz, J = 1.5 Hz, 1H, *Ar-H*), 7.46 (ddd, J = 8.5 Hz, J = 7 Hz, J = 1.5 Hz, 1H, *Ar-H*), 7.31–7.24 (m, 3H, *Ar-H*), 7.18–7.16 (m, 2H, *Ar-H*), 6.78 (d, J = 8.5 Hz, 1H, *Ar-H*), 6.76 (dd, J = 8.5 Hz, J = 8 Hz, 1H, *Ar-H*), 4.67 (t, J = 6.5 Hz, 1H, C*H*Ph), 3.16 (dd, J = 16.5 Hz, J = 6.5 Hz, 1H, COC*H*H), 2.94 (s, 3H, NCH_3) and 2.92 (dd, J = 16.5 Hz, J = 6.5 Hz, 1H, COCH*H*) overlapping. ^{13}C NMR (100 MHz, $CDCl_3$): δ = 192.4, 151.7, 139.9, 136.0, 128.9, 127.8, 127.6, 126.5, 119.7, 116.6,

112.8, 64.6, 45.4, 37.8. LRMS (ESI): *m/z*: 238 (70%, $[M+H]^+$), 260 (100%, $[M+Na]^+$). Data consistent with previously reported values.[13]

1-Methyl-2-hexyl-2,3-dihydroquinolin-4(1H)-one (10f)

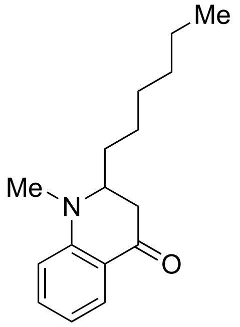

Prepared following general cyclisation procedure from (E)-1-(2-((3,4-dimethoxybenzyl)(methyl)amino)phenyl)-non-2-en-1-one **9n** (119 mg, 0.30 mmol), trifluoromethanesulfonic acid (80 µL, 0.90 mmol) in DCM (3 mL), then antimony(III) chloride (21 mg, 0.090 mmol) in MeCN (1 mL). Flash chromatography (10% Et_2O in petrol) afforded the title compound as a dark oil (51 mg; 69%).
^{1}H NMR (400 MHz, $CDCl_3$): δ = 7.85 (dd, *J* = 8 Hz, *J* = 1.5 Hz, 1H, *Ar-H*), 7.38 (ddd, *J* = 8.5 Hz, *J* = 8 Hz, *J* = 1.5 Hz, 1H, *Ar-H*), 6.68 (dd, *J* = 8.5 Hz, *J* = 8 Hz, 1H, *Ar-H*), 6.60 (d, *J* = 8.5 Hz, 1H, *Ar-H*), 3.51 (dtd, *J* = 6.5 Hz, *J* = 6 Hz, *J* = 2.5 Hz, 1H, $COCH_2CH$), 3.03 (s, 3H, NCH_3), 2.97 (dd, *J* = 16 Hz, *J* = 6.5 Hz, 1H, CO*CH*H), 2.65 (dd, *J* = 16 Hz, *J* = 2.5 Hz, 1H, COCH*H*), 1.63–1.52 (m, 2H, $CH_2(CH_2)_4CH_3$), 1.37–1.16 (m, 8H, $CH_2(CH_2)_4CH_3$), 0.86 (t, *J* = 7 Hz, 3H, $(CH_2)_5CH_3$). ^{13}C NMR (100 MHz, $CDCl_3$): δ = 193.5, 150.1, 135.7, 127.5, 119.1, 115.9, 112.8, 61.5, 41.5, 38.1, 31.7, 29.2, 28.5, 26.0, 22.5, 14.0. IR: ν_{max} (cm^{-1}) 2979, 1657, 1611, 1505, 1381, 1279, 1025, 759. LRMS (ESI): *m/z*: 246 (65%, $[M+H]^+$), 268 (100%, $[M+Na]^+$). HRMS (ESI): Found 268.1687 ($M+Na^+$), calculated for $C_{16}H_{23}NNaO$ is 268.1671.

1-Methyl-2-isopentyl-2,3-dihydroquinolin-4(1H)-one (10g)

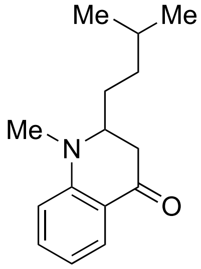

Prepared following general cyclisation procedure from *(E)*-1-(2-((3,4-dimethoxybenzyl)(methyl)amino)phenyl)-6-methylhept-2-en-1-one **9o** (115 mg, 0.30 mmol), trifluoromethanesulfonic acid (80 µL, 0.90 mmol) in DCM (3 mL), then antimony(III) chloride (21 mg, 0.090 mmol) in MeCN (1 mL). Flash chromatography (10% Et_2O in petrol) afforded the title compound as a brown oil (43 mg; 62%). ^{1}H NMR (400 MHz, $CDCl_3$): δ = 7.83 (d, *J* = 8 Hz, 1H, *Ar-H*), 7.37 (dd, *J* = 8.5 Hz, *J* = 8 Hz, 1H, *Ar-H*), 6.66 (dd, *J* = 8.5 Hz, *J* = 8 Hz, 1H, *Ar-H*), 6.59 (d, *J* = 8.5 Hz, 1H, *Ar-H*), 3.47 (tdd, *J* = 7 Hz, *J* = 6 Hz, *J* = 2.5 Hz, 1H, $COCH_2CH$), 3.02 (s, 3H, NCH_3), 2.94 (dd, *J* = 16 Hz, *J* = 6 Hz, 1H, CO*CH*H), 2.63 (dd, *J* = 16 Hz, *J* = 2.5 Hz, 1H, COCH*H*), 1.63–1.51 (m, 2H, $CH_2CH_2CH(CH_3)_2$), 1.46 (m, 1H, $CH(CH_3)_2$), 1.25–1.03 (m, 2H, $CH_2CH_2CH(CH_3)_2$), 0.84 (d, *J* = 6.5 Hz, 3H, $CHCH_3$) and 0.83 (d, *J* = 6.5

Hz, 3H, CHCH_3) overlapping. ^{13}C NMR (100 MHz, $CDCl_3$): δ = 193.4, 150.0, 135.6, 127.3, 119.0, 115.8, 112.7, 61.7, 41.3, 38.0, 34.9, 28.0, 26.1, 22.6, 22.2. IR: ν_{max} (cm^{-1}) 2985, 1666, 1599, 1512, 1380, 1279, 733. LRMS (ESI): *m/z*: 232 (40%, $[M+H]^+$), 254 (100%, $[M+Na]^+$). HRMS (ESI): Found 254.1522 ($M+Na^+$), calculated for $C_{15}H_{21}NNaO$ is 254.1515.

2-(*tert*-Butyl)-1-methyl-2,3-dihydroquinolin-4(1*H*)-one (10h)

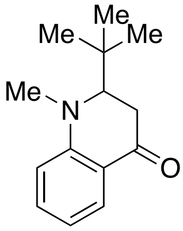

Prepared following general procedure D from (*E*)-1-(2-((3,4-dimethoxybenzyl)(methyl)amino)phenyl)-4,4-dimethylpent-2-en-1-one **9p** (110 mg, 0.30 mmol), trifluoromethanesulfonic acid (80 µL, 0.90 mmol) in DCM (3 mL), then antimony(III) chloride (21 mg, 0.090 mmol) in MeCN (1 mL). Flash chromatography (10% Et_2O in petrol) afforded the title compound as a yellow oil (36 mg; 55%). ^{1}H NMR (400 MHz, $CDCl_3$): δ = 7.77 (dd, *J* = 8 Hz, *J* = 1.5 Hz, 1H, *Ar-H*), 7.35 (ddd, *J* = 8.5 Hz, *J* = 7 Hz, *J* = 1.5 Hz, 1H, *Ar-H*), 6.63–6.56 (m, 2H, *Ar-H*), 3.25 (dd, *J* = 8 Hz, *J* = 1.5 Hz, 1H, C*H*C$(CH_3)_3$), 3.18 (s, 3H, NCH_3), 3.00 (dd, *J* = 16.5 Hz, *J* = 8 Hz, 1H, COC*H*H), 2.82 (dd, *J* = 16.5 Hz, *J* = 1.5 Hz, 1H, COCH*H*), 0.91 (s, 9H, C$(CH_3)_3$). ^{13}C NMR (100 MHz, $CDCl_3$): δ = 194.1, 151.0, 135.6, 126.9, 119.3, 115.3, 112.4, 69.9, 42.6, 39.2, 38.5, 27.8. IR: ν_{max} (cm^{-1}) 2955, 1671, 1605, 1562, 1498, 1434, 1395, 1347, 1292, 1162, 1096, 1037, 753. LRMS (ESI): *m/z*: 218 (95%, $[M+H]^+$), 240 (20%, $[M+Na]^+$), 457 (100%, $[2M+Na]^+$). HRMS (ESI): Found 240.1355 ($M+Na^+$), calculated for $C_{14}H_{19}NNaO$ is 240.1359.

2-(3-Chloropropyl)-1-methyl-2,3-dihydroquinolin-4(1*H*)-one (10i)

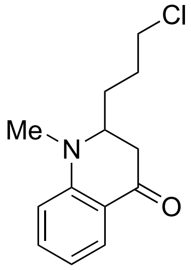

Prepared following general procedure D from (*E*)-6-chloro-1-(2-((3,4-dimethoxybenzyl)(methyl)amino)phenyl)hex-2-en-1-one **9q** (116 mg, 0.30 mmol), trifluoromethanesulfonic acid (80 µL, 0.90 mmol) in DCM (3 mL), then antimony(III) chloride (21 mg, 0.090 mmol) in MeCN (1 mL). Flash chromatography (20% Et_2O in petrol) afforded the title compound as a yellow oil (30 mg; 42%). ^{1}H NMR (400MHz, $CDCl_3$): δ = 7.84 (dd, *J* = 8 Hz, *J* = 1.5 Hz, 1H, *Ar-H*), 7.39 (ddd, *J* = 8.5 Hz, *J* = 7 Hz, *J* = 1.5 Hz, 1H, *Ar-H*), 6.69 (ddd, *J* = 8 Hz, *J* = 7 Hz, *J* = 1 Hz, 1H, *Ar-H*), 6.60 (app d, *J* = 8.5 Hz, 1H, *Ar-H*), 3.57 (tdd, *J* = 7 Hz, *J* = 6 Hz, *J* = 2.5 Hz, 1H, C*H*$(CH_2)_3$Cl), 3.52–3.44 (m, 2H, CH_2Cl), 3.05 (s, 3H, –NCH_3), 3.01 (dd, *J* = 16 Hz, *J* = 6 Hz, 1H, COCH*H*), 2.61 (dd, *J* = 16 Hz, *J* = 2.5 Hz, 1H, COC*H*H), 1.88–1.78 (m, 2H, $CH_2(CH_2)_2$Cl), 1.71–1.64 (m, 2H, $CH_2CH_2CH_2$Cl). ^{13}C NMR (400MHz,

$CDCl_3$): δ = 193.0, 149.9, 135.9, 127.5, 119.0, 116.2, 112.9, 60.8, 44.5, 41.5, 38.2, 29.0, 26.1. IR: ν_{max} (cm^{-1}) 2938, 1671, 1603, 1562, 1494, 1460, 1340, 1316, 1269, 1207, 1164, 1037, 757. LRMS (ESI): *m/z* 238 (100%, $[M+H]^+$). HRMS (ESI): Found 260.0820 ($M+Na^+$), calculated for $C_{13}H_{16}ClNNaO$ is 260.0813.

4-(1-Methyl-4-oxo-1,2,3,4-tetrahydroquinolin-2-yl)butanenitrile (10j)

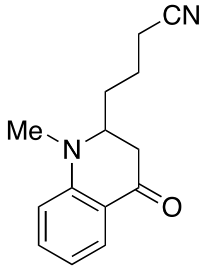

Prepared following general procedure D from (*E*)-7-(2-((3,4-dimethoxybenzyl)(methyl)amino)phenyl)-7-oxohept-5-enenitrile **9r** (114 mg, 0.30 mmol), trifluoromethanesulfonic acid (80 μL, 0.90 mmol) in DCM (3 mL), then antimony(III) chloride (21 mg, 0.090 mmol) in MeCN (1 mL). Flash chromatography (60% Et_2O in petrol) afforded the title compound as a yellow oil (45 mg, 66%). ^1H NMR (400 MHz, $CDCl_3$): δ = 7.83 (dd, *J* = 8 Hz, *J* = 1.5 Hz, 1H, *Ar-H*), 7.40 (ddd, *J* = 8.5 Hz, *J* = 7 Hz, *J* = 1.5 Hz, 1H, *Ar-H*), 6.70 (ddd, *J* = 8 Hz, *J* = 7 Hz, *J* = 1 Hz, 1H, *Ar-H*), 6.61 (d, *J* = 8.5 Hz, 1H, *Ar-H*), 3.62–3.55 (m, 1H, C*H*(CH_2)$_3$CN), 3.05 (s, 3H, NC*H*$_3$), 3.02 (dd, *J* = 16 Hz, *J* = 6 Hz, 1H, COC*H*H), 2.58 (dd, *J* = 16 Hz, *J* = 2.5 Hz, 1H, COCH*H*), 2.39–2.24 (m, 1H, C*H*$_2$CN), 1.83–1.53 (m, 4H, (C*H*$_2$)$_2$$CH_2$CN). ^{13}C NMR (100 MHz, $CDCl_3$): δ = 192.7, 149.7, 136.0, 127.4, 119.1, 119.0, 116.4, 112.9, 60.6, 41.4, 38.2, 28.0, 22.0, 17.2. IR: ν_{max} (cm^{-1}) 2935, 1667, 1601, 1561, 1492, 1460, 1434, 1344, 1277, 1205, 1163, 1095, 1037, 993, 757. LRMS (ESI): *m/z* 229 (100%, $[M+H]^+$), 251 (50%, $[M+Na]^+$), 479 (75%, $[2M+Na]^+$). HRMS (ESI): Found 251.1162 ($M+Na^+$), calculated for $C_{14}H_{16}N_2NaO$ is 251.1155.

1-Methyl-2-ferrocenyl-2,3-dihydroquinolin-4(1H)-one (10l)

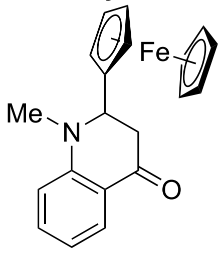

Prepared following general procedure D from *(E)*-1-(2-((3,4-dimethoxybenzyl)(methyl)amino)phenyl)-3-ferrocenylprop-2-en-1-one **9t** (149 mg, 0.30 mmol), trifluoromethanesulfonic acid (80 μL, 0.90 mmol) in DCM (3 mL), then antimony(III) chloride (21 mg, 0.090 mmol) in MeCN (1 mL). Flash chromatography (10% Et_2O in petrol) afforded the title compound as a red oil (45 mg, 66%). ^1H NMR (400MHz, $CDCl_3$): δ = 7.90 (d, *J* = 7.5 Hz, 1H, *Ar-H*), 7.34 (t, *J* = 7 Hz, 1H, *Ar-H*), 6.69 (t, *J* = 7.5 Hz, 1H, *Ar-H*), 6.53 (d, *J* = 8.5 Hz, 1H, *Ar-H*), 4.48 (bs, 1H, $COCH_2$C*H*), 4.19 (s, 5H, *Fc-H*), 4.16–4.05 (m, 4H, *Fc-H*), 3.28 (d, *J* = 16 Hz, 1H, COC*H*H), 3.18 (d, *J* = 16 Hz, 1H, COCH*H*), 2.97 (s, 3H, NC*H*$_3$). ^{13}C NMR (400MHz, $CDCl_3$): δ = 193.4, 149.9, 135.7, 127.4, 119.9, 117.4, 113.1, 86.0, 68.8

(5C), 68.5, 68.4, 67.6, 66.7, 60.6, 43.6, 37.9. IR: ν_{max} (cm^{-1}) 2979, 1657, 1611, 1505, 1381, 1279, 1095, 1025, 910, 840, 759. LRMS (ESI): *m/z*: 368 (100%, $[M+H]^+$). HRMS (ESI): Found 368.0713 ($M+H^+$), calculated for $C_{20}H_{19}FeNO$ is 368.0708.

1-Methyl-2-(4-fluorophenyl)-2,3-dihydroquinolin-4(1H)-one (10m)

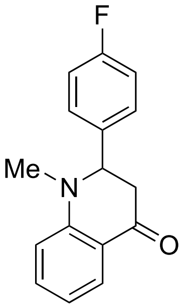

Prepared following general procedure D from *(E)*-1-(2-((3,4-dimethoxybenzyl)(methyl)amino)phenyl)-3-(4-fluorophenyl)prop-2-en-1-one **9u** (122 mg, 0.30 mmol), trifluoromethanesulfonic acid (80 μL, 0.90 mmol) in DCM (3 mL), then antimony(III) chloride (21 mg, 0.090 mmol) in MeCN (1 mL). Flash chromatography (10% Et_2O in petrol) afforded the title compound as a red oil (60 mg; 79%). ^1H NMR (400MHz, $CDCl_3$): δ = 7.87 (dd, *J* = 8 Hz, *J* = 2 Hz, 1H, *Ar-H*), 7.47 (ddd, *J* = 8.5 Hz, *J* = 8 Hz, *J* = 2 Hz, 1H, *Ar-H*), 7.16–7.12 (m, 2H, *Ar-H*), 7.01–6.96 (m, 2H, *Ar-H*), 6.78 (d, *J* = 8.5 Hz, 1H, *Ar-H*), 6.77 (dd, *J* = 8.5 Hz, *J* = 8 Hz, 1H, *Ar-H*), 4.67 (t, *J* = 6 Hz, 1H, $COCH_2CH$), 3.17 (dd, *J* = 16 Hz, *J* = 6 Hz, 1H, CO*CH*H), 2.94 (s, 3H, NCH_3.), 2.89 (dd, *J* = 16 Hz, *J* = 6 Hz, 1H, COCH*H*). ^{13}C NMR (400MHz, $CDCl_3$): δ = 192.2, 162.2 (d, ^1J_F = 245 Hz), 151.5, 136.1, 135.7 (d, ^4J_F = 3 Hz), 128.2 (d, ^3J_F = 8.5 Hz), 127.7, 119.8, 116.8, 115.9 (d, ^2J_F = 22 Hz), 112.9, 64.1, 45.5, 37.8. IR: ν_{max} (cm^{-1}) 2912, 2362, 1674, 1603, 1564, 1509, 1435, 1348, 1319, 1287, 1224, 1094, 1039, 908, 840, 734. LRMS (ESI): *m/z*: 278 (100%, $[M+Na]^+$). HRMS (ESI): Found 278.0969 ($M+Na^+$), calculated for $C_{16}H_{14}FNNaO$ is 278.0951.

1,3-Dimethyl-2-phenyl-2,3-dihydroquinolin-4(1*H*)-one (10n and 10n')

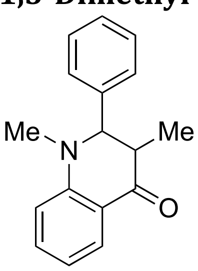

Prepared following general procedure D from (*E*)-1-(2-((3,4-dimethoxybenzyl)(methyl)amino)phenyl)-2-methyl-3-phenylprop-2-en-1-one **9u** (121 mg, 0.30 mmol), trifluoromethanesulfonic acid (80 μL, 0.90 mmol) in DCM (3 mL), then antimony(III) chloride (21 mg, 0.090 mmol) in MeCN (1 mL). Flash chromatography (2% Et_2O in petrol) afforded the title compound as a 4:1 mixture of diastereomers (48 mg, 64%). Pure diastereomers were available for characterization purposes. **10n:** ^1H NMR (400 MHz, $CDCl_3$): δ = 7.36 (t, *J* = 8 Hz, 1H, *Ar-H*), 7.34–7.30 (m, 2H, *Ar-H*), 7.28–7.20 (m, 2H, *Ar-H*), 7.19–7.15 (m, 2H, *Ar-H*), 6.47 (d, *J* = 8 Hz, 1H, *Ar-H*), 6.33 (d, *J* = 7.5 Hz, 1H, *Ar-H*), 3.91 (d, *J* = 4.5 Hz, 1H, C*H*Ph), 2.96 (s, 3H, NCH_3), 2.61 (qd, *J* = 7.5 Hz, *J* = 4.5 Hz, 1H, *CH*Me), 1.34 (d, *J* = 7.5 Hz, 3H, $CHCH_3$). ^{13}C NMR (100 MHz, $CDCl_3$): δ = 208.6, 157.5, 149.1, 143.2,

137.1, 128.7, 128.0, 126.8, 118.8, 112.1, 107.2, 53.7, 52.8, 29.1, 14.9. IR: ν_{max} (cm^{-1}) 2928, 1673, 1594, 1518, 1478, 1454, 1409, 1328, 1231, 1171, 755. LRMS (ESI): *m/z*: 252 (100%, $[M+H]^+$), 274 (15%, $[M+Na]^+$). HRMS (ESI): Found 274.1200 ($M+Na^+$), calculated for $C_{17}H_{17}NNaO$ is 274.1202. **10n':** ^{1}H NMR (400 MHz, $CDCl_3$): δ = 7.86 (dd, *J* = 8 Hz, *J* = 1.5 Hz, 1H, *Ar-H*), 7.41 (ddd, *J* = 8.5 Hz, *J* = 7 Hz, *J* = 1.5 Hz, 1H, *Ar-H*), 7.30–7.18 (m, 3H, *Ar-H*), 7.07–7.05 (m, 2H, *Ar-H*), 6.73 (ddd, *J* = 8 Hz, *J* = 7.5 Hz, *J* = 1 Hz, 1H, *Ar-H*), 6.65 (d, *J* = 8.5 Hz, 1H, *Ar-H*), 4.49 (d, *J* = 7 Hz, 1H, C*H*Ph), 3.42 (qt, *J* = 7 Hz, 1H, *CH*Me), 2.92 (s, 3H, NC*H₃*), 1.04 (d, *J* = 7 Hz, 3H, CHC*H₃*). ^{13}C NMR (100 MHz, $CDCl_3$): δ = 195.5, 151.1, 136.8, 135.6, 128.6, 128.1, 127.6, 127.4, 119.4, 116.2, 112.2, 71.0, 45.4, 37.9, 11.2. IR: ν_{max} (cm^{-1}) 2934, 1674, 1605, 1565, 1493, 1454, 1347, 1314, 1256, 1166, 732. LRMS (ESI): *m/z*: 252 (100%, $[M+H]^+$), 274 (35%, $[M+Na]^+$). HRMS (ESI): Found 274.1196 ($M+Na^+$), calculated for $C_{17}H_{17}NNaO$ is 274.1202. The major diastereomer (**10n'**) was assigned as the *trans* diastereomer on the basis of the coupling constant.

8-Methoxy-1-methyl-2-phenyl-2,3-dihydroquinolin-4(1H)-one (10o)

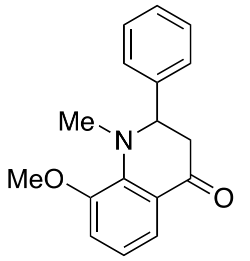

Prepared following general procedure D from (*E*)-1-(2-((3,4-dimethoxybenzyl)(methyl)amino)-3-methoxyphenyl)-3-phenylprop-2-en-1-one **9h** (125 mg, 0.30 mmol), trifluoromethanesulfonic acid (80 µL, 0.90 mmol) in DCM (3 mL), then antimony(III) chloride (21 mg, 0.090 mmol) in MeCN (1 mL). Flash chromatography (40% Et_2O in petrol) afforded the title compound as a yellow oil (68 mg, 85%). ^{1}H NMR (400 MHz, $CDCl_3$): δ = 7.31–7.26 (m, 3H, *Ar-H*), 7.22 (ddt, *J* = 8.5 Hz, *J* = 6.5 Hz, *J* = 1.5 Hz, 1H, *Ar-H*), 7.15–7.11 (m, 2H, *Ar-H*), 6.92 (d, *J* = 8 Hz, 1H, *Ar-H*), 6.32 (dd, *J* = 8 Hz, *J* = 1 Hz, 1H, *Ar-H*), 4.36 (dd, *J* = 8 Hz, *J* = 4 Hz, 1H, C*H*Ph), 3.78 (s, 3H, OC*H₃*), 3.21 (s, 3H, NC*H₃*), 3.17 (dd, *J* = 19 Hz, *J* = 8 Hz, 1H, COC*H*H), 2.65 (dd, *J* = 19 Hz, *J* = 4 Hz, 1H, COCH*H*). ^{13}C NMR (100 MHz, $CDCl_3$): δ = 206.5, 151.2, 146.7, 144.5, 140.9, 128.7, 127.5, 126.6, 121.5, 120.5, 112.6, 57.1, 47.5, 43.1, 32.9. IR: ν_{max} (cm^{-1}) 2950, 1667, 1598, 1525, 1495, 1392, 1351, 1245, 1213, 1102, 1037, 816, 761. LRMS (ESI): *m/z*: 268 (100%, $[M+H]^+$). HRMS (ESI): Found 290.1142 ($M+Na^+$), calculated for $C_{17}H_{17}NNaO_2$ is 290.1151.

6,7-Dimethoxy-1-methyl-2-phenyl-2,3-dihydroquinolin-4(1H)-one (10p)

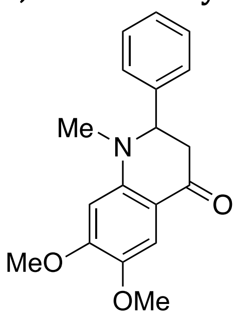

Prepared following general procedure D from (*E*)-1-(2-((3,4-dimethoxybenzyl)(methyl)amino)-4,5-dimethoxyphenyl)-3-phenylprop-2-en-1-one **9i** (134 mg, 0.30 mmol), trifluoromethanesulfonic acid (80 µL, 0.90 mmol) in DCM (3 mL), then antimony(III) chloride (21 mg, 0.090 mmol) in MeCN (1 mL). Flash chromatography (60% Et_2O in petrol) afforded the title compound as a yellow oil (62 mg, 70%). ^1H NMR (400 MHz, $CDCl_3$): δ = 7.36 (s, 1H, *Ar-H*), 7.33–7.24 (m, 3H, *Ar-H*), 7.19–7.17 (m, *J* = 2H, *Ar-H*), 6.23 (s, 1H, *Ar-H*), 4.61 (t, *J* = 6.5 Hz, 1H, C*H*Ph), 3.97 (s, 3H, OC*H₃*), 3.86 (s, 3H, OC*H₃*), 3.09 (dd, *J* = 16 Hz, *J* = 6 Hz, 1H, COC*H*H), 2.92 (s, 3H, NC*H₃*), 2.88 (dd, *J* = 16 Hz, *J* = 7 Hz, 1H, COCH*H*). ^{13}C NMR (100 MHz, $CDCl_3$): δ = 190.5, 156.3, 149.0, 141.6, 140.2, 128.9, 127.8, 126.7, 112.3, 108.6, 95.8, 65.3, 56.1, 55.9, 45.3, 38.1. IR: ν_{max} (cm^{-1}) 2926, 1739, 1655, 1615, 1567, 1453, 1422, 1248, 1040, 910, 812, 731. LRMS (ESI): *m/z*: 298 (100%, $[M+H]^+$), 320 (40%, $[M+Na]^+$). HRMS (ESI): Found 320.1253 ($M+Na^+$), calculated for $C_{18}H_{19}NNaO_3$ is 320.1257.

1-Methyl-2-phenyl-7-(trifluoromethyl)-2,3-dihydroquinolin-4(1*H*)-one (10q)

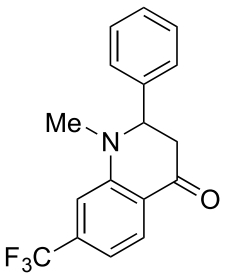

Prepared following general procedure D from (*E*)-1-(2-((3,4-dimethoxybenzyl)(methyl)amino)-4-trifluoromethyl)-3-phenylprop-2-en-1-one **9k** (137 mg, 0.30 mmol), trifluoromethanesulfonic acid (80 µL, 0.90 mmol) in DCM (3 mL), then antimony(III) chloride (21 mg, 0.090 mmol) in MeCN (1 mL). Flash chromatography (10% Et_2O in petrol) afforded the title compound as a yellow oil (66 mg; 72%). ^1H NMR (400MHz, $CDCl_3$): δ = 7.93 (d, *J* = 8 Hz, 1H, *Ar-H*), 7.33–7.27 (m, 3H, *Ar-H*), 7.14–7.12 (m, 2H, *Ar-H*), 6.99–6.95 (m, 2H, *Ar-H*), 4.74 (t, *J* = 6 Hz, 1H, *CH*Ph), 3.21 (dd, *J* = 16 Hz, *J* = 6 Hz, 1H, COC*H*H), 3.00 (s, 3H, N*CH₃*), 2.96 (dd, *J* = 16 Hz, *J* = 6 Hz, 1H, COCH*H*). ^{13}C NMR (400MHz, $CDCl_3$): δ = 191.7, 151.2, 139.1, 136.9 (q, $^2J_{CF_3}$ = 32 Hz), 129.1, 128.5, 128.1, 126.4, 123.7 (q, $^1J_{CF_3}$ = 274 Hz), 121.5, 112.7 (q, $^3J_{CF_3}$ = 4 Hz), 109.7 (q, $^3J_{CF_3}$ = 4 Hz), 64.5, 45.1, 38.0. IR: ν_{max} (cm^{-1}) 2918, 1686, 1619, 1571, 1494, 1450, 1426, 1336, 1312, 1213, 1168, 1127, 1084, 1039, 856, 818, 735, 702. LRMS (ESI): *m/z* 305 (100%, $[M+H]^+$), 328 (80%, $[M+Na]^+$). HRMS (ESI): Found 328.0918 ($M+Na^+$), calculated for $C_{17}H_{14}F_3NNaO$ is 328.0920.

1-Methyl-2-phenyl-7-bromo-2,3-dihydroquinolin-4(1*H*)-one (10r)

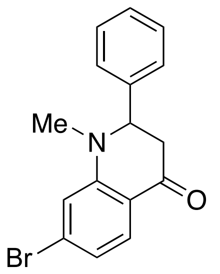

Prepared following general procedure D from (*E*)-1-(4-Bromo-2-((3,4-dimethoxybenzyl)(methyl)amino)phenyl)-3-phenylprop-2-en-1-one **9l** (140 mg, 0.30 mmol), trifluoromethanesulfonic acid (80 µL, 0.90 mmol) in DCM (3 mL), then antimony(III) chloride (21 mg, 0.090 mmol) in MeCN (1 mL). Flash chromatography (10% Et_2O in petrol) afforded the title compound as a yellow oil (57 mg; 60%). ^1H NMR (400MHz, $CDCl_3$): δ = 7.69 (d, *J* = 8.5 Hz, 1H, *Ar-H*), 7.32–7.25 (m, 3H, *Ar-H*), 7.13–7.11 (m, 2H, *Ar-H*), 6.93 (d, *J* = 1.5 Hz, 1H, *Ar-H*), 6.87 (dd, *J* = 8.5 Hz, *J* = 1.5 Hz, 1H, *Ar-H*), 4.69 (t, *J* = 6 Hz, 1H, *CH*Ph), 3.17 (dd, *J* = 16 Hz, *J* = 6 Hz, 1H, COC*H*H), 2.94 (s, 3H, N*CH₃*), 2.90 (dd, *J* = 16 Hz, *J* = 6 Hz, 1H, COCH*H*). ^{13}C NMR (400MHz, $CDCl_3$): δ = 191.5, 152.0, 139.3, 131.3, 129.1 (2C), 128.0, 126.4, 119.9, 118.5, 115.6, 64.6, 45.1, 38.0. IR: ν_{max} (cm^{-1}) 2915, 1678, 1592, 1547, 1490, 1451, 1419, 1284, 1212, 1085, 1038, 909, 839, 801, 739, 701. LRMS (ESI): *m/z* 316 (70%, [[^{79}Br] M+H]$^+$), 318 (70%, [[^{81}Br] M+H]$^+$), 338 (100%, [[^{79}Br] M+Na]$^+$), 340 (95%, [[^{81}Br] M+Na]$^+$). HRMS (ESI): Found 338.0147 ([^{79}Br] M+Na$^+$), 340.0133 ([^{81}Br] M+Na$^+$), calculated for $C_{16}H_{14}{}^{79}BrNNaO$ is 338.0151, calculated for $C_{16}H_{14}{}^{81}BrNNaO$ is 340.0131.

4-Methyl-5-phenyl-5,6-dihydrothieno[3,2-b]pyridin-7(4H)-one (10s)

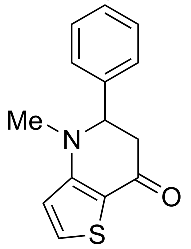

Prepared following general procedure D from *(E)*-1-(3-((3,4-dimethoxybenzyl)(methyl)amino)thiophen-2-yl)-3-phenylprop-2-en-1-one **9m** (118 mg, 0.50 mmol), trifluoromethanesulfonic acid (80 µL, 0.90 mmol) in DCM (3 mL), then antimony(III) chloride (21 mg, 0.090 mmol) in MeCN (1 mL). Flash chromatography (10% Et_2O in petrol) afforded the title compound as a yellow oil (56 mg; 77%). ^1H NMR (400MHz, $CDCl_3$): δ = 7.62 (d, *J* = 5.5 Hz, 1H, SC*H*), 7.38–7.29 (m, 5H, *Ar-H*), 6.69 (d, *J* = 5.5 Hz, 1H, SCHC*H*), 4.55 (t, *J* = 8 Hz, 1H, C*H*Ph), 2.89 (d, *J* = 8 Hz, 2H, COC*H₂*), 2.83 (s, 3H, NC*H₃*). ^{13}C NMR (400MHz, $CDCl_3$): δ = 184.8, 159.4, 139.5, 136.3, 128.9, 128.1, 127.0, 117.2, 113.2, 66.8, 45.7, 37.9. IR: ν_{max} (cm^{-1}) 3080, 2920, 1635, 1544, 1438, 1410, 1277, 1246, 1204, 1060, 1024, 937, 734, 703. LRMS (ESI): *m/z*: 244 (70%, [M+H]$^+$), 266 (100%, [M+Na]$^+$). HRMS (ESI): Found 266.0606 (M+Na$^+$), calculated for $C_{14}H_{13}NNaOS$ is 266.0610.

References

[1] N. Philippe, F. Denivet, J.-L. Vasse, J. S.-d. O. Santos, V. Levacher, G. Dupas, *Tetrahedron* **2003**, *59*, 8049–8056.

[2] K. A. Neidigh, M. A. Avery, J. S. Williamson, S. Bhattacharyya, *J. Chem. Soc., Perkin Trans. 1* **1998**, 2527–2532.

[3] a) E. Tzur, A. Szadkowska, A. Ben-Asuly, A. Makal, I. Goldberg, K. Woźniak, K. Grela, N. G. Lemcoff, *Chem. Eur. J.* **2010**, *16*, 8726–8737; b) E. V. D´yachenko, T. V. Glukhareva, E. F. Nikolaenko, A. V. Tkachev, Y. Y. Morzherin, *Russ. Chem. Bull.* **2004**, *53*, 1240–1247.

[4] E. Kim, M. Koh, B. J. Lim, S. B. Park, *J. Am. Chem. Soc.* **2011**, *133*, 6642–6649.

[5] S. Murarka, C. Zhang, M. D. Konieczynska, D. Seidel, *Org. Lett.* **2009**, *11*, 129–132.

[6] J. S. Baum, M. E. Condon, D. A. Shook, *J. Org. Chem.* **1987**, *52*, 2983–2988.

[7] a) M. Vázquez, M. R. Bermejo, M. Licchelli, A. M. González-Noya, R. M. Pedrido, C. Sangregorio, L. Sorace, A. M. García-Deibe, J. Sanmartín, *Eur. J. Inorg. Chem.* **2005**, 3479–3490; b) W. K. Anderson, D. K. Dalvie, *J. Heterocycl. Chem.* **1993**, *30*, 1533–1536.

[8] a) P.-H. Park, H. S. Kim, X. Y. Jin, F. Jin, J. Hur, G. Ko, D. H. Sohn, *Eur. J. Pharmacol.* **2009**, *606*, 215–224; b) A. V. Dubrovskiy, R. C. Larock, *J. Org. Chem.* **2012**, *77*, 11232–11256.

[9] F. Gao, K. F. Johnson, J. B. Schlenoff, *J. Chem. Soc., Perkin Trans. 2* **1996**, 269–274.

[10] J. A. Donnelly, D. F. Farrell, *J. Org. Chem.* **1990**, *55*, 1757–1761.

[11] a) J. Li, L. Jin, C. Yu, W. Su, *J. Chem. Res.* **2009**, 170–173; b) B.-L. Lei, C.-H. Ding, X.-F. Yang, X.-L. Wan, X.-L. Hou, *J. Am. Chem. Soc.* **2009**, *131*, 18250–18251.

[12] K. Kanagaraj, K. Pitchumani, *J. Org. Chem.* **2013**, *78*, 744–751.

[13] a) D.-F. Chen, Z.-Y. Han, Y.-P. He, J. Yu, L.-Z. Gong, *Angew. Chem. Int. Ed.* **2012**, *51*, 12307–12310; b) M. Rueping, S. A. Moreth, M. Bolte, *Z. Naturforsch.* **2012**, *67b*, 1021–1029.

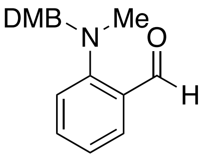

8d

^{1}H NMR (400 MHz, $CDCl_3$)

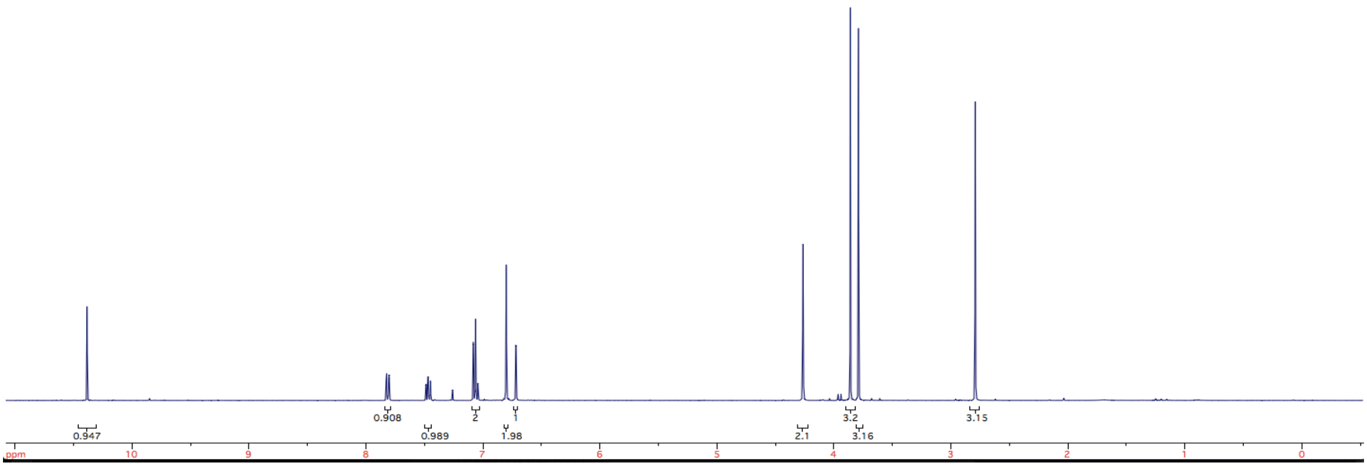

^{13}C NMR (100 MHz, $CDCl_3$)

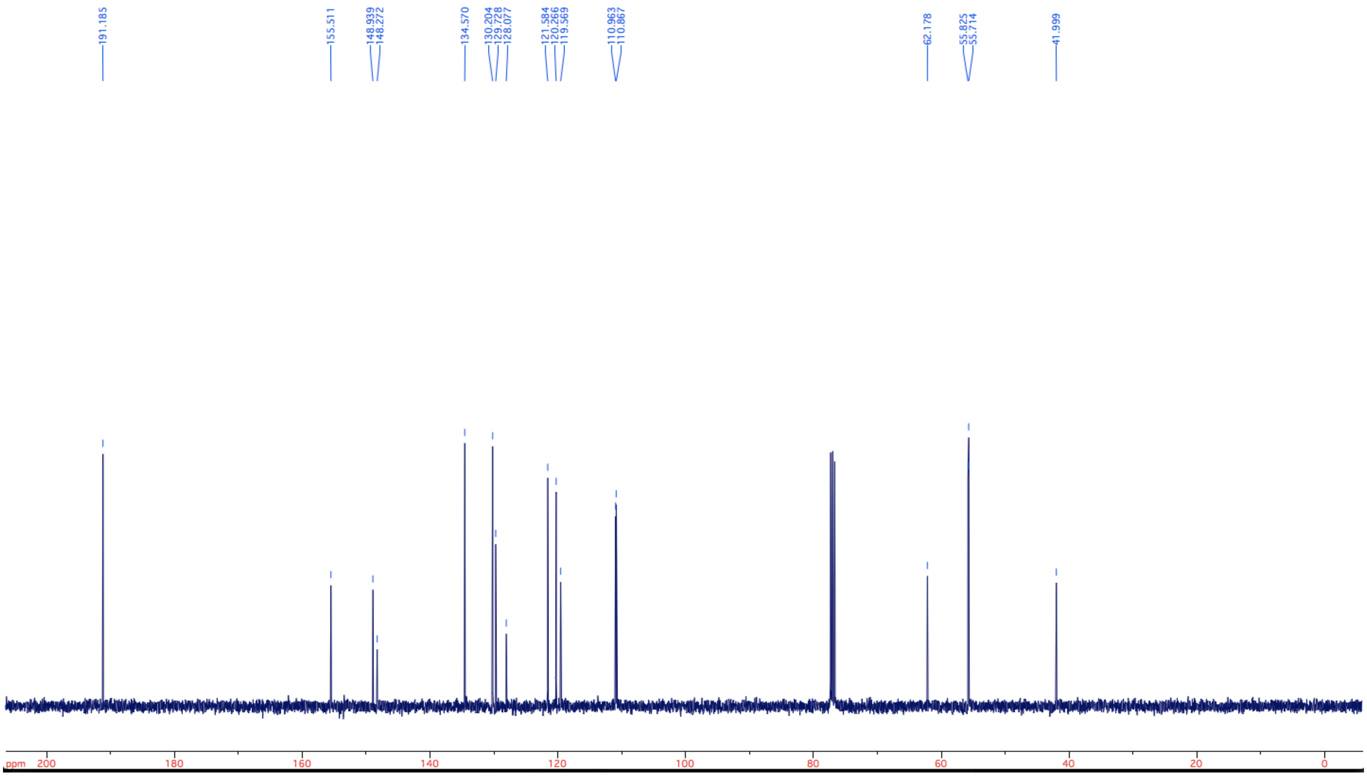

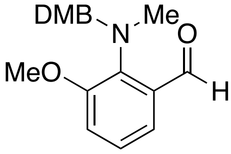

8g

^{1}H NMR (400 MHz, $CDCl_3$)

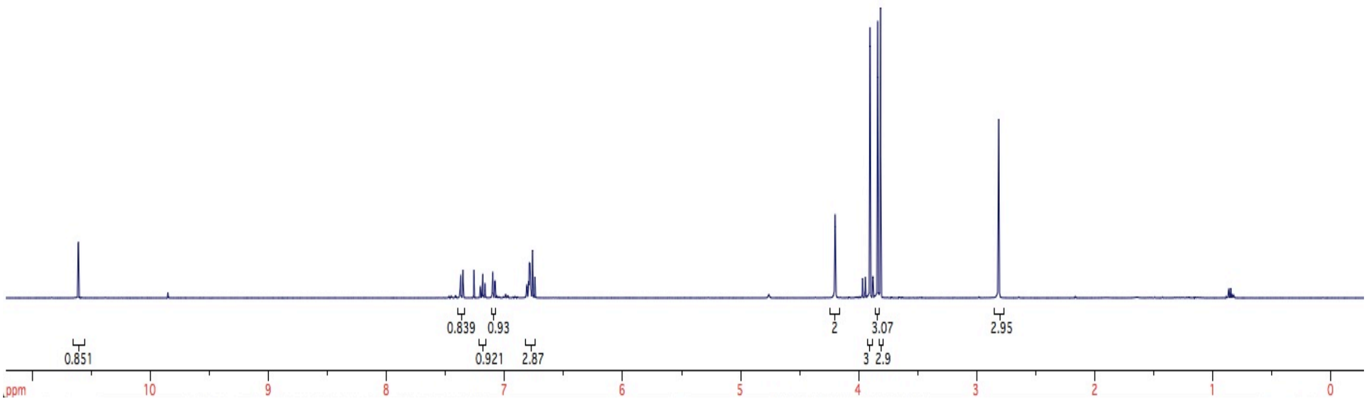

^{13}C NMR (100 MHz, $CDCl_3$)

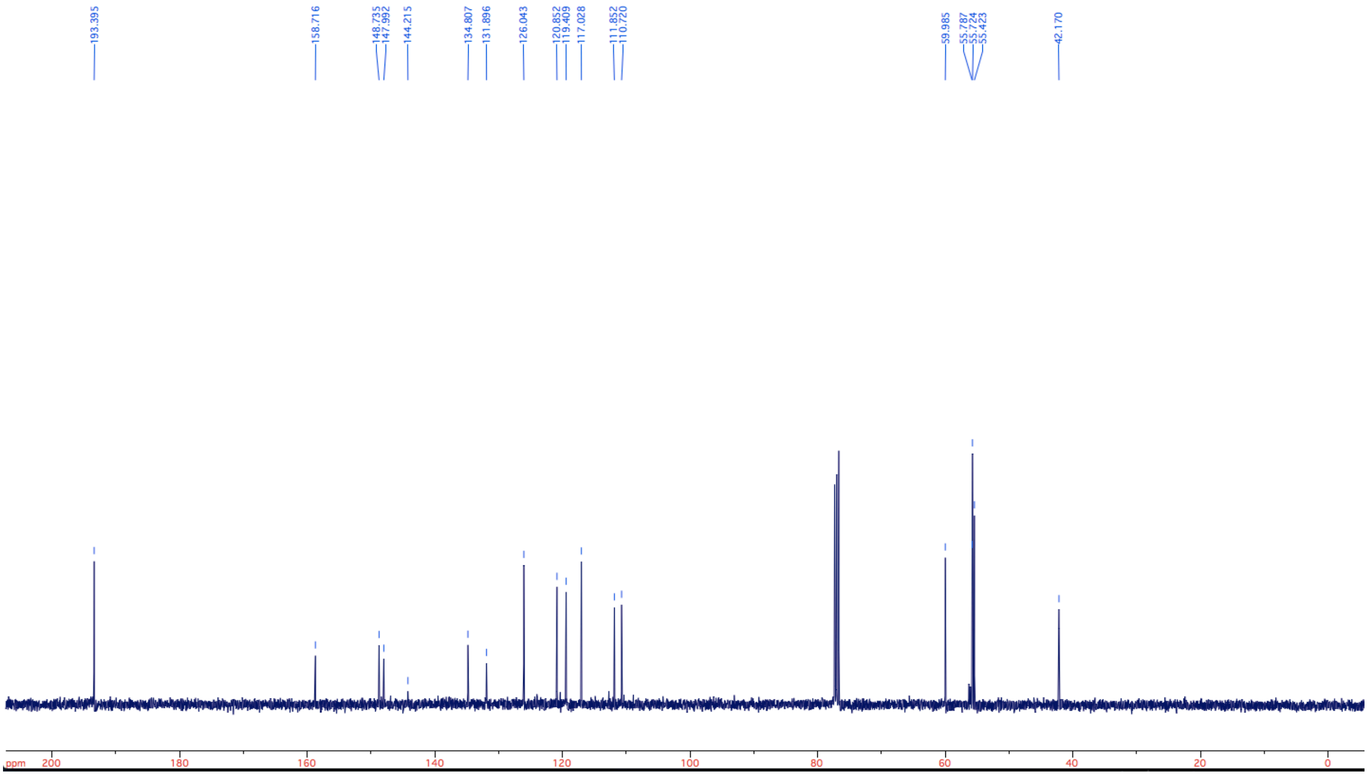

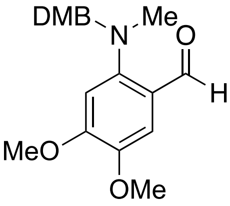

8h

^{1}H NMR (400 MHz, $CDCl_3$)

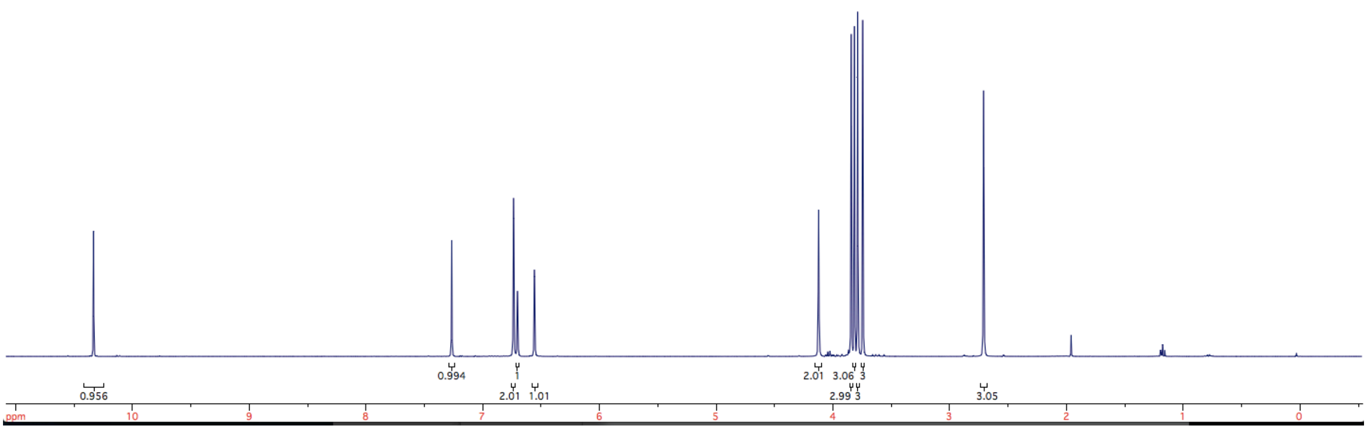

^{13}C NMR (100 MHz, $CDCl_3$)

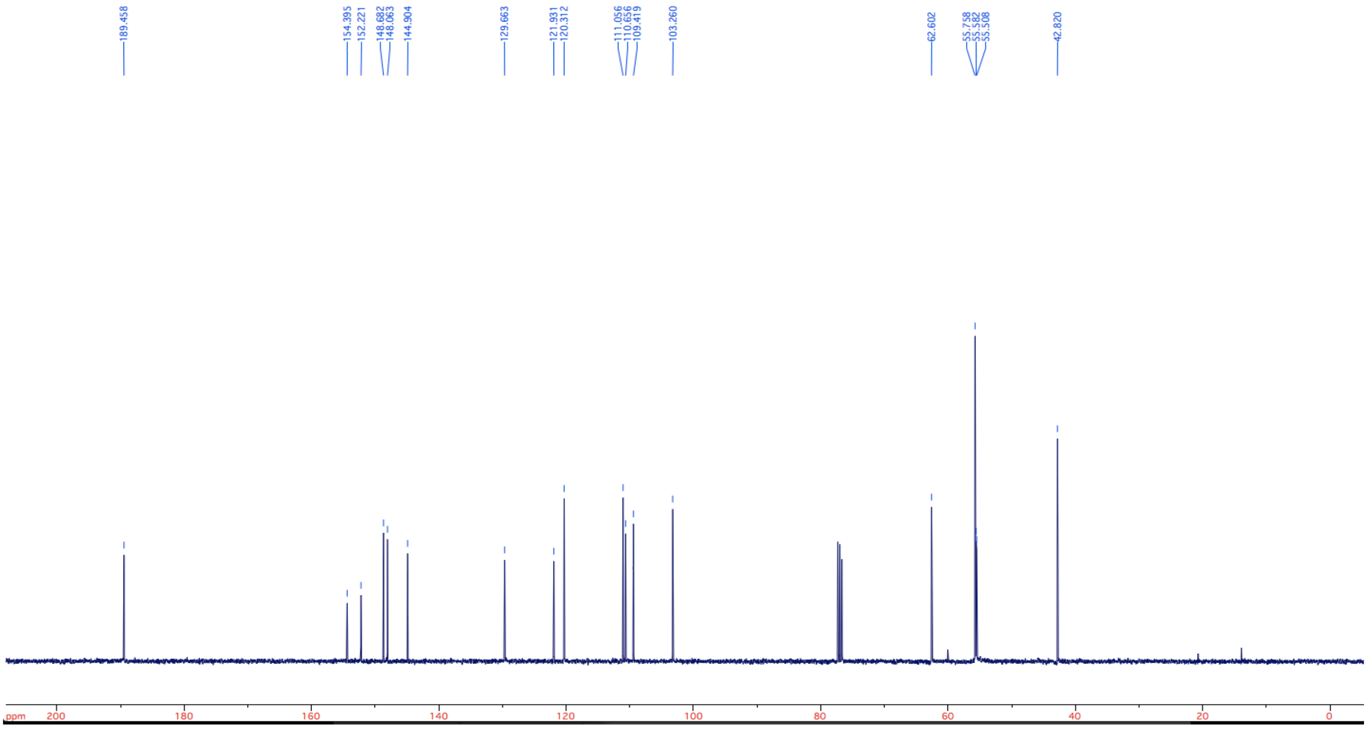

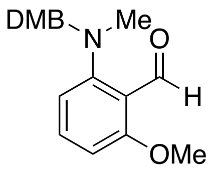

8i

^{1}H NMR (400 MHz, $CDCl_3$)

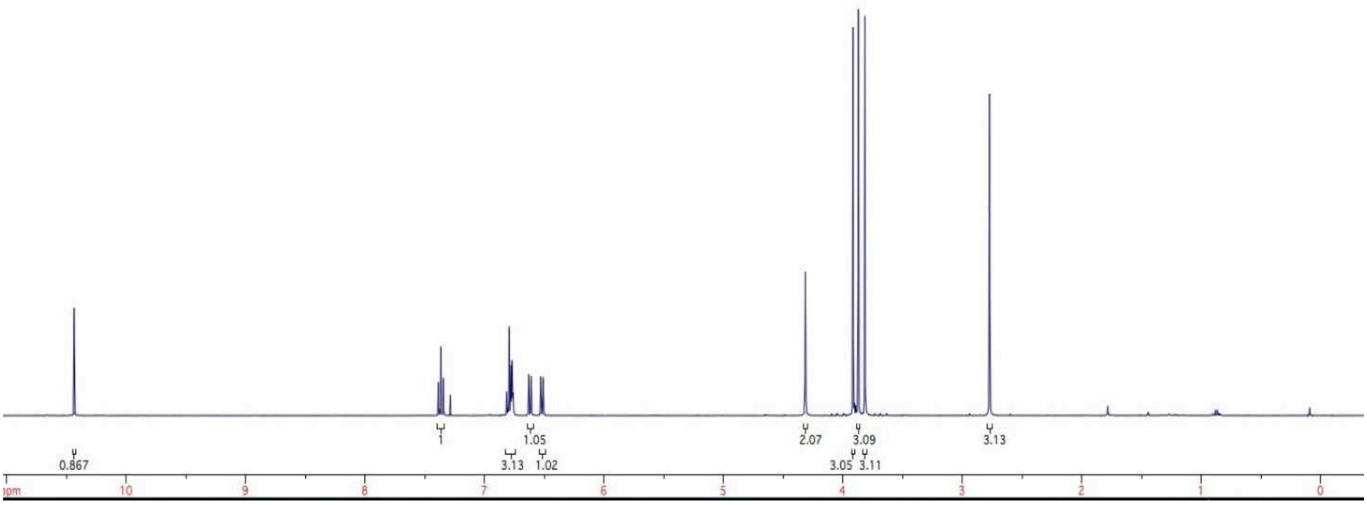

^{13}C NMR (100 MHz, $CDCl_3$)

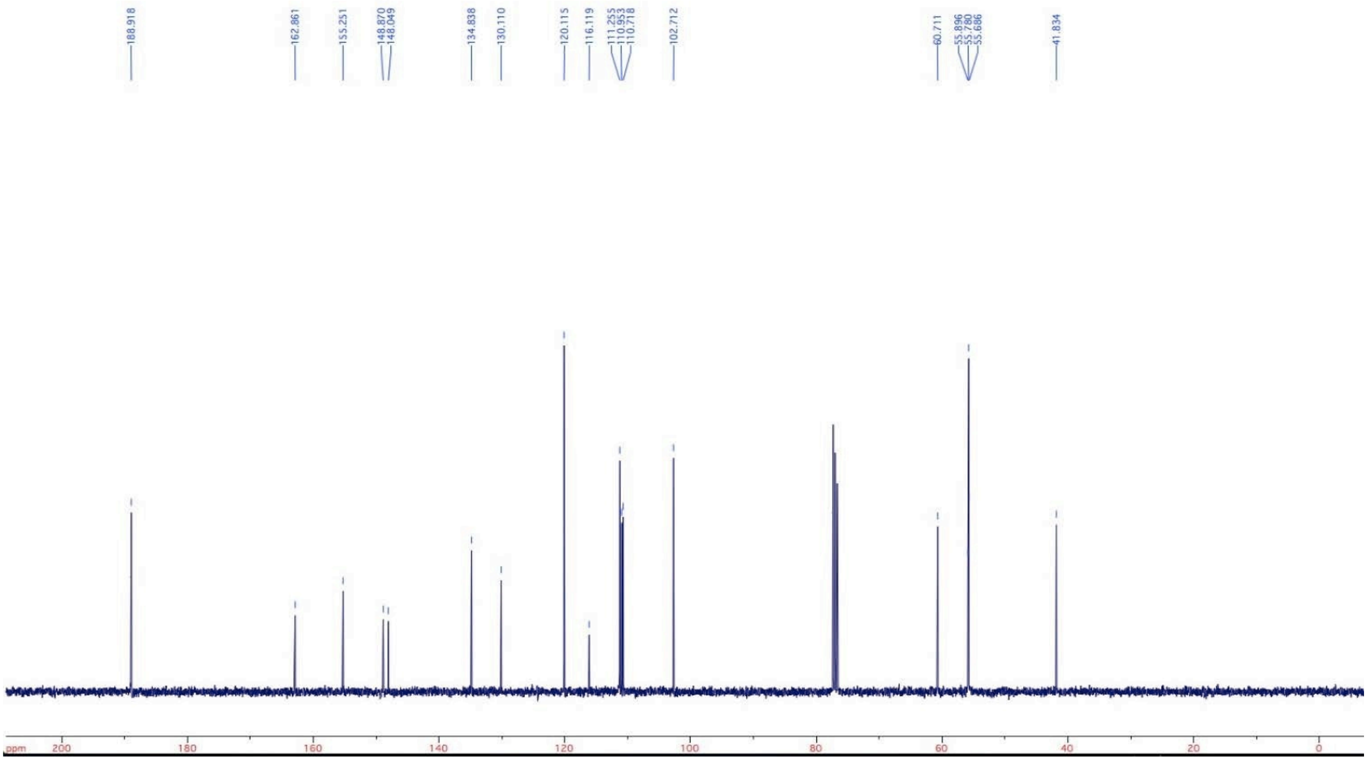

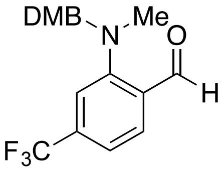

8j

^{1}H NMR (400 MHz, $CDCl_3$)

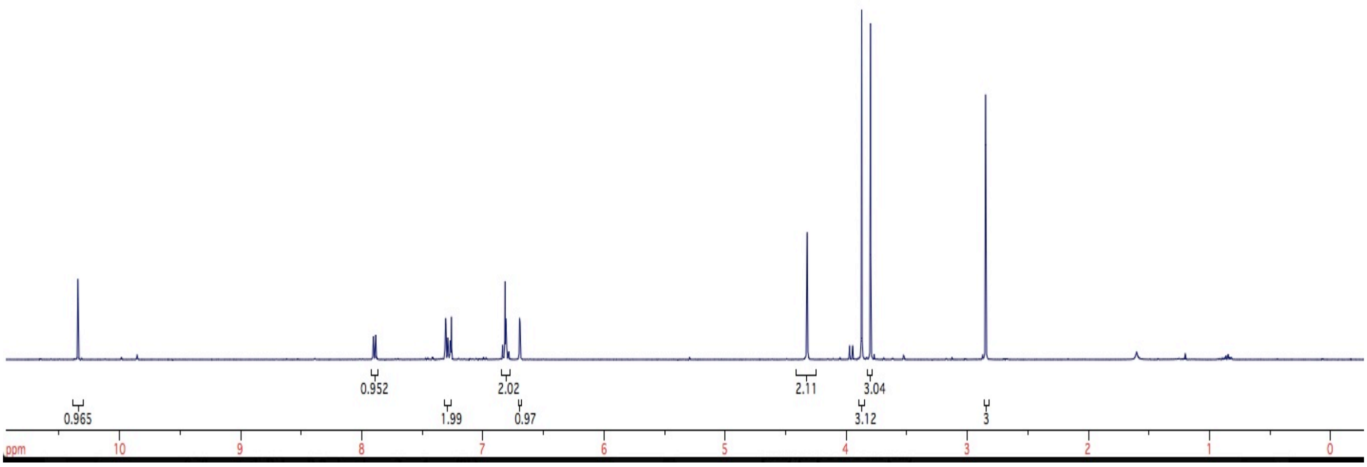

^{13}C NMR (100 MHz, $CDCl_3$)

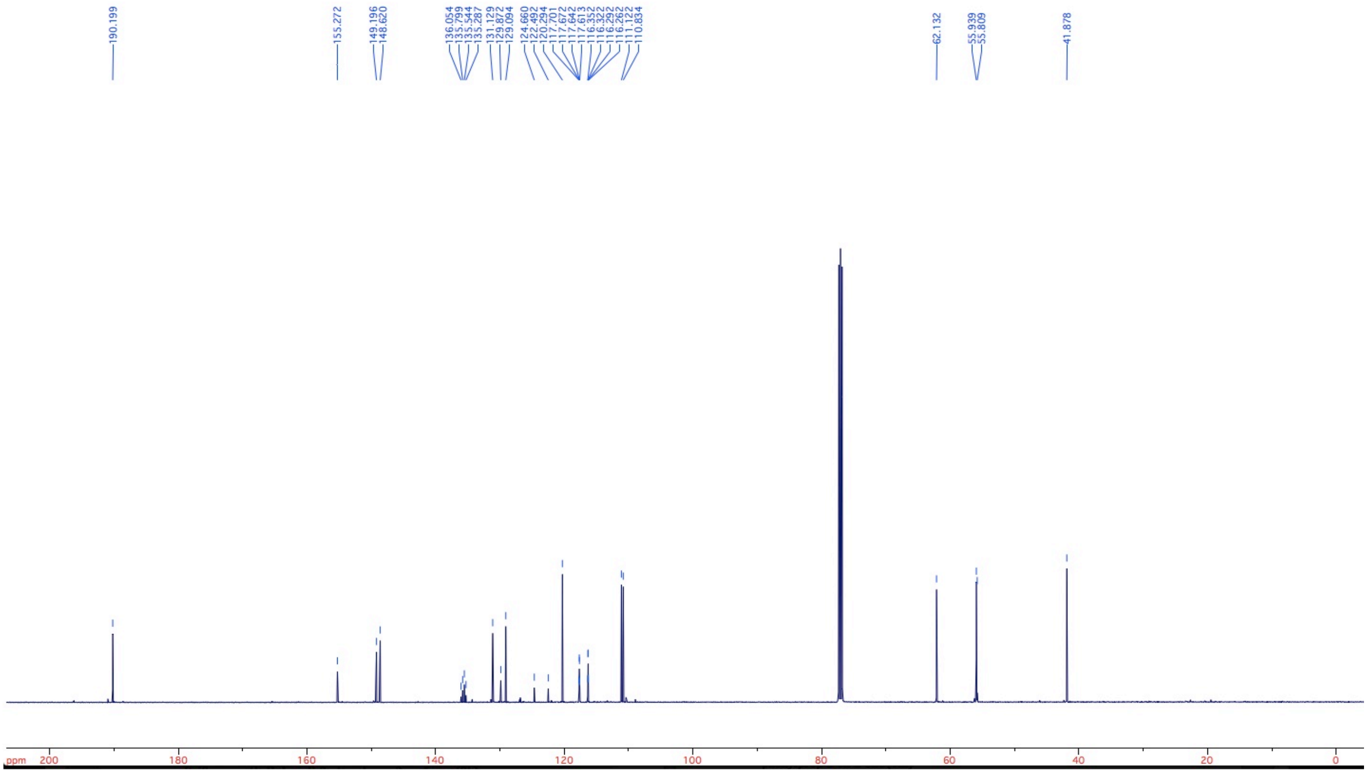

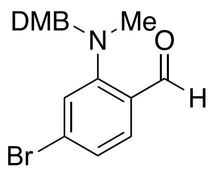

8k

^{1}H NMR (400 MHz, $CDCl_3$)

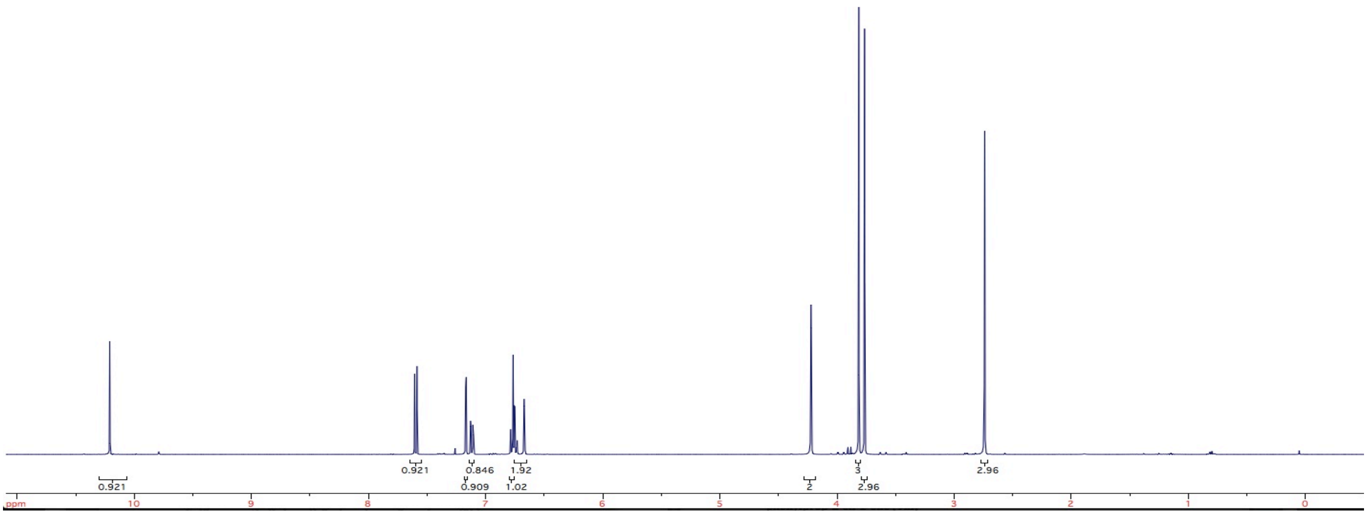

^{13}C NMR (100 MHz, $CDCl_3$)

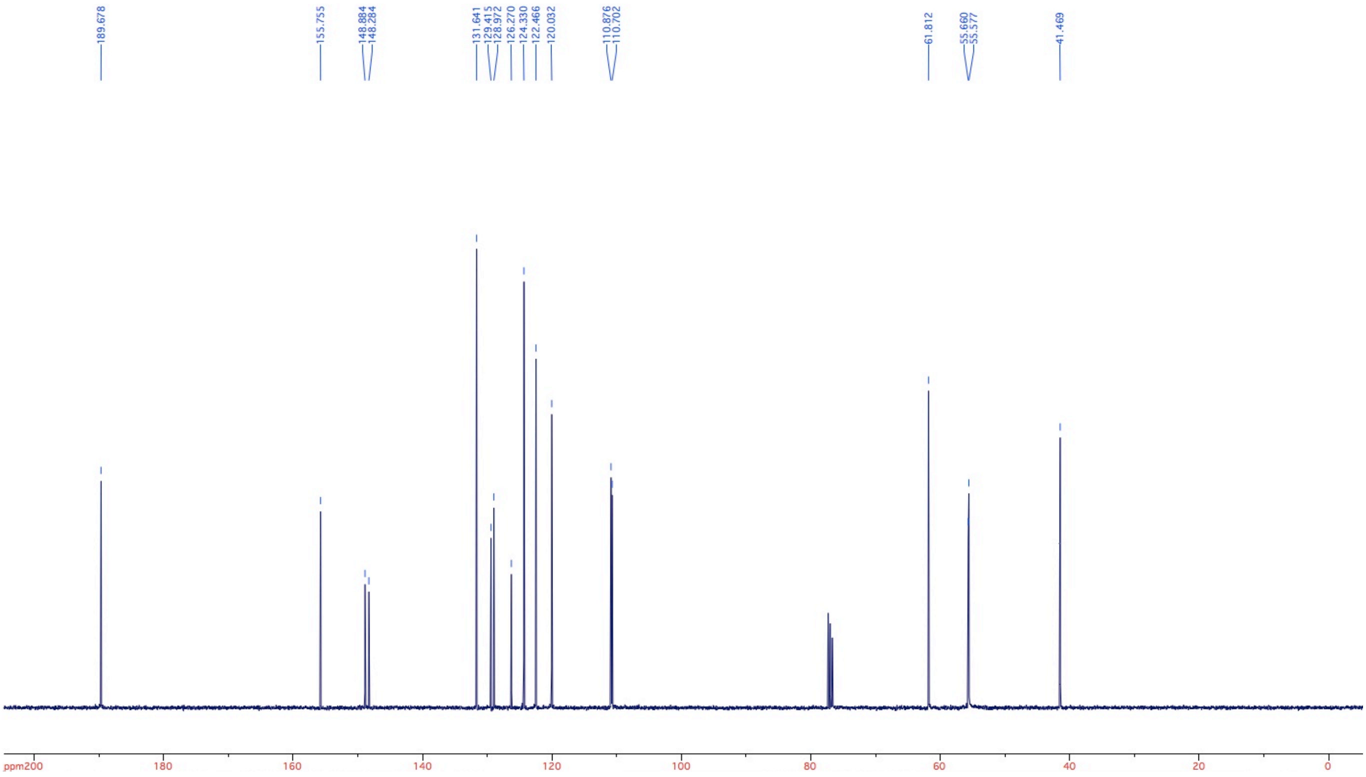

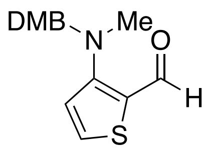

8l

^{1}H NMR (400 MHz, $CDCl_3$)

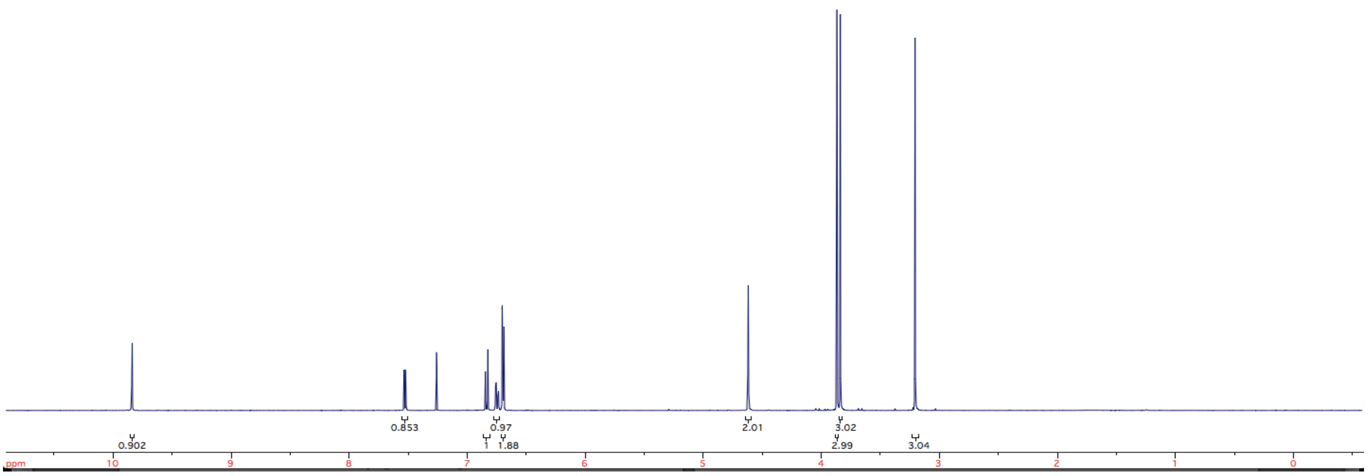

^{13}C NMR (100 MHz, $CDCl_3$)

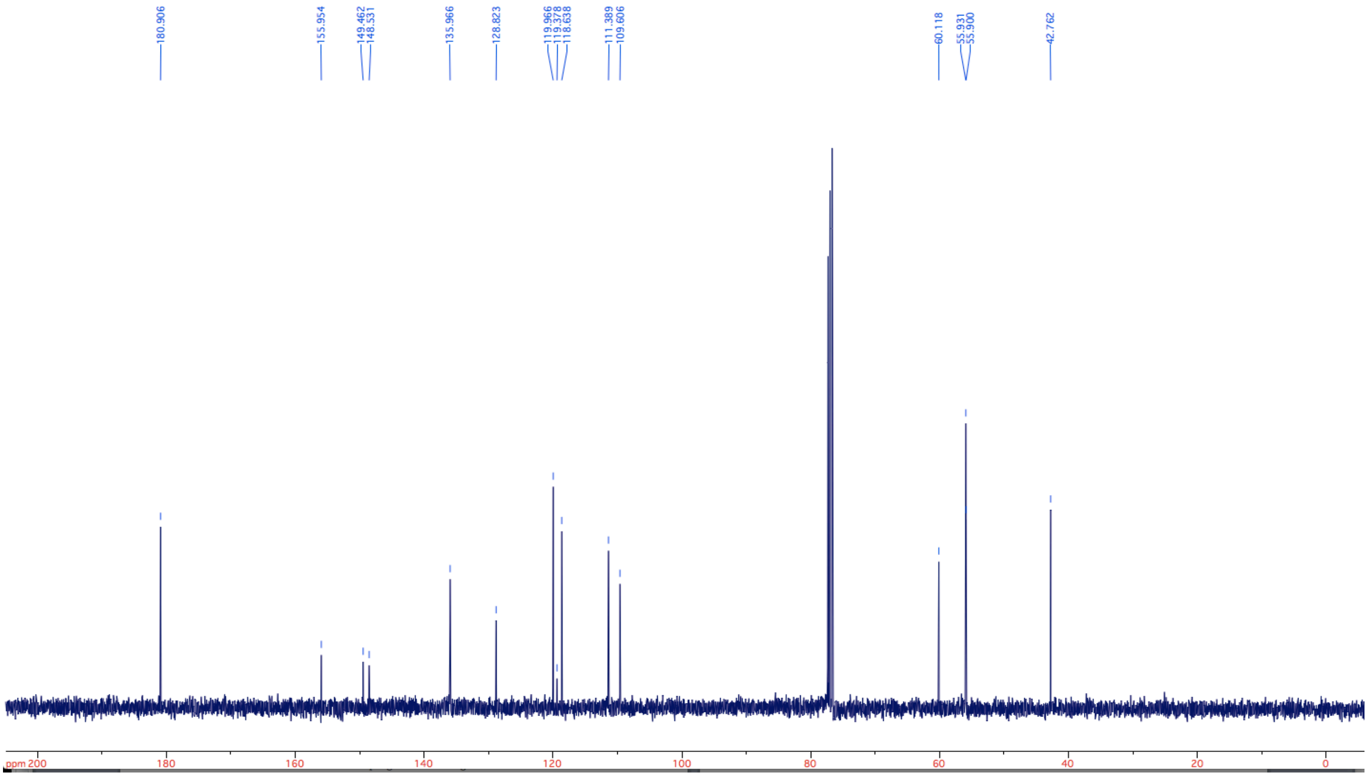

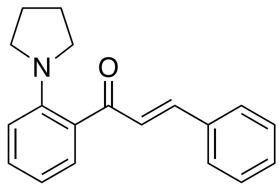

9b

^{1}H NMR (400 MHz, CDCl$_3$)

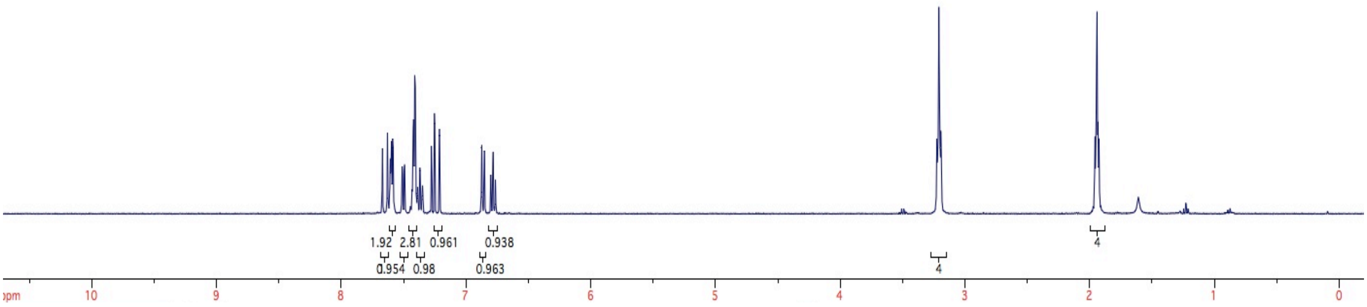

^{13}C NMR (100 MHz, CDCl$_3$)

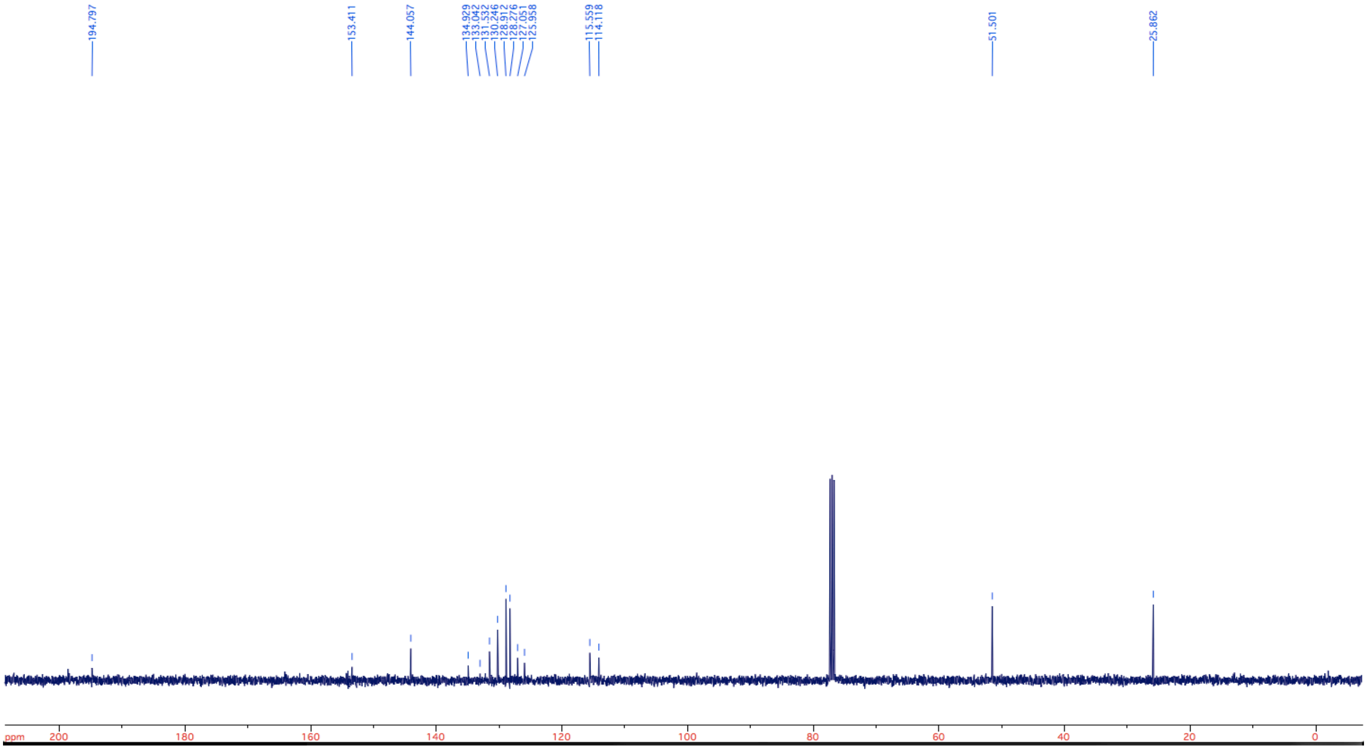

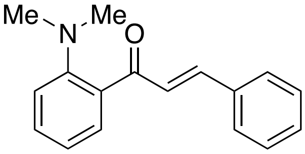

9c

^{1}H NMR (400 MHz, $CDCl_3$)

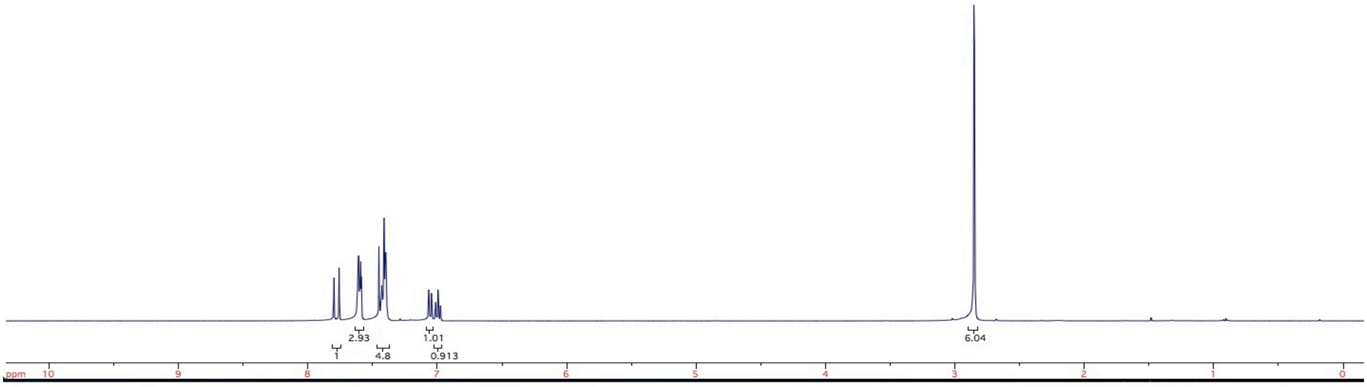

^{13}C NMR (100 MHz, $CDCl_3$)

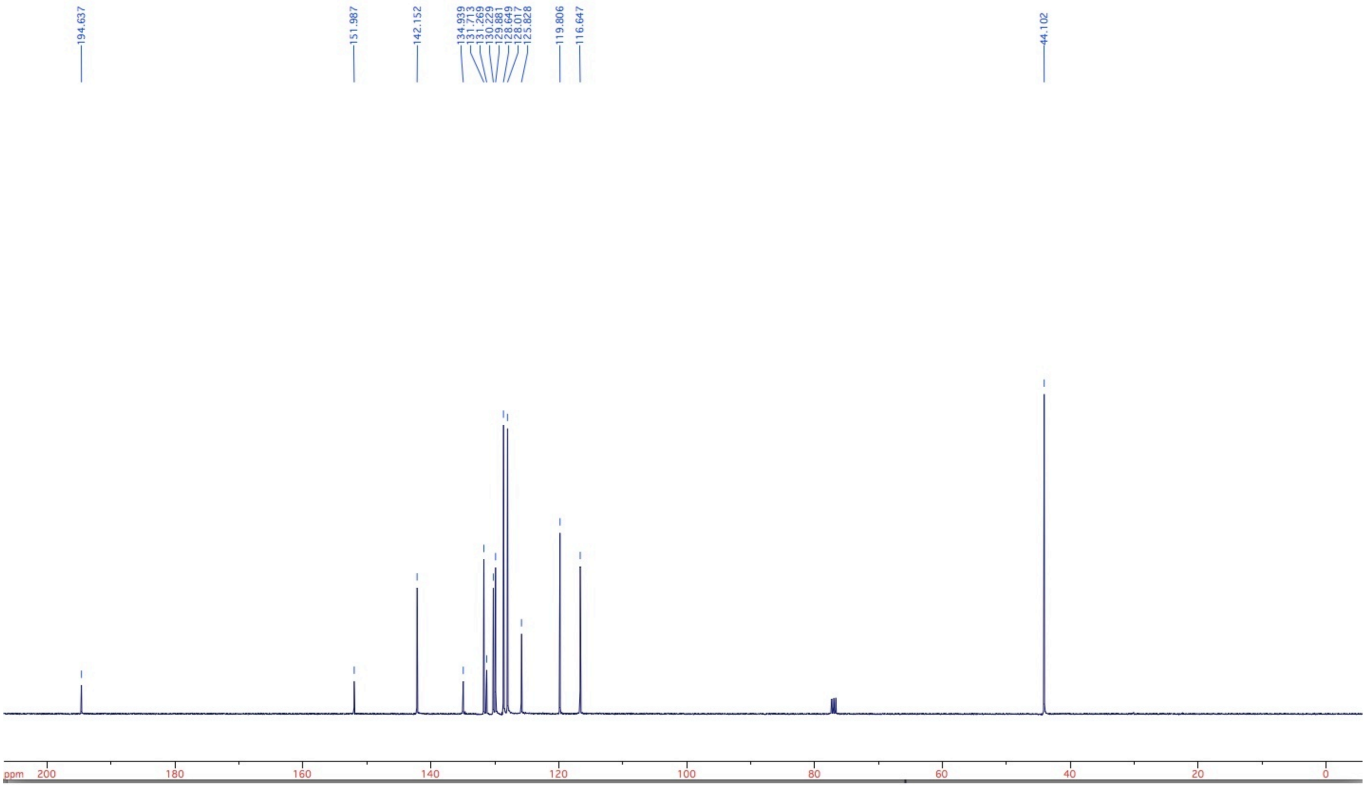

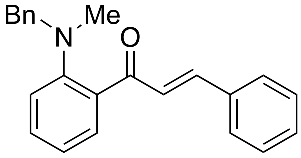

9d

^{1}H NMR (400 MHz, $CDCl_3$)

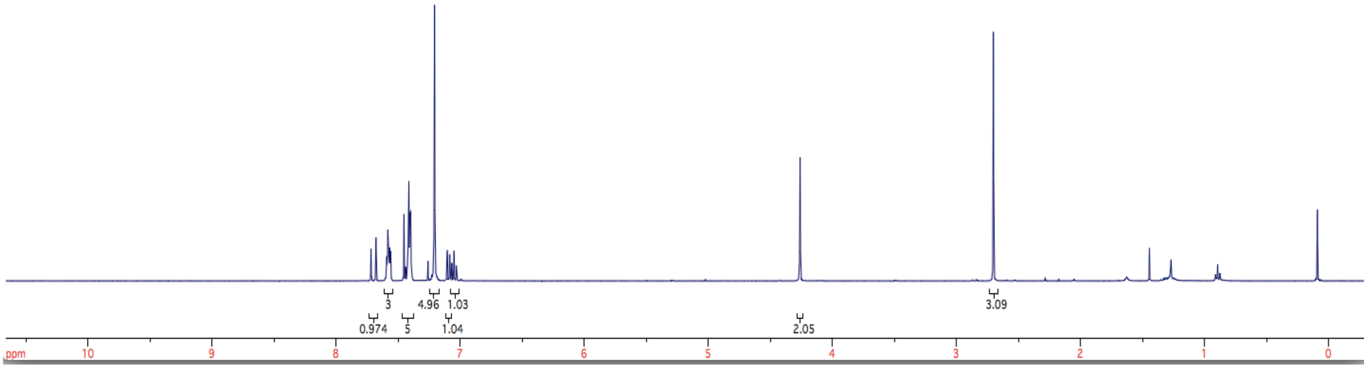

^{13}C NMR (100 MHz, $CDCl_3$)

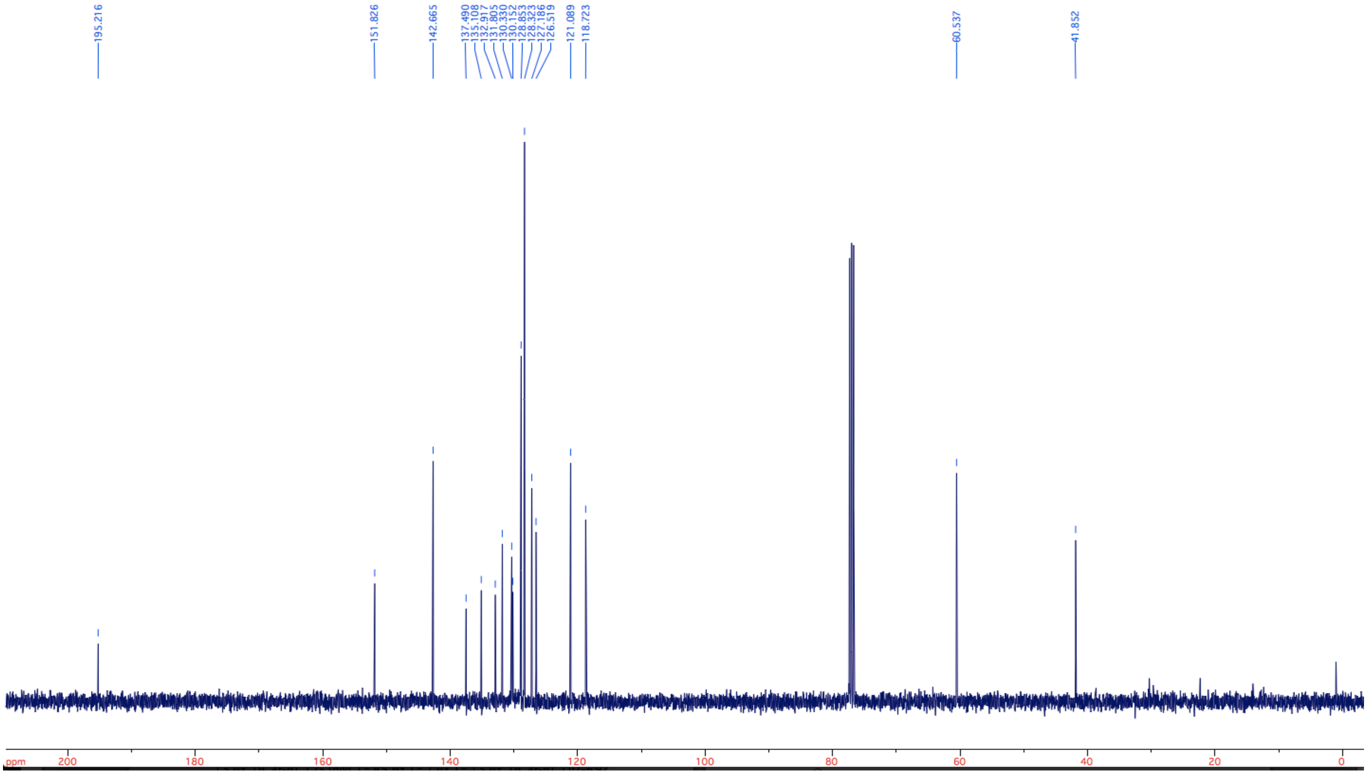

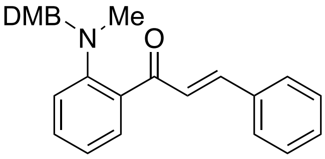

9e

^{1}H NMR (400 MHz, $CDCl_3$)

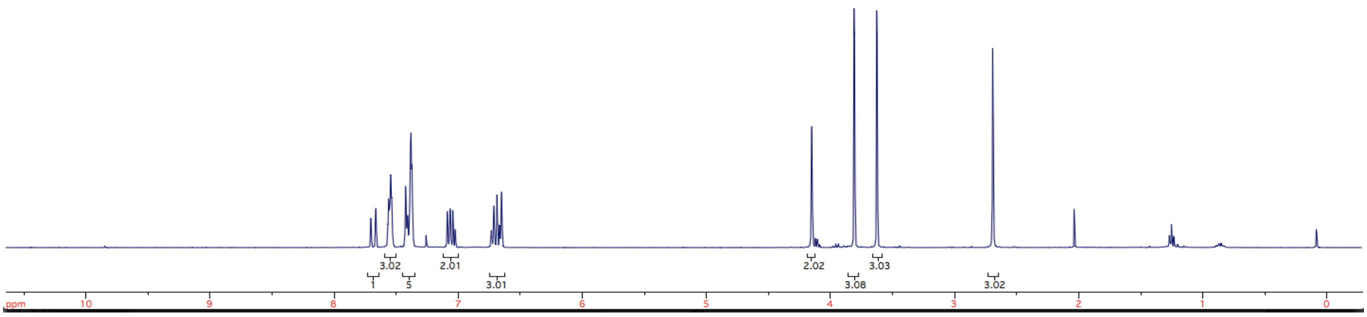

^{13}C NMR (100 MHz, $CDCl_3$)

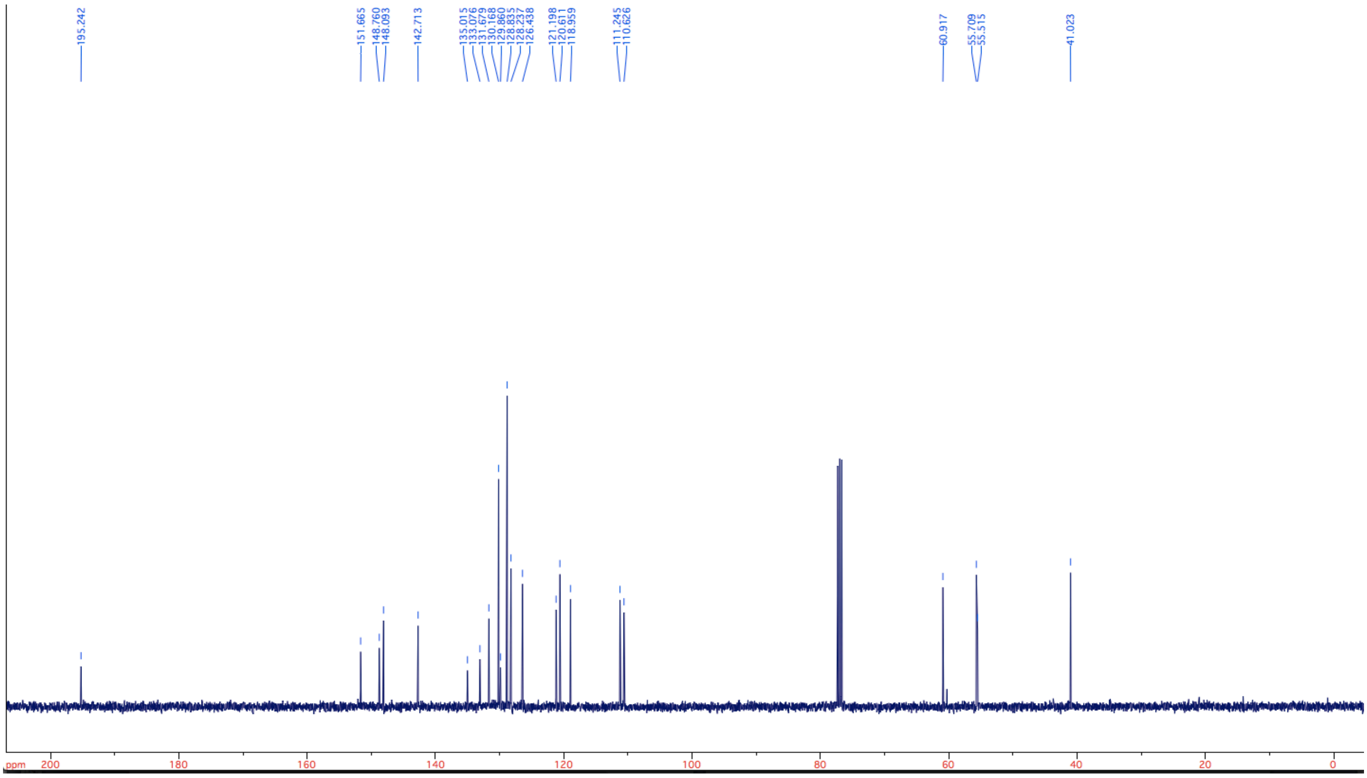

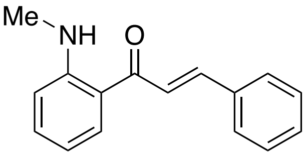

9f

^{1}H NMR (400 MHz, $CDCl_3$)

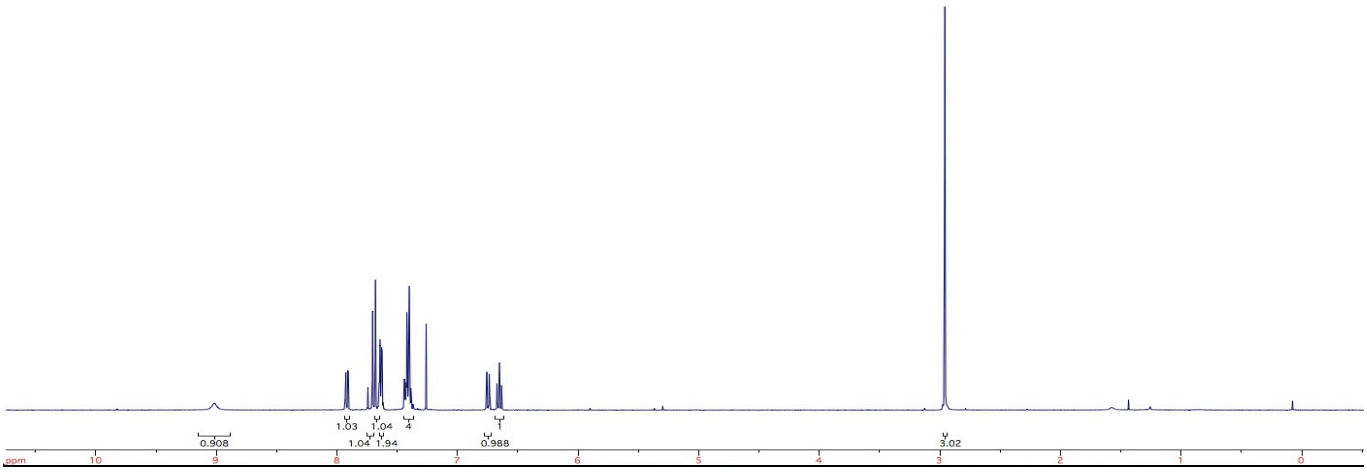

^{13}C NMR (100 MHz, $CDCl_3$)

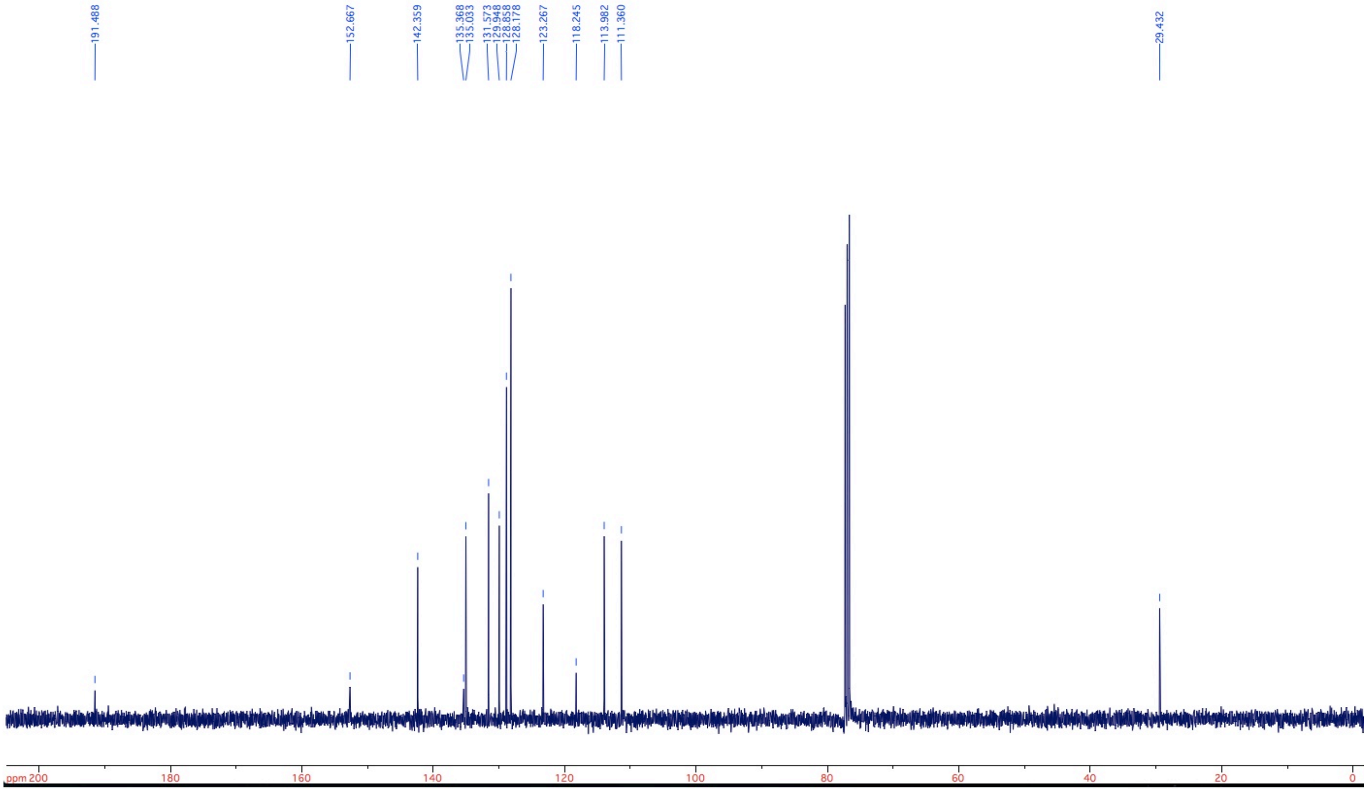

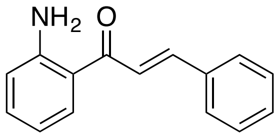

9g

^{1}H NMR (400 MHz, $CDCl_3$)

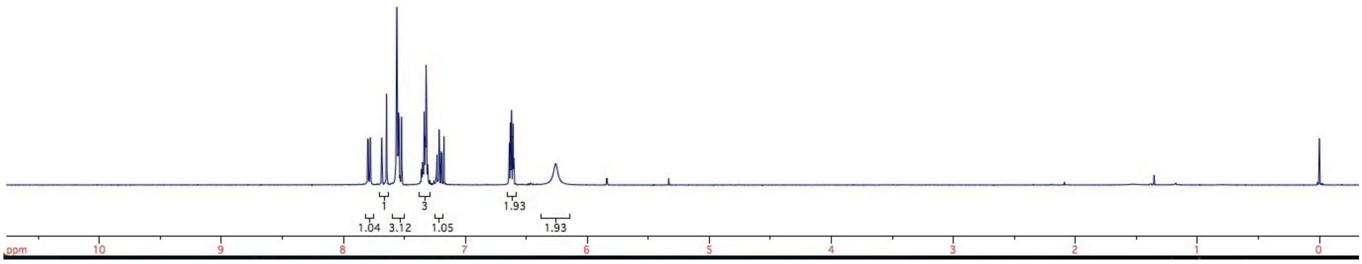

^{13}C NMR (100 MHz, $CDCl_3$)

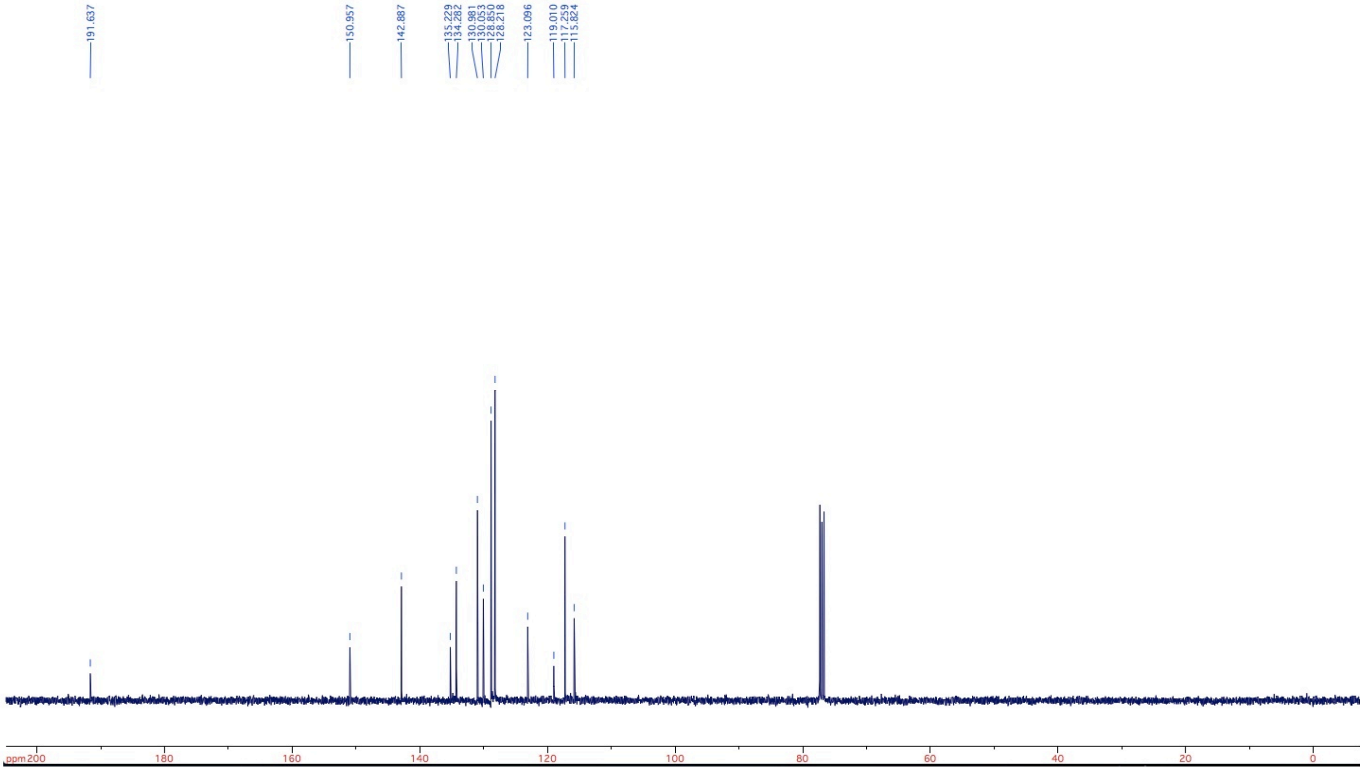

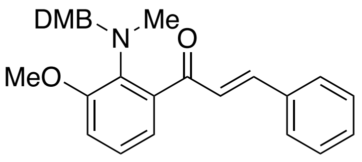

9h

^{1}H NMR (400 MHz, $CDCl_3$)

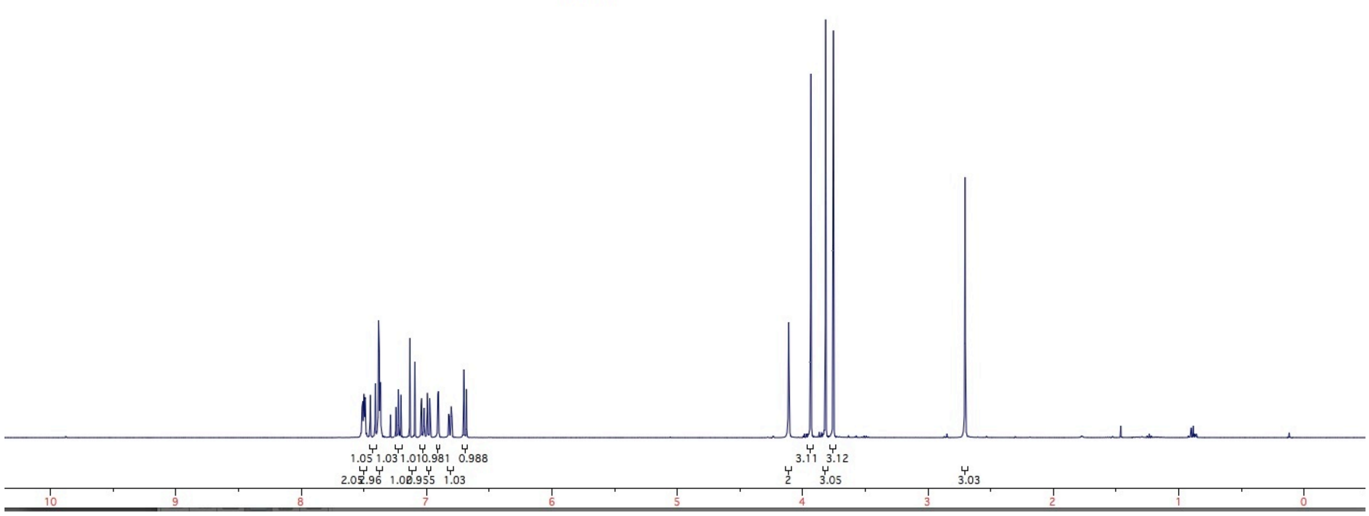

^{13}C NMR (100 MHz, $CDCl_3$)

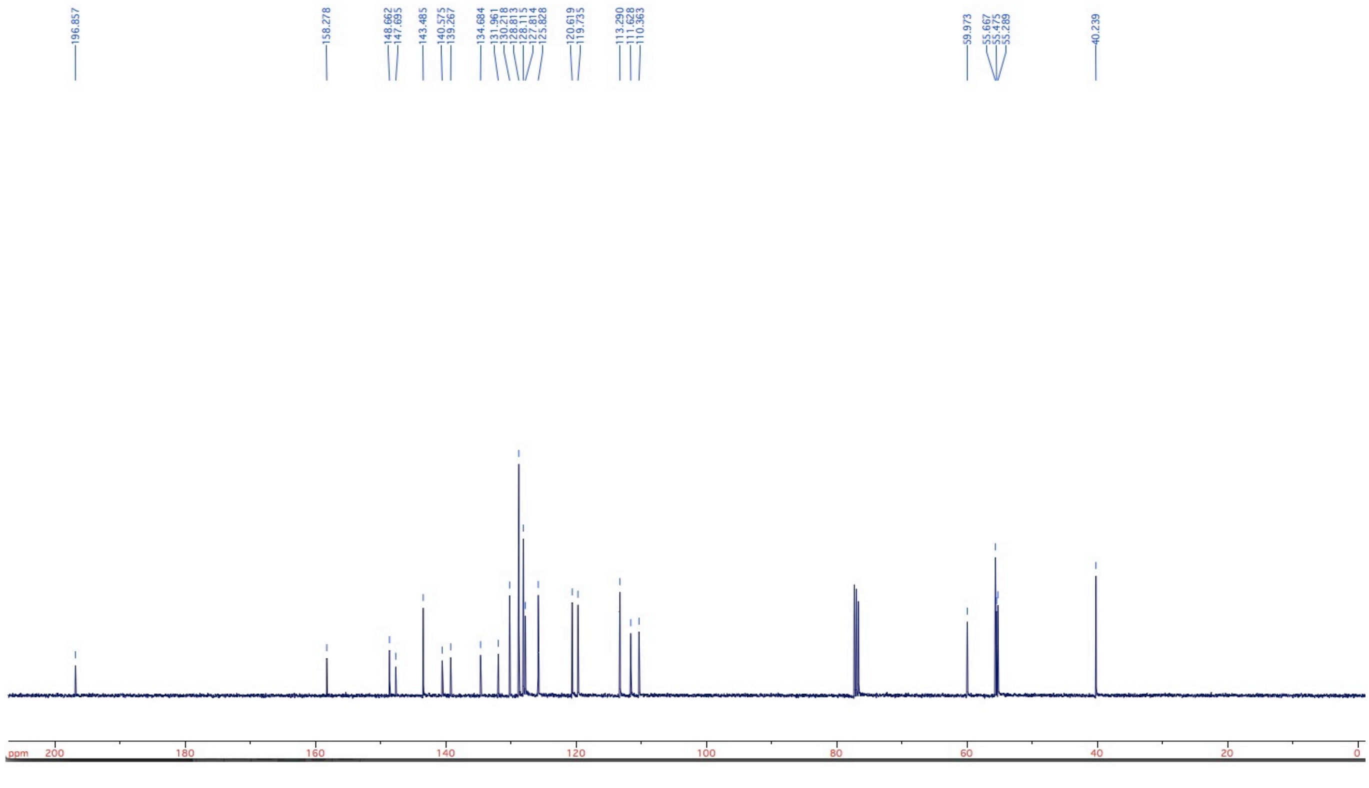

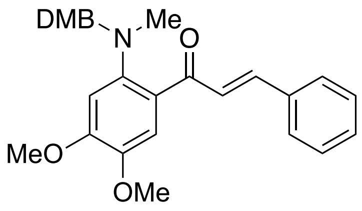

9i

^{1}H NMR (400 MHz, $CDCl_3$)

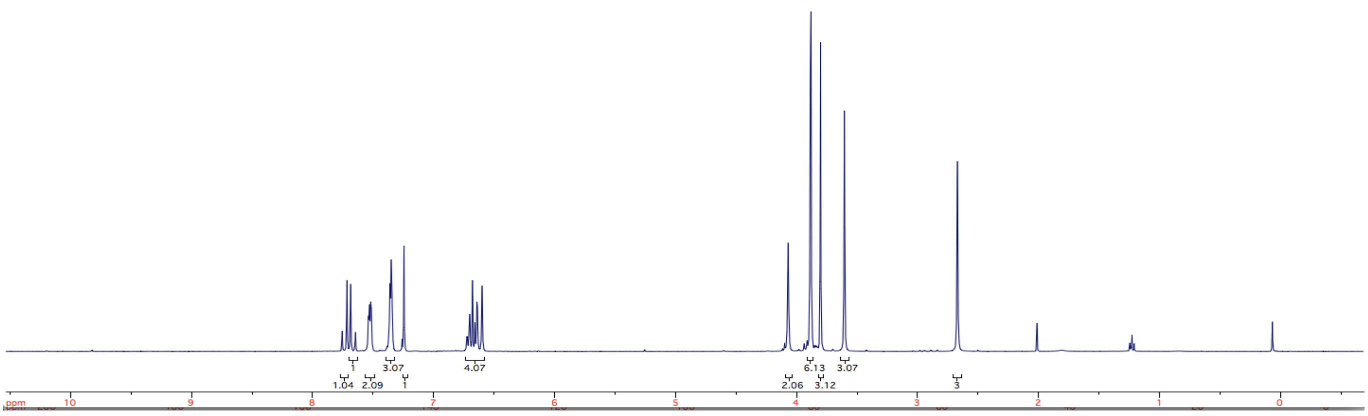

^{13}C NMR (100 MHz, $CDCl_3$)

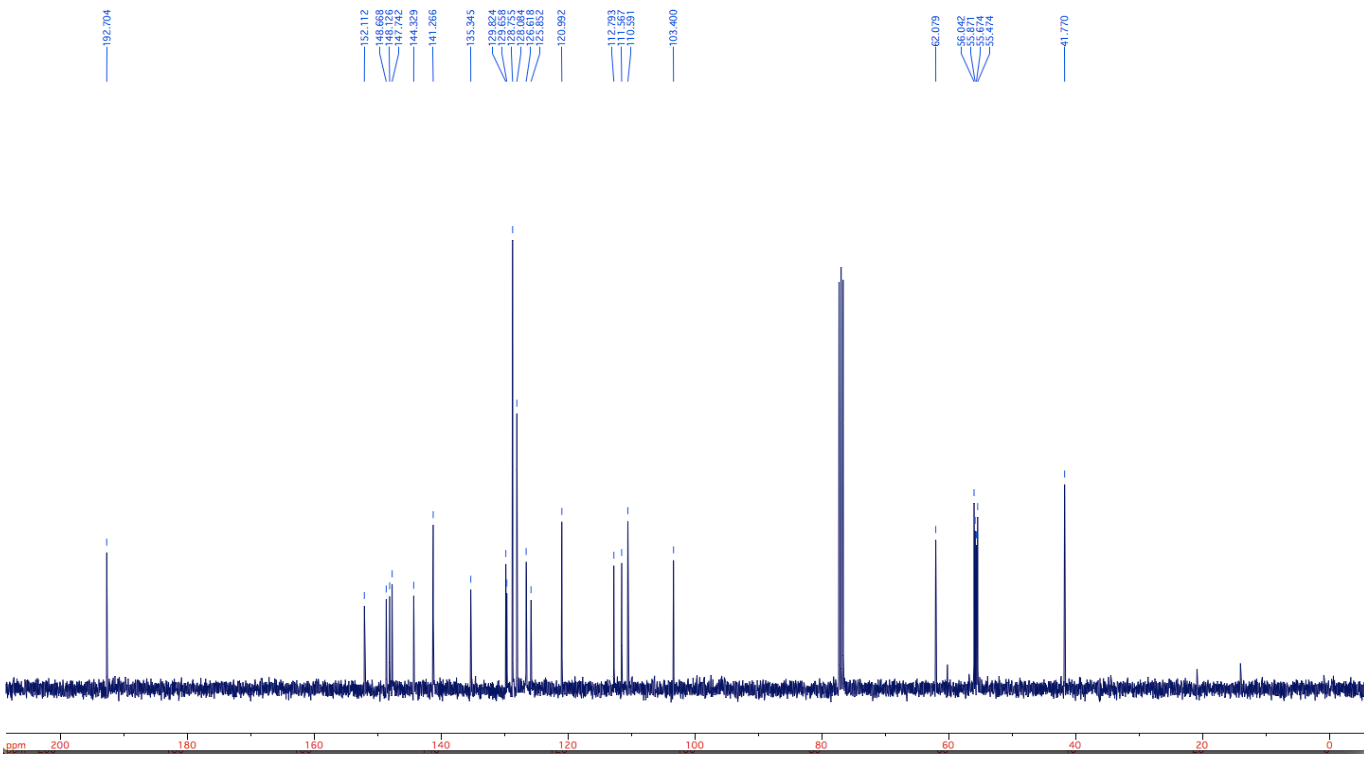

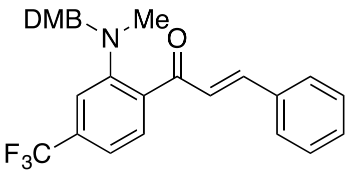

9k

^{1}H NMR (400 MHz, $CDCl_3$)

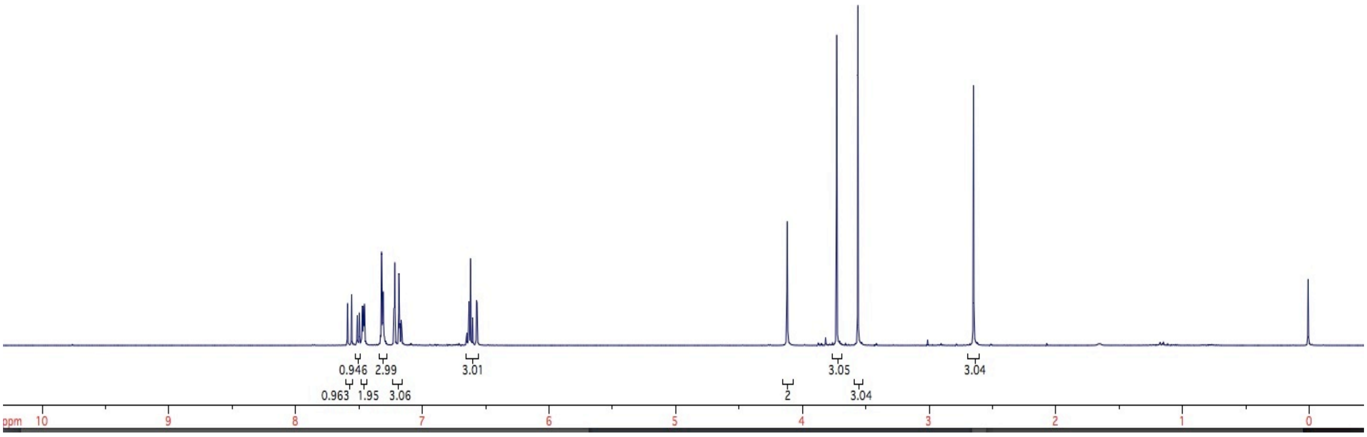

^{13}C NMR (100 MHz, $CDCl_3$)

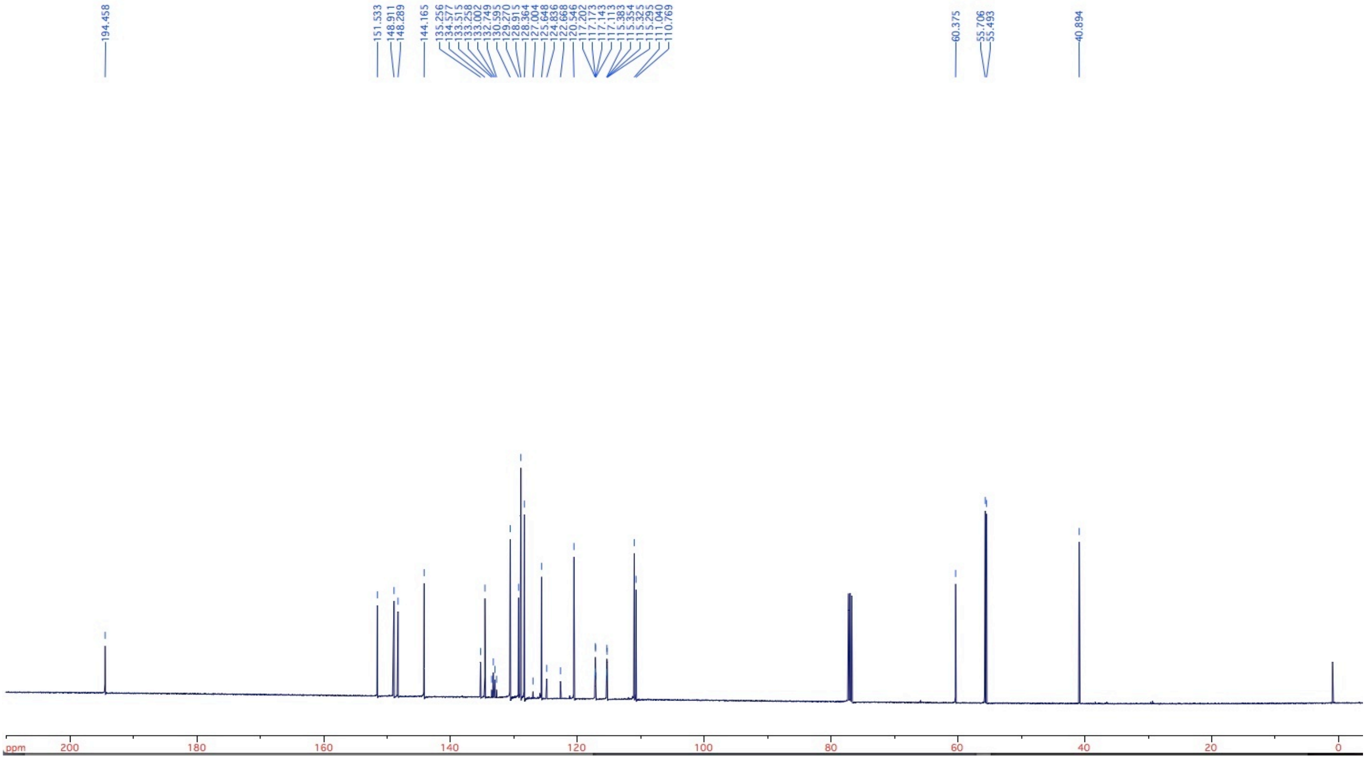

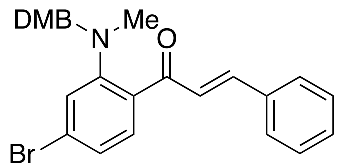

9l

^{1}H NMR (400 MHz, $CDCl_3$)

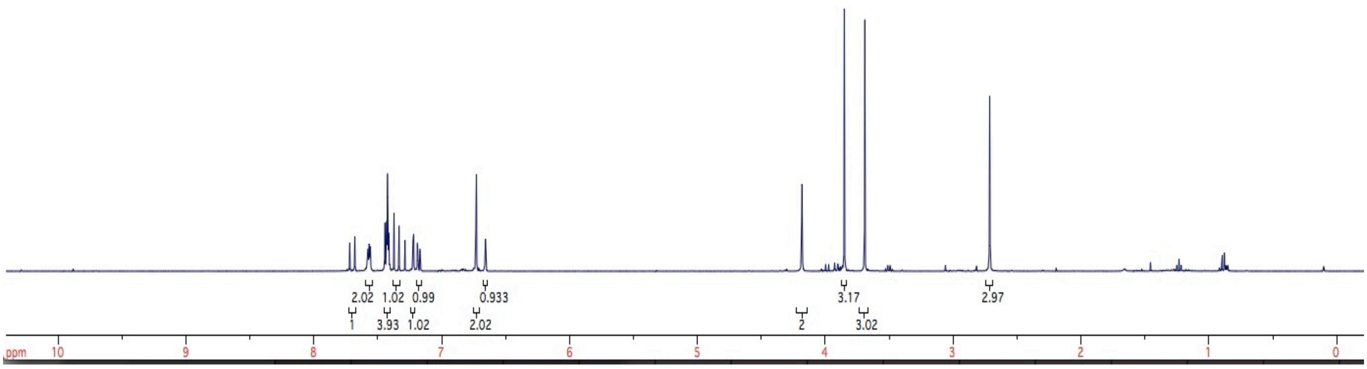

^{13}C NMR (100 MHz, $CDCl_3$)

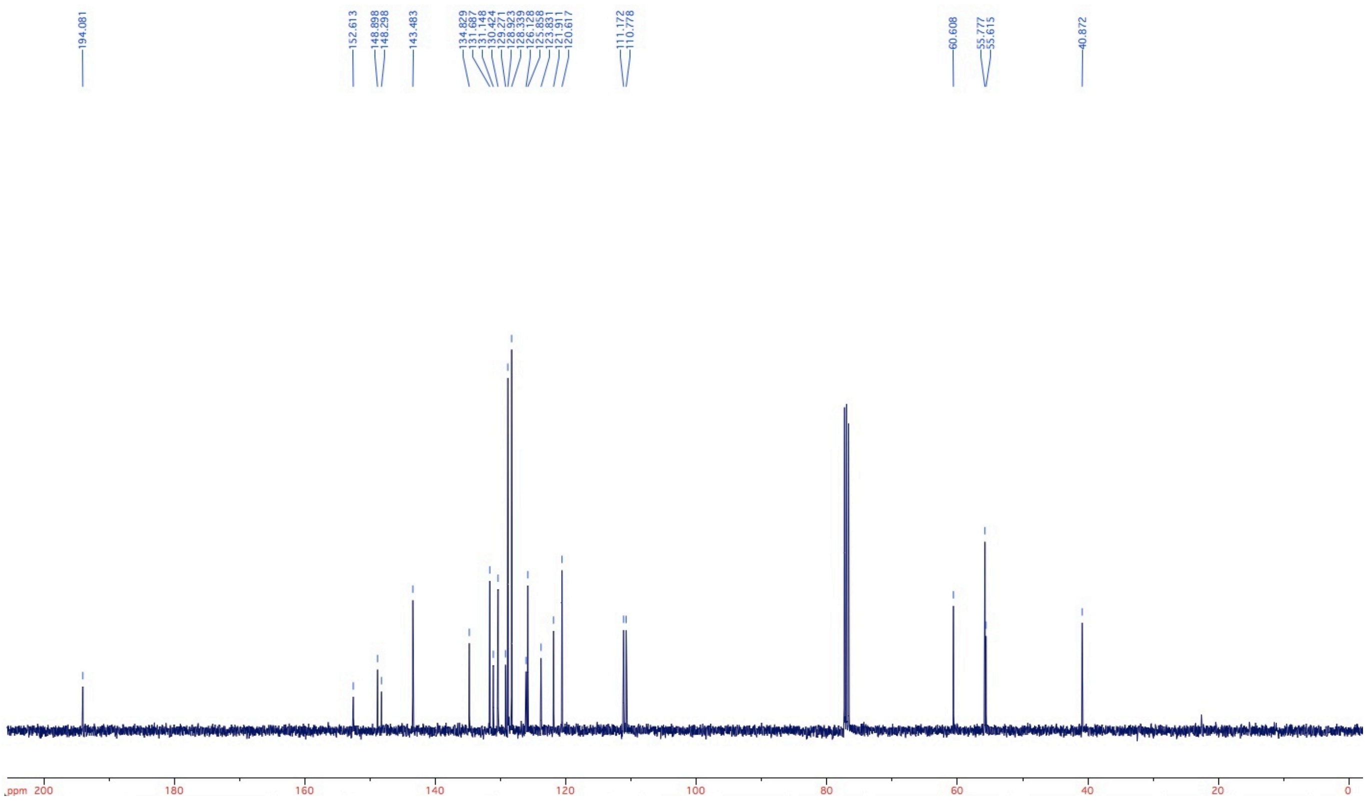

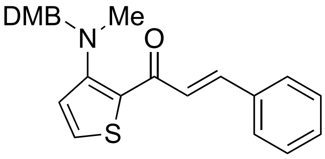

9m

^{1}H NMR (400 MHz, $CDCl_3$)

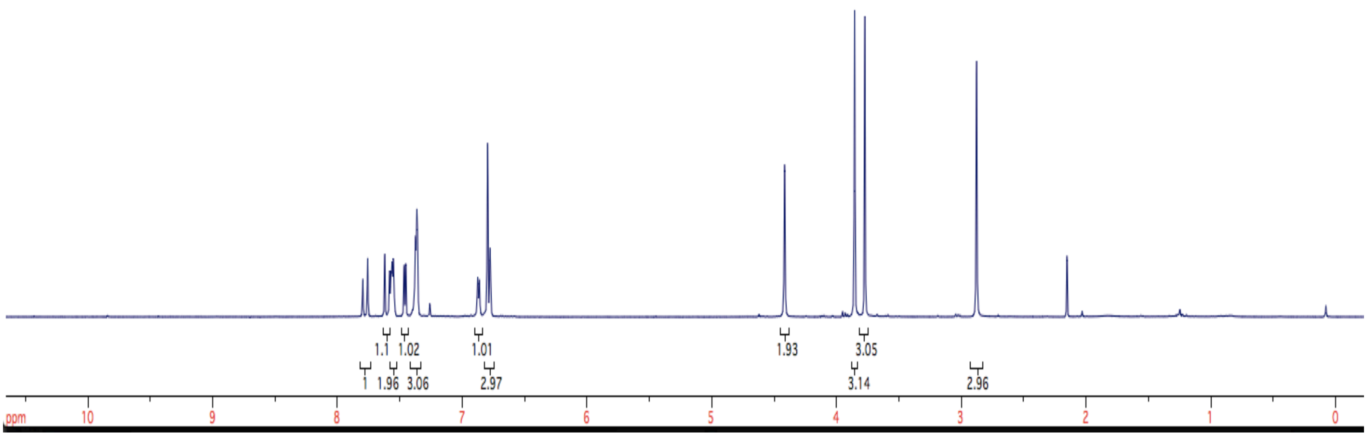

^{13}C NMR (100 MHz, $CDCl_3$)

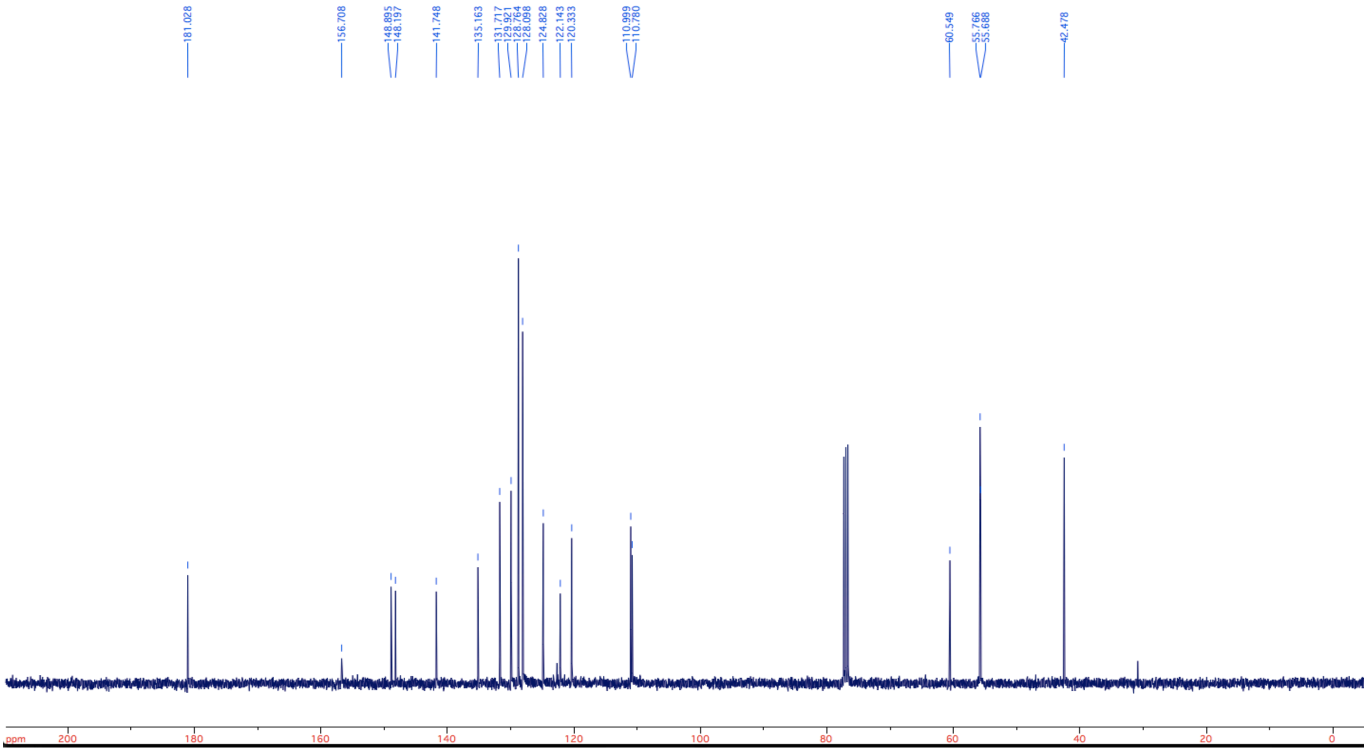

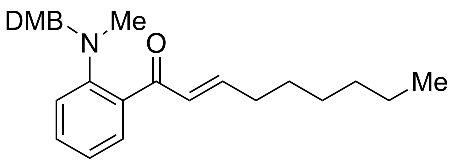

9n

^{1}H NMR (400 MHz, $CDCl_3$)

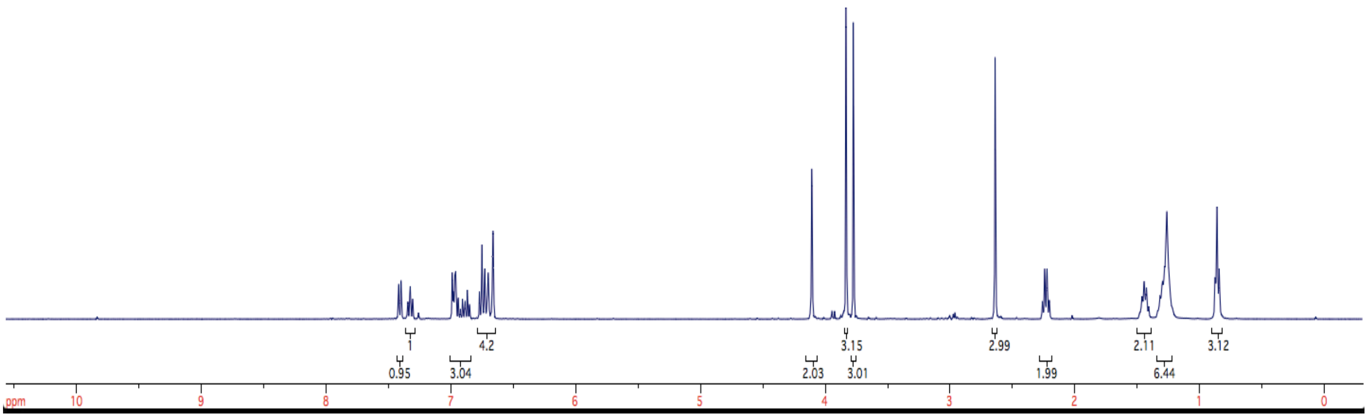

^{13}C NMR (100 MHz, $CDCl_3$)

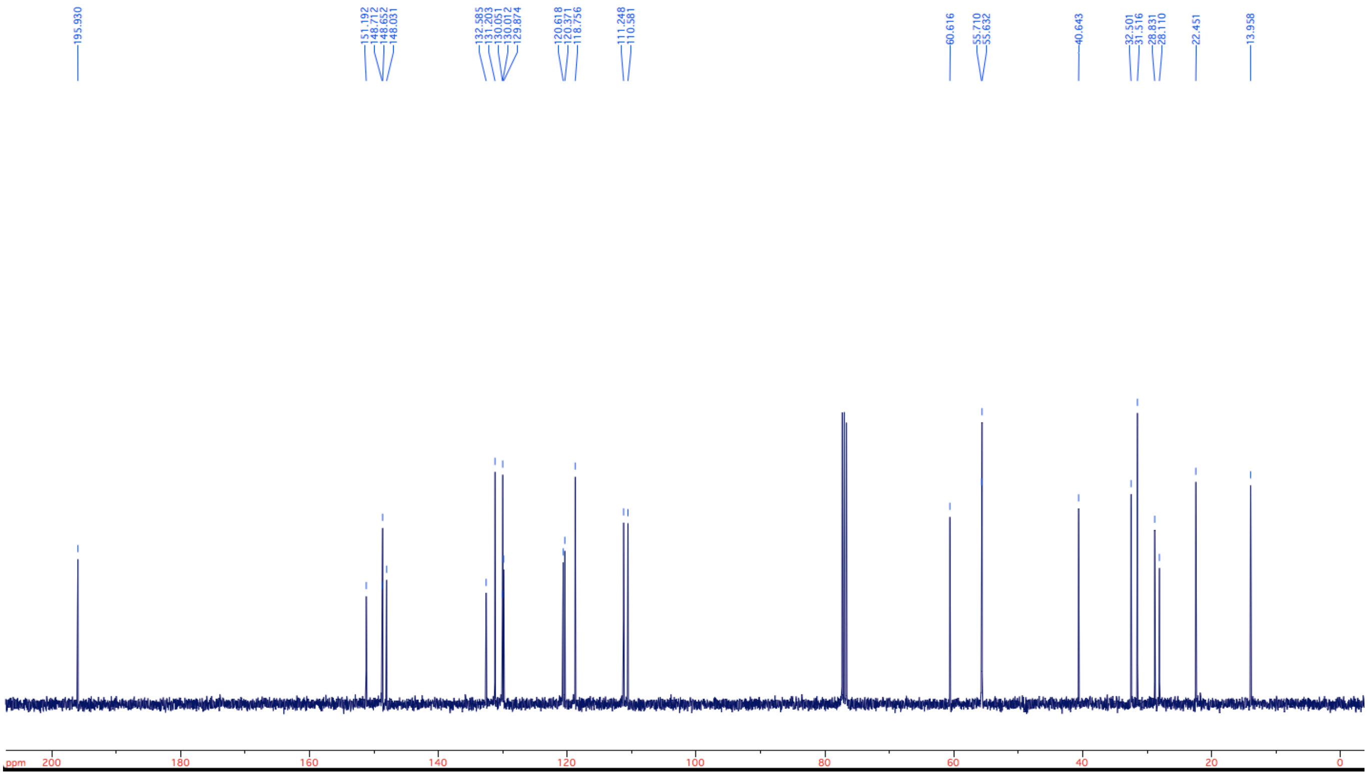

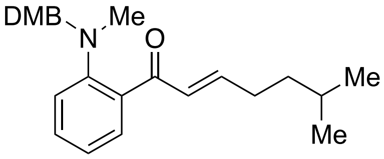

9o

^{1}H NMR (400 MHz, $CDCl_3$)

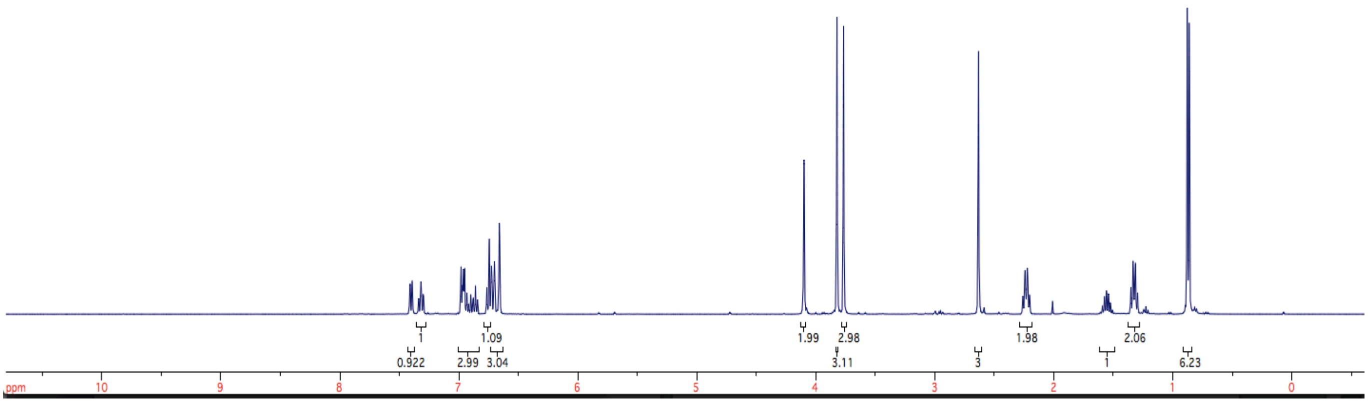

^{13}C NMR (100 MHz, $CDCl_3$)

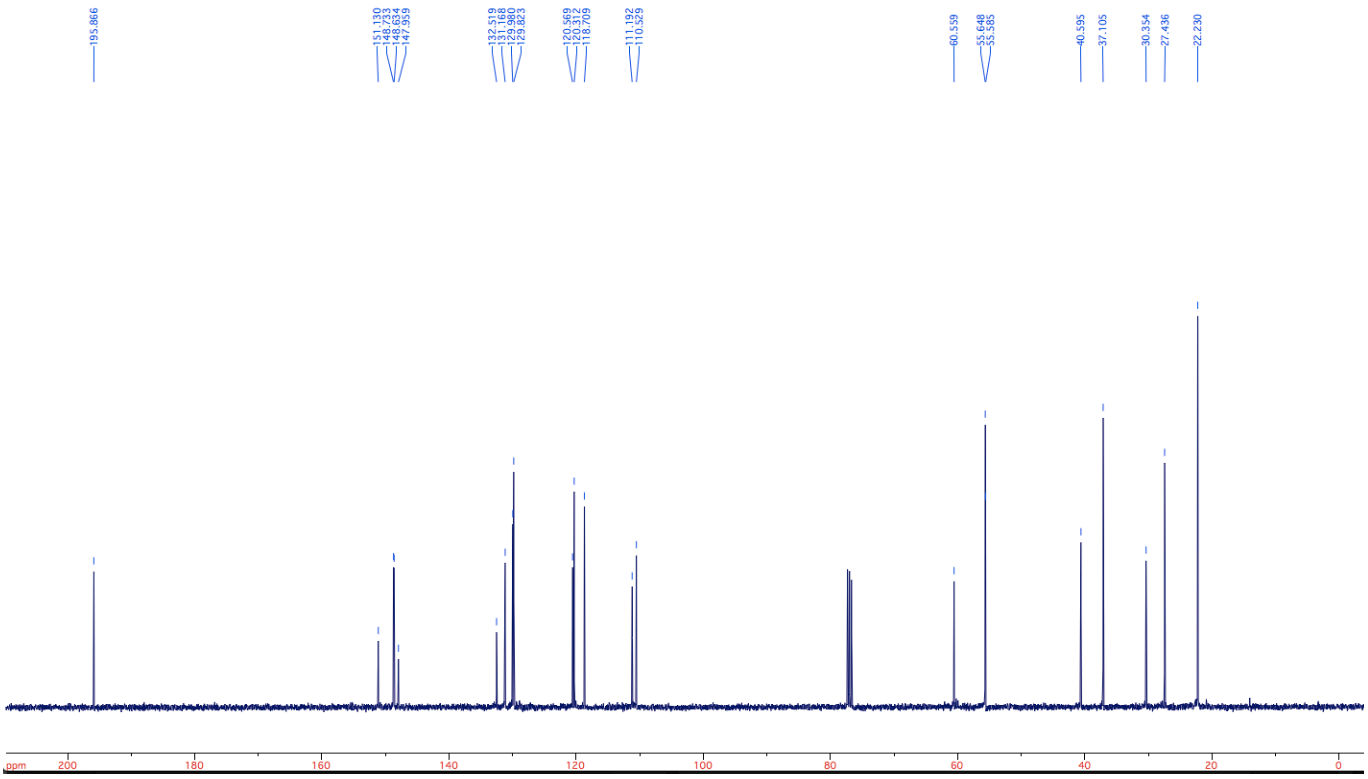

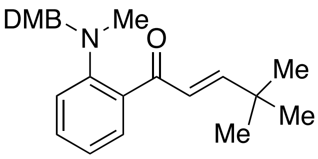

9p

^{1}H NMR (400 MHz, $CDCl_3$)

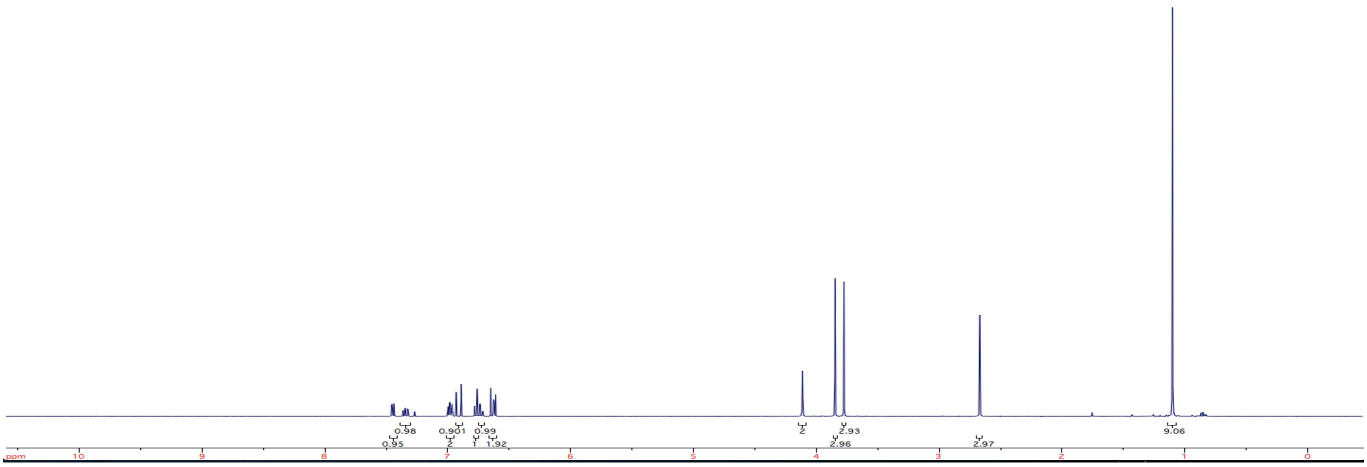

^{13}C NMR (100 MHz, $CDCl_3$)

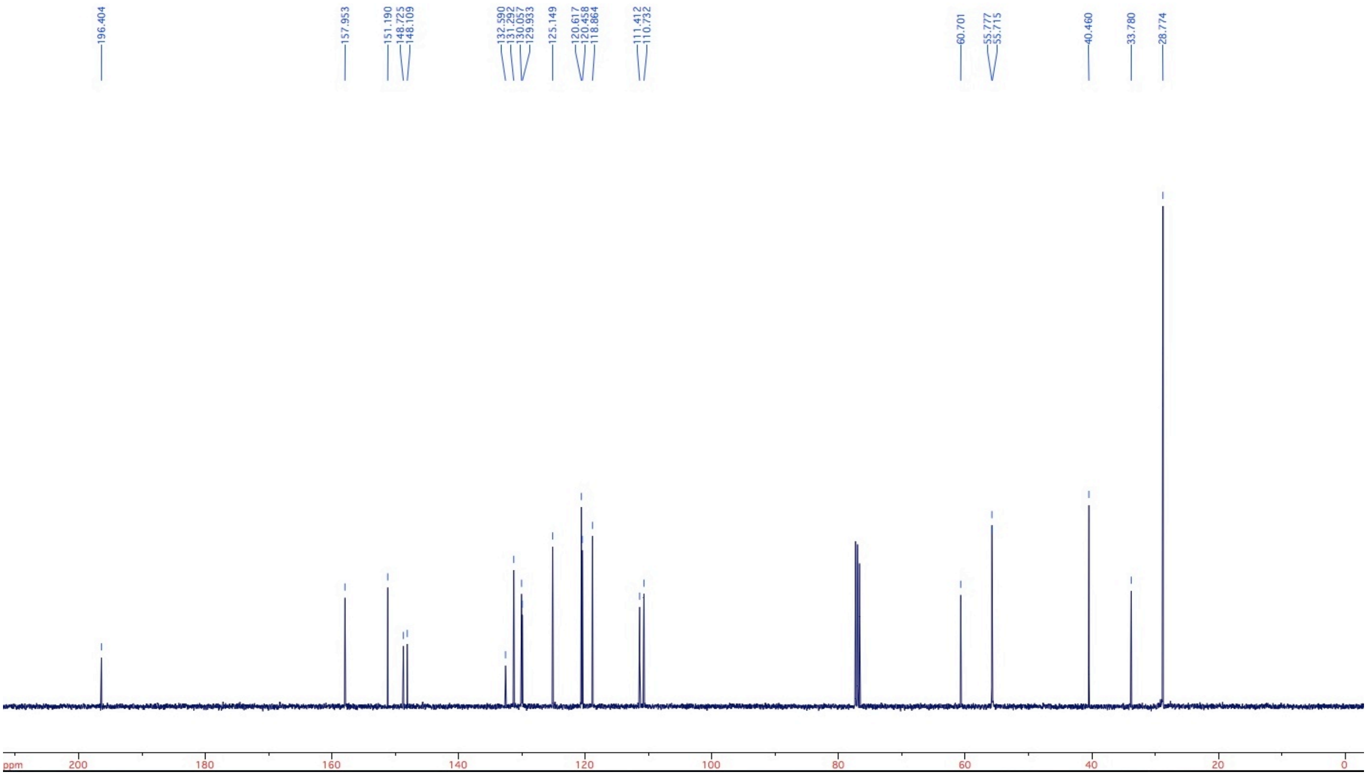

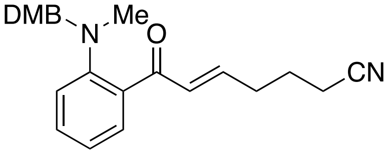

9r

^{1}H NMR (400 MHz, $CDCl_3$)

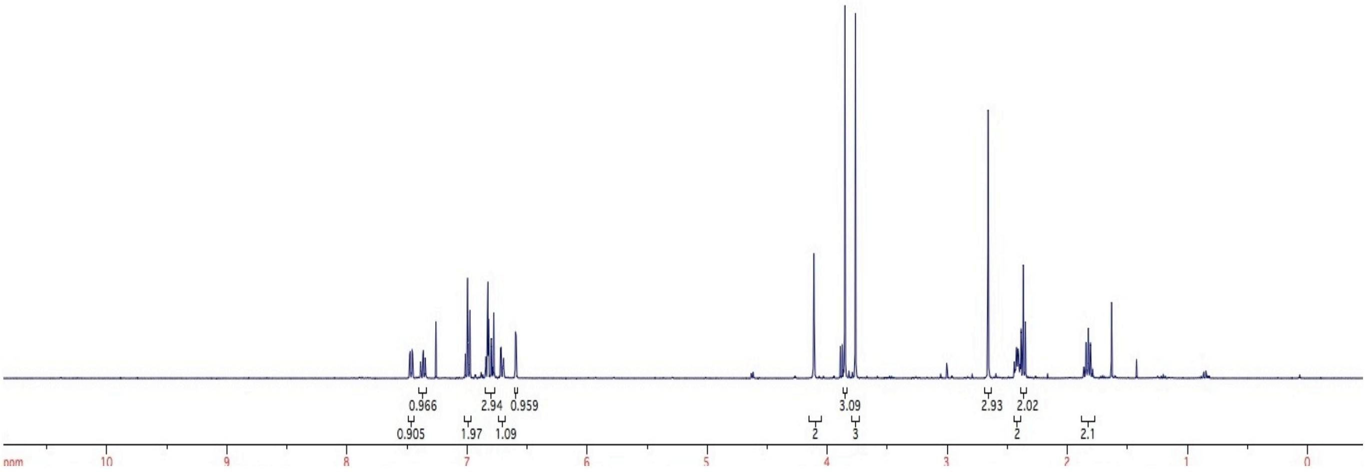

^{13}C NMR (100 MHz, $CDCl_3$)

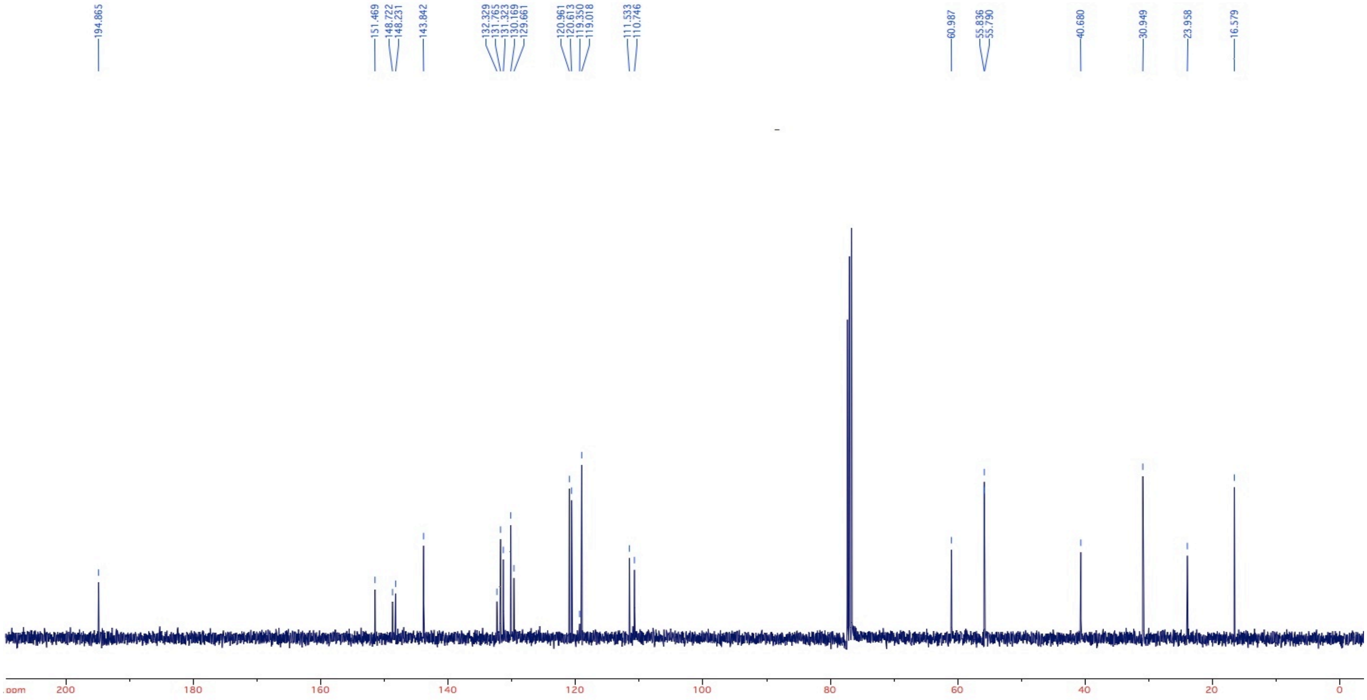

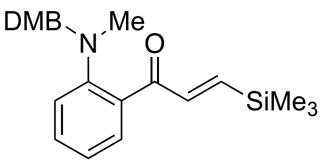

9s

^{1}H NMR (400 MHz, $CDCl_3$)

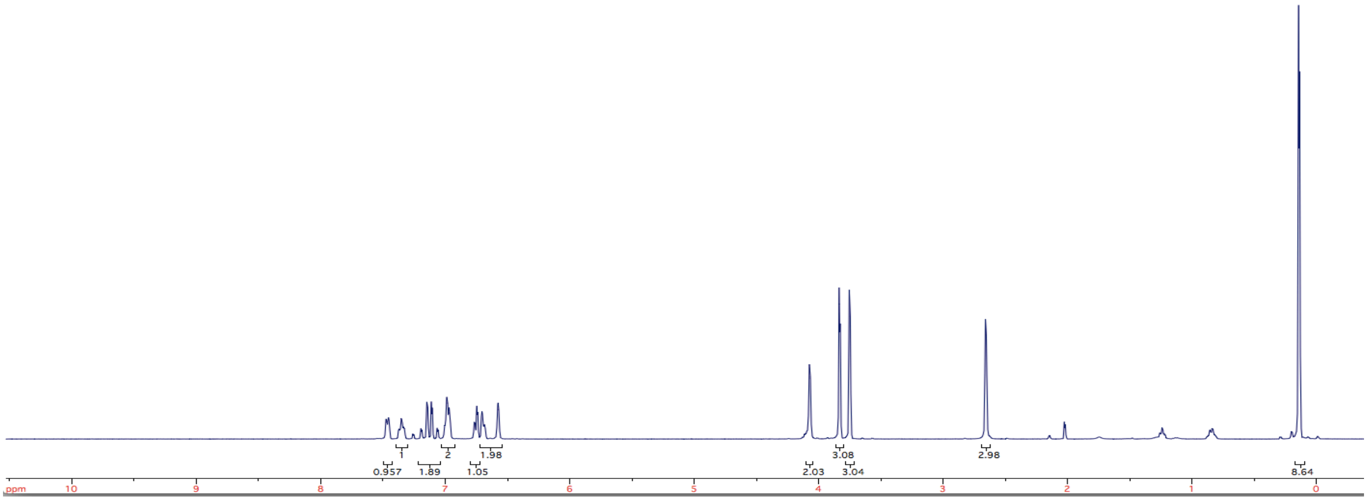

^{13}C NMR (100 MHz, $CDCl_3$)

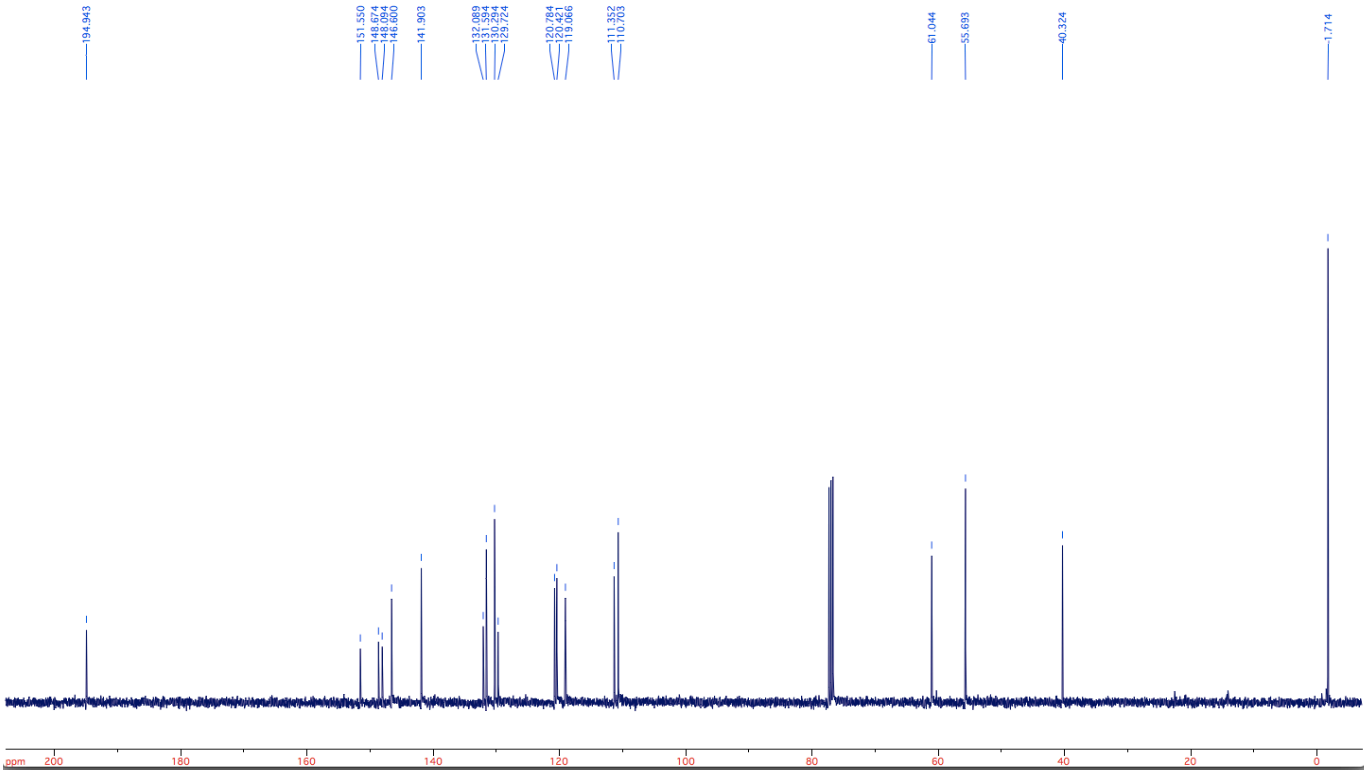

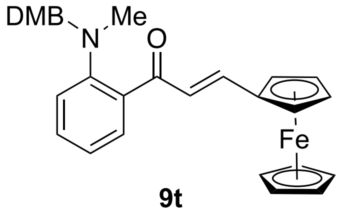

9t

^{1}H NMR (400 MHz, $CDCl_3$)

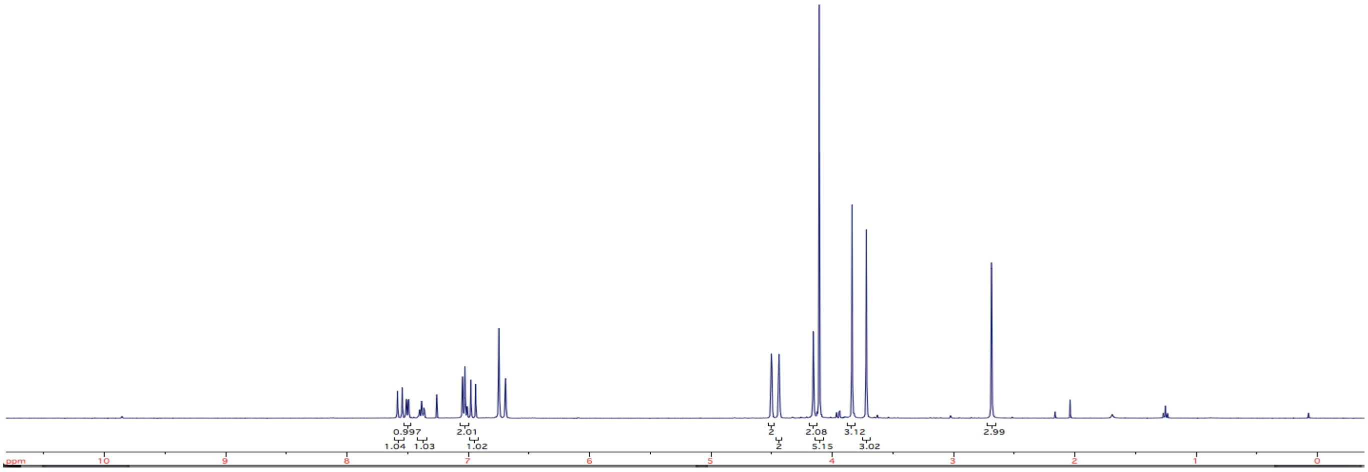

^{13}C NMR (100 MHz, $CDCl_3$)

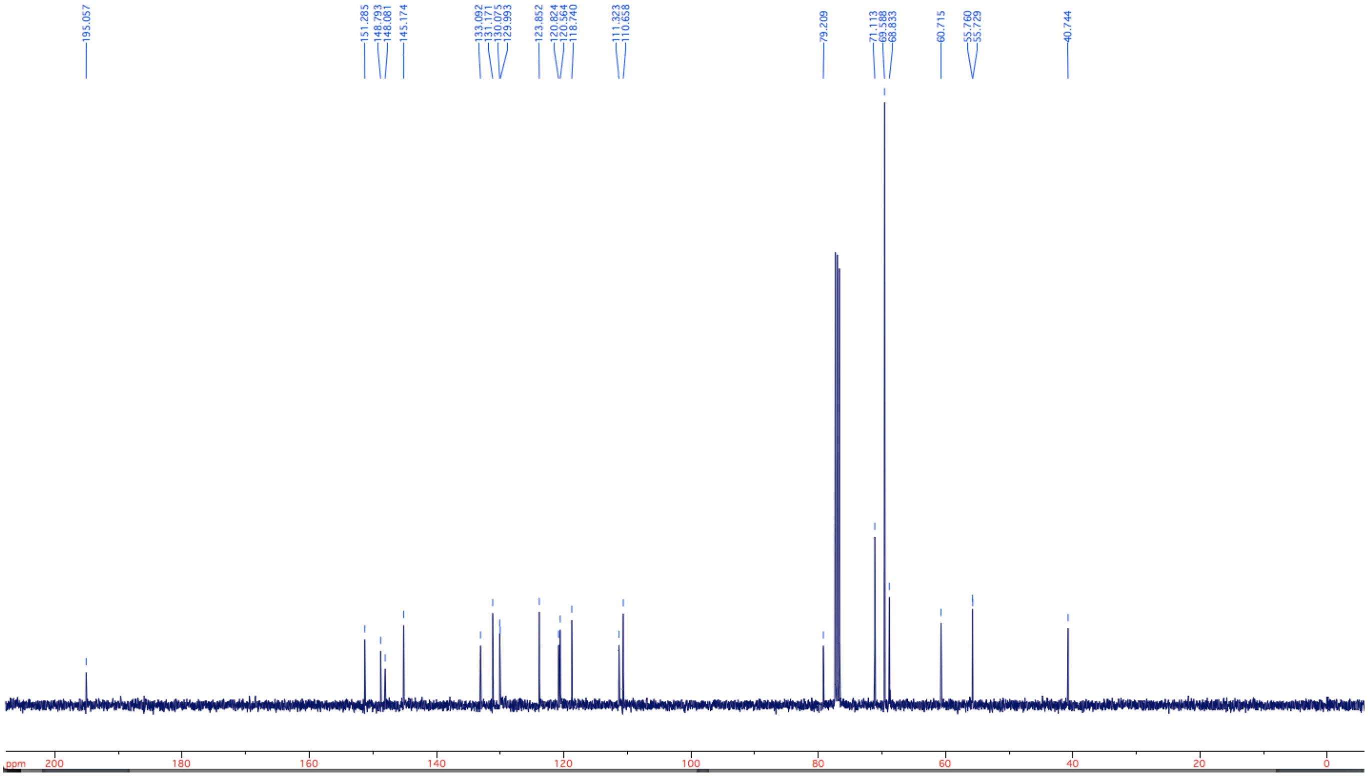

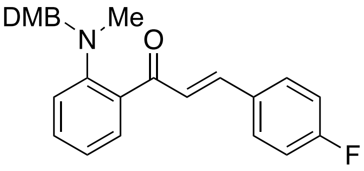

9u

^{1}H NMR (400 MHz, $CDCl_3$)

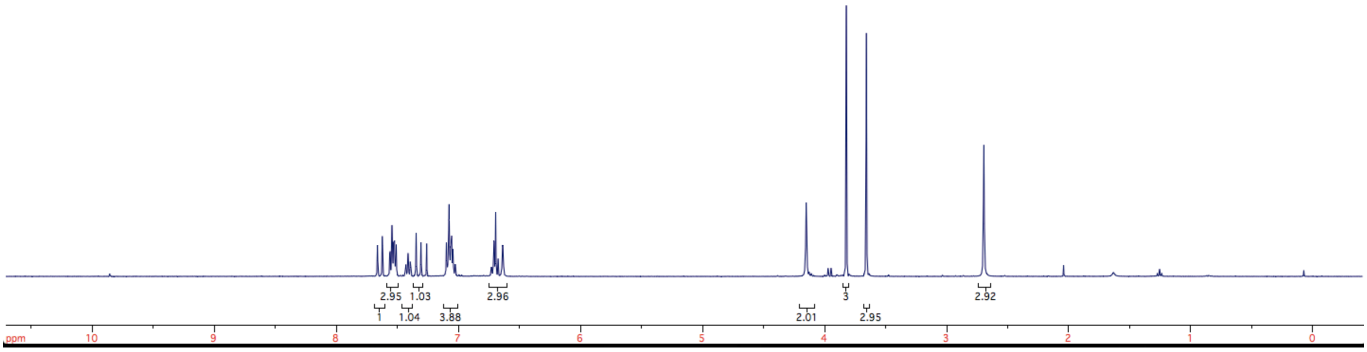

^{13}C NMR (100 MHz, $CDCl_3$)

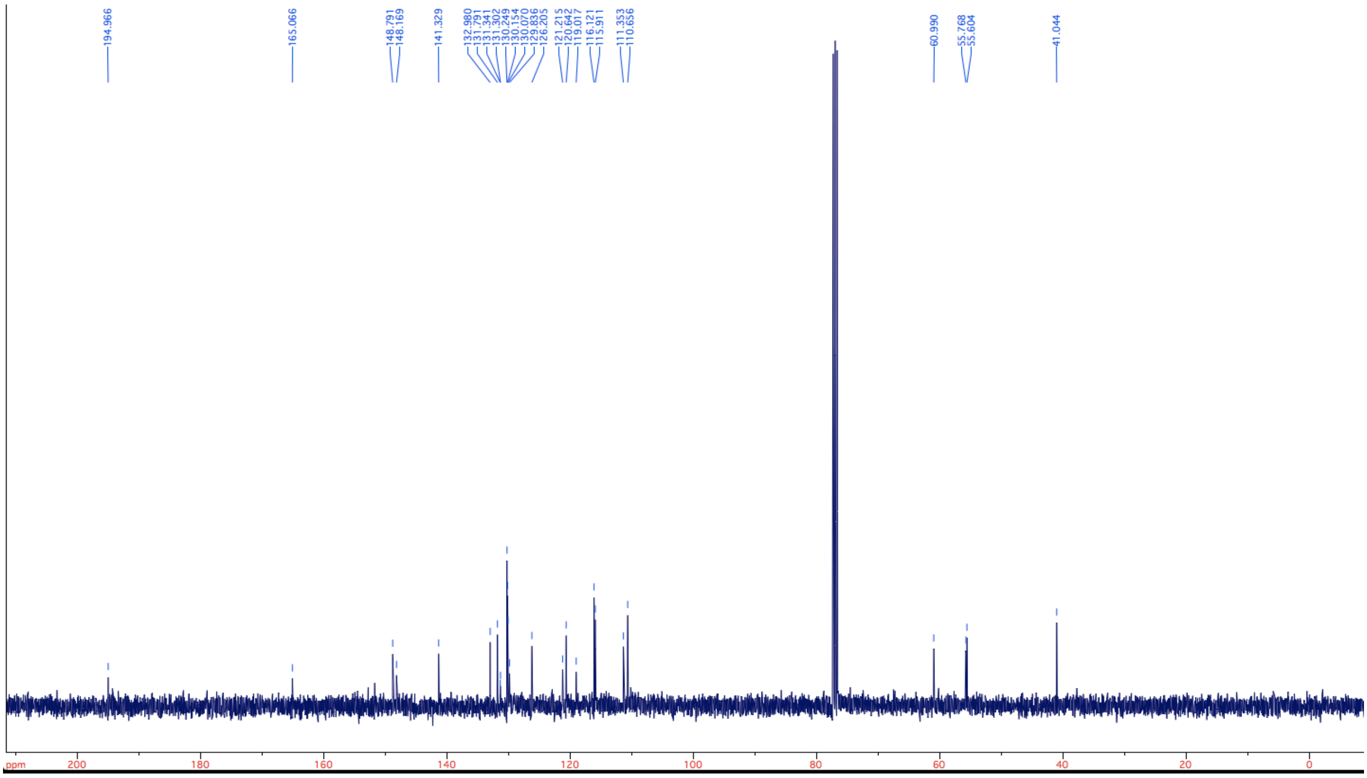

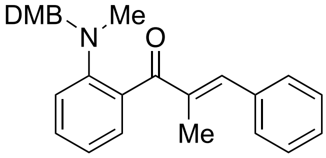

9v

^{1}H NMR (400 MHz, $CDCl_3$)

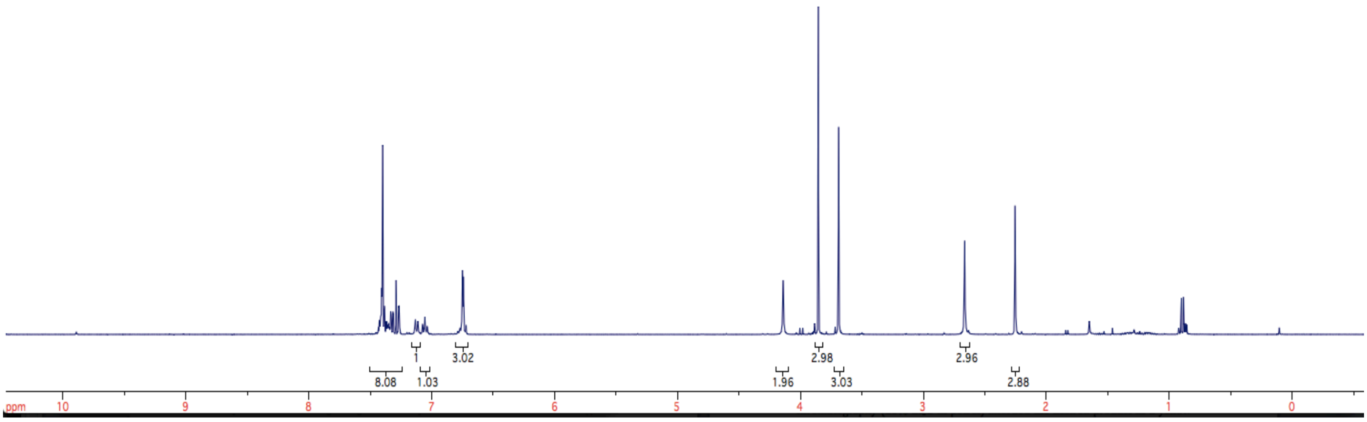

^{13}C NMR (100 MHz, $CDCl_3$)

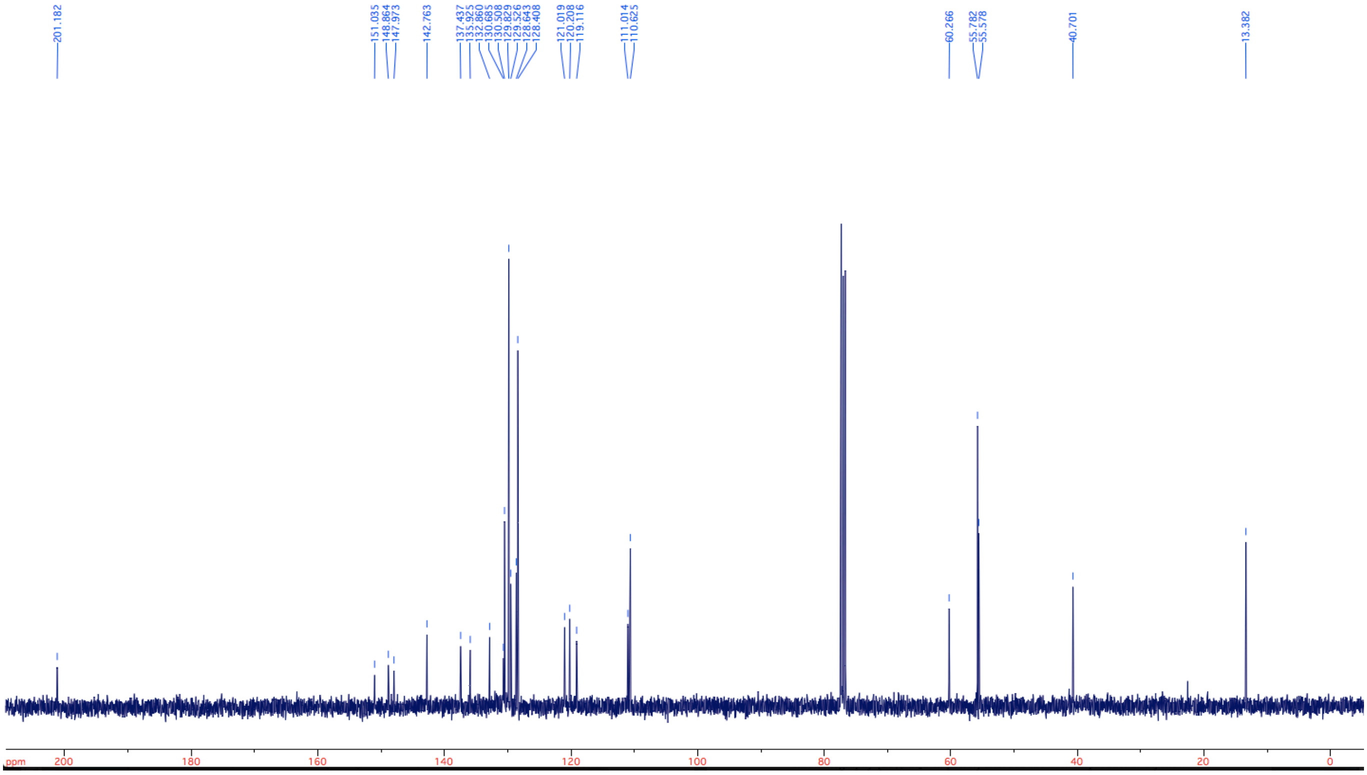

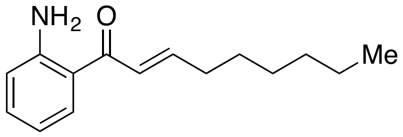

9w

^{1}H NMR (400 MHz, $CDCl_3$)

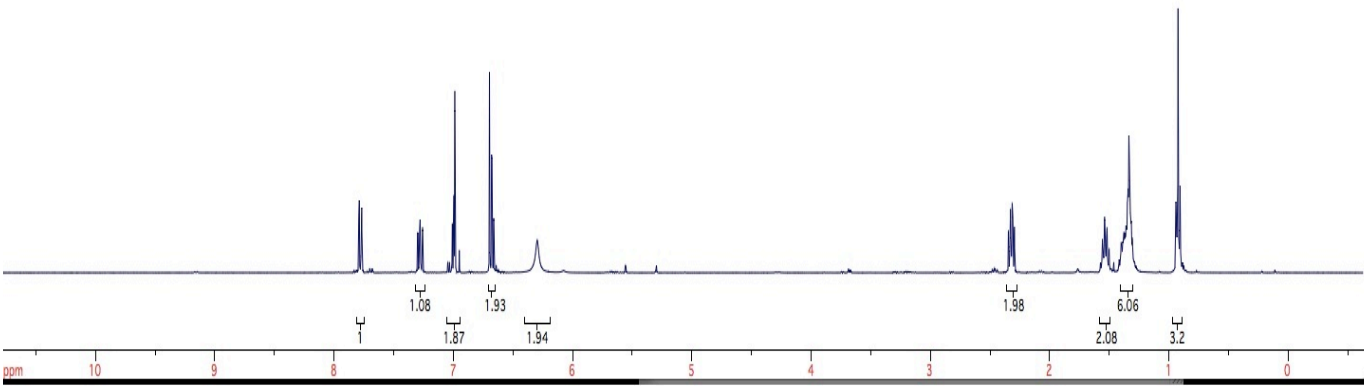

^{13}C NMR (100 MHz, $CDCl_3$)

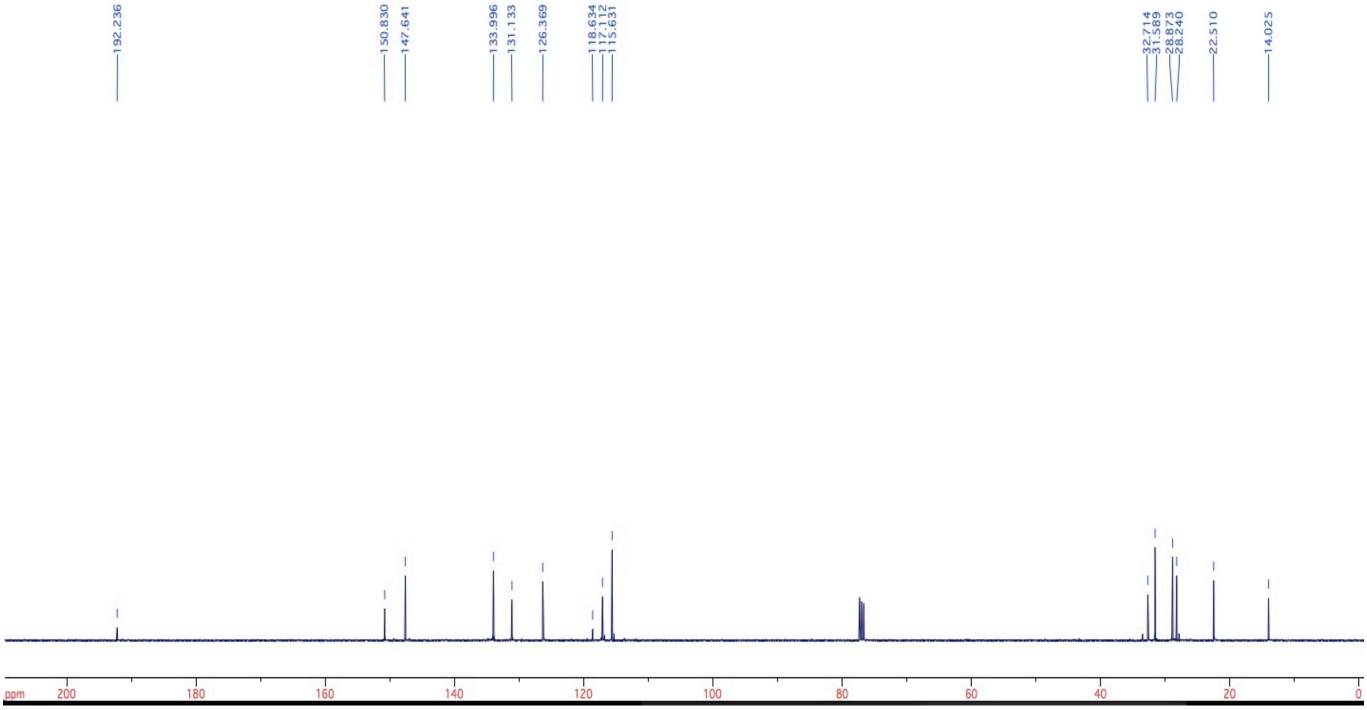

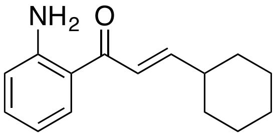

9x

^{1}H NMR (400 MHz, $CDCl_3$)

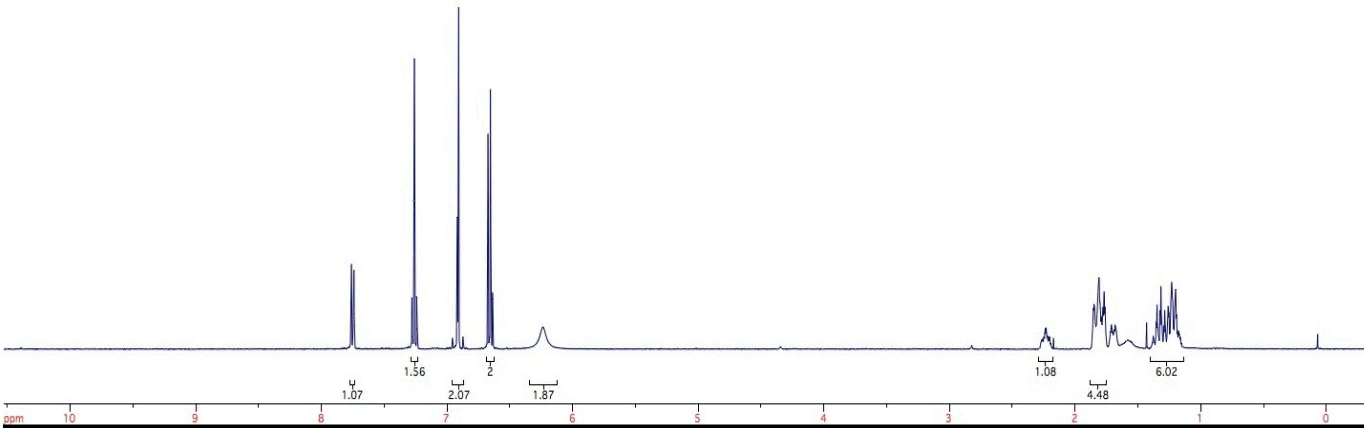

^{13}C NMR (100 MHz, $CDCl_3$)

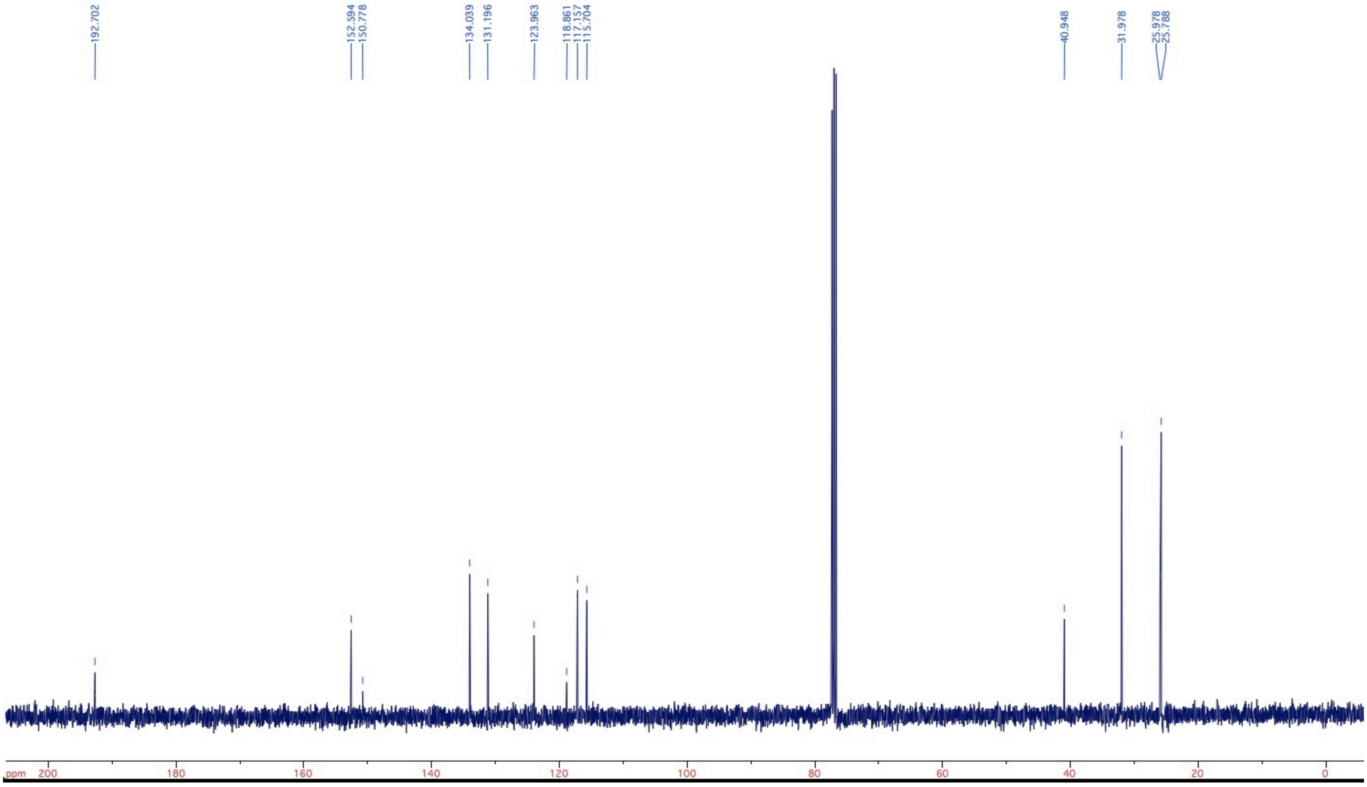

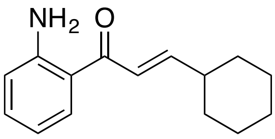

9x

DEPT135 (100 MHz, $CDCl_3$)

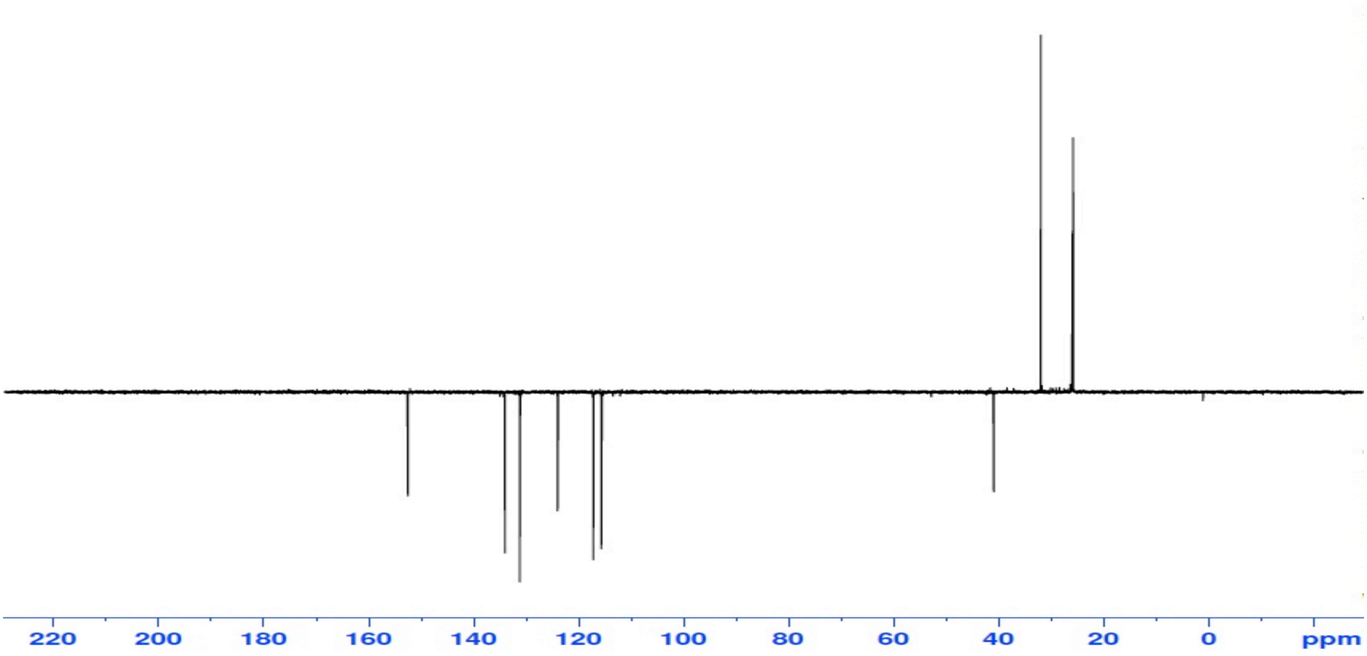

DEPTQ (100 MHz, $CDCl_3$)

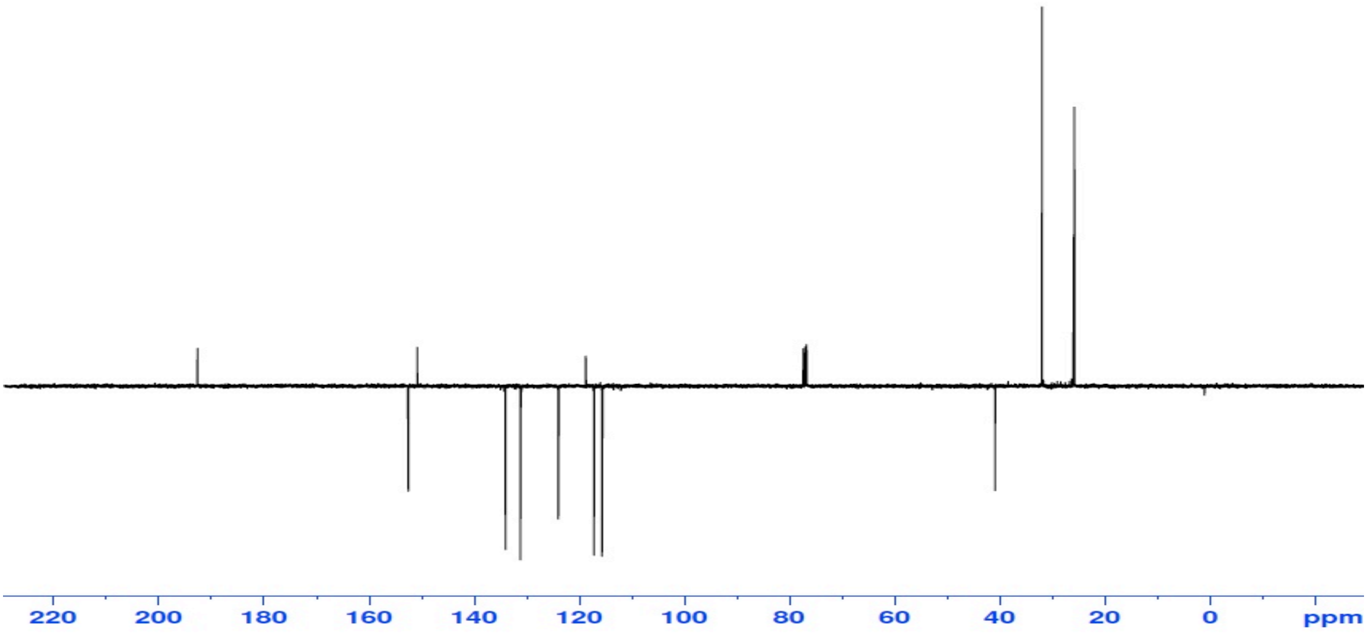

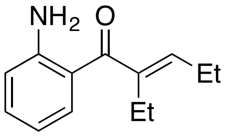

9y

^{1}H NMR (400 MHz, $CDCl_3$)

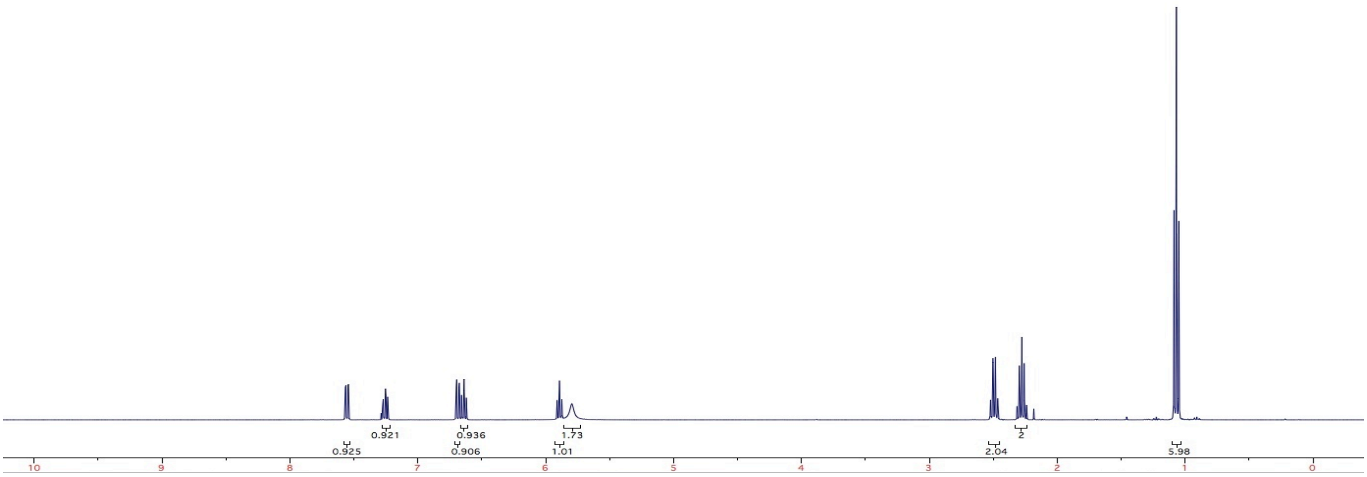

^{13}C NMR (100 MHz, $CDCl_3$)

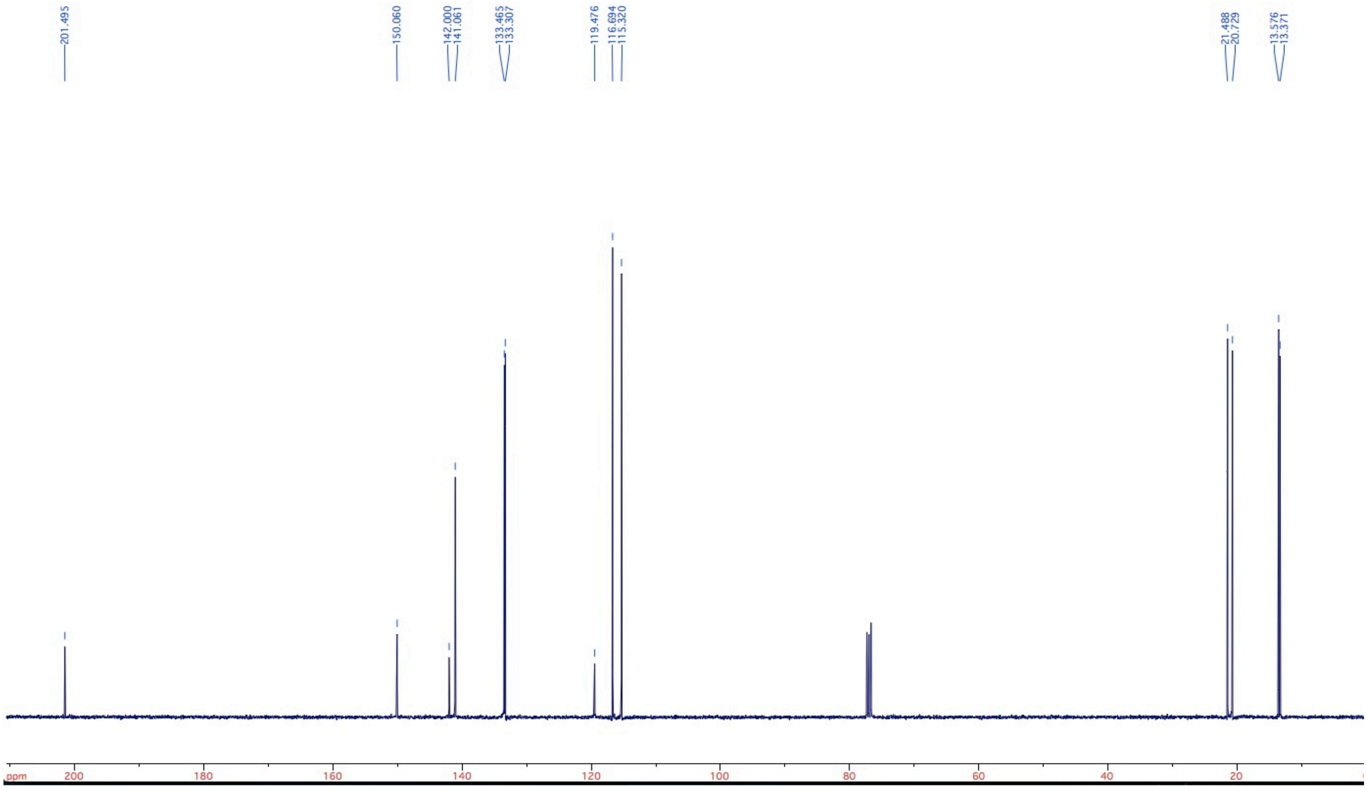

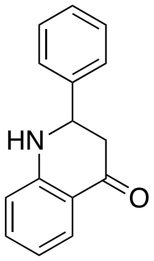

10a

^{1}H NMR (400 MHz, $CDCl_3$)

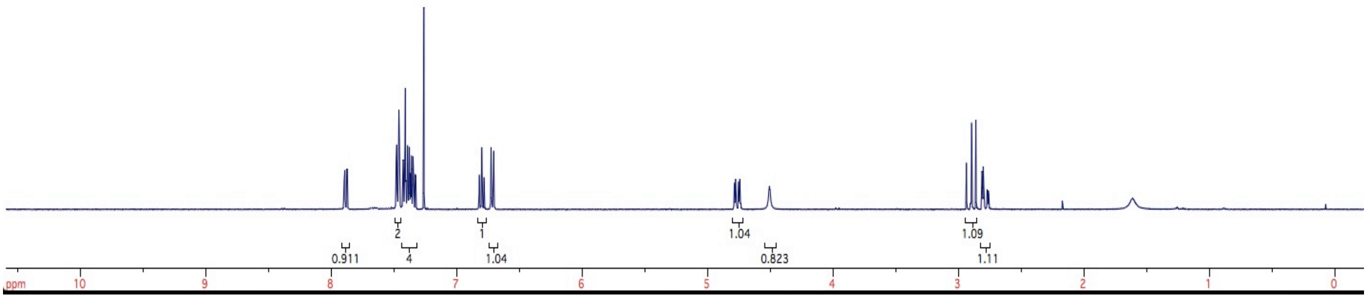

^{13}C NMR (100 MHz, $CDCl_3$)

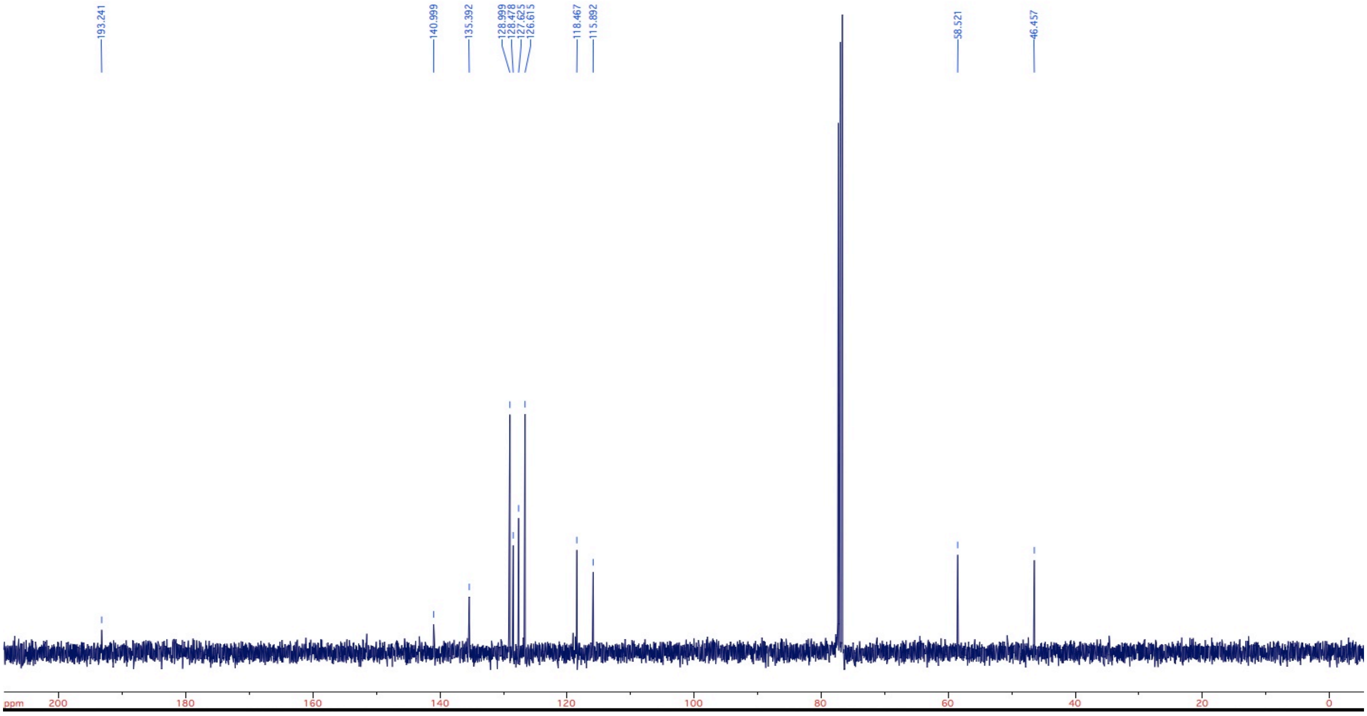

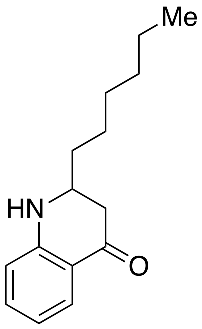

10b

^{1}H NMR (400 MHz, $CDCl_3$)

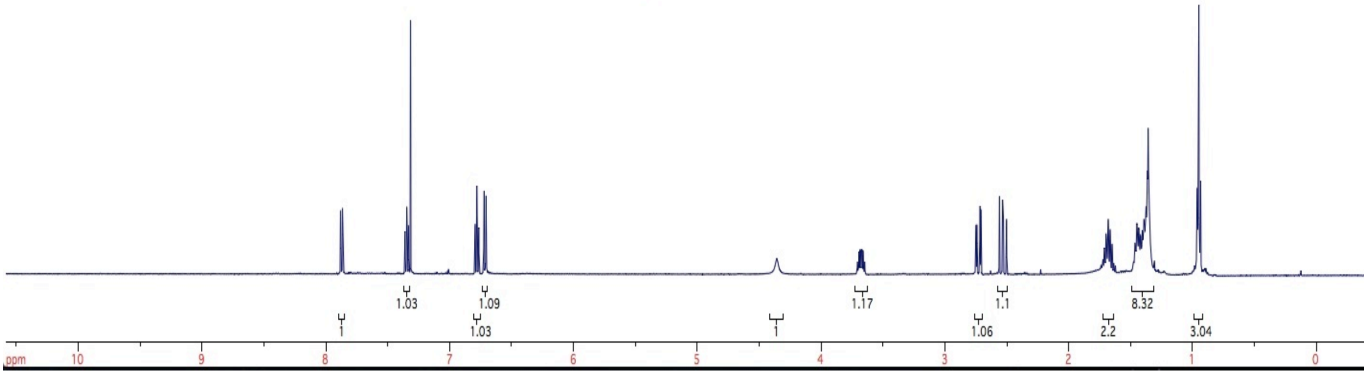

^{13}C NMR (100 MHz, $CDCl_3$)

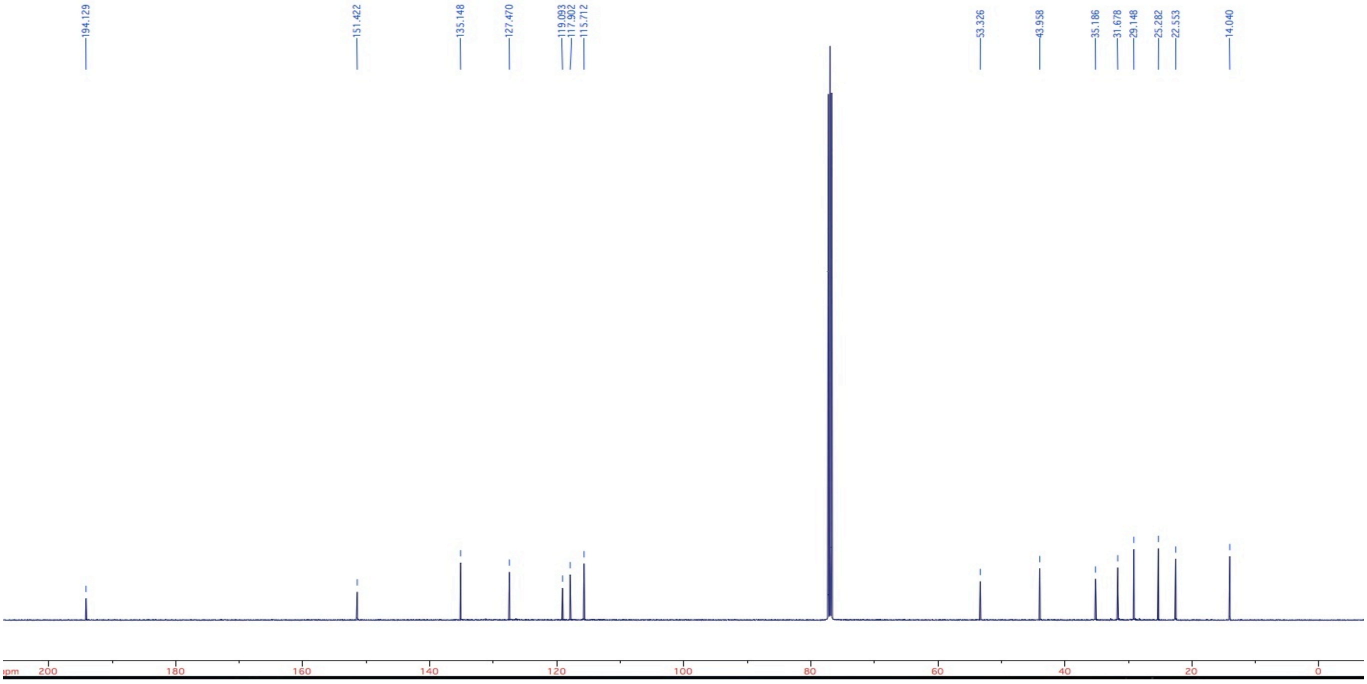

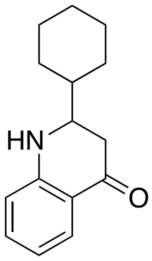

10c

^{1}H NMR (400 MHz, $CDCl_3$)

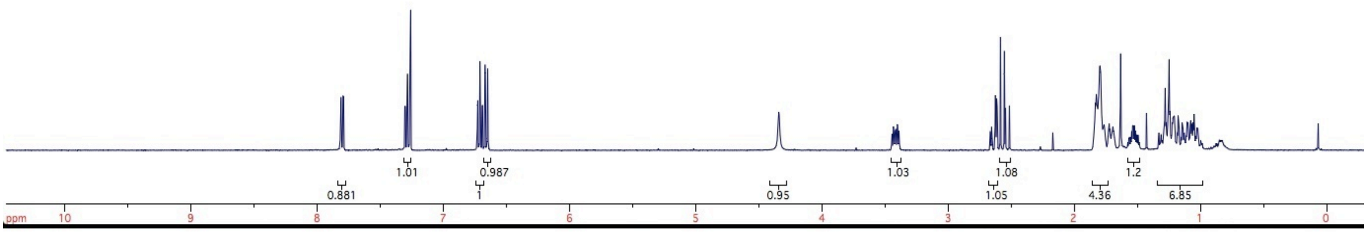

^{13}C NMR (100 MHz, $CDCl_3$)

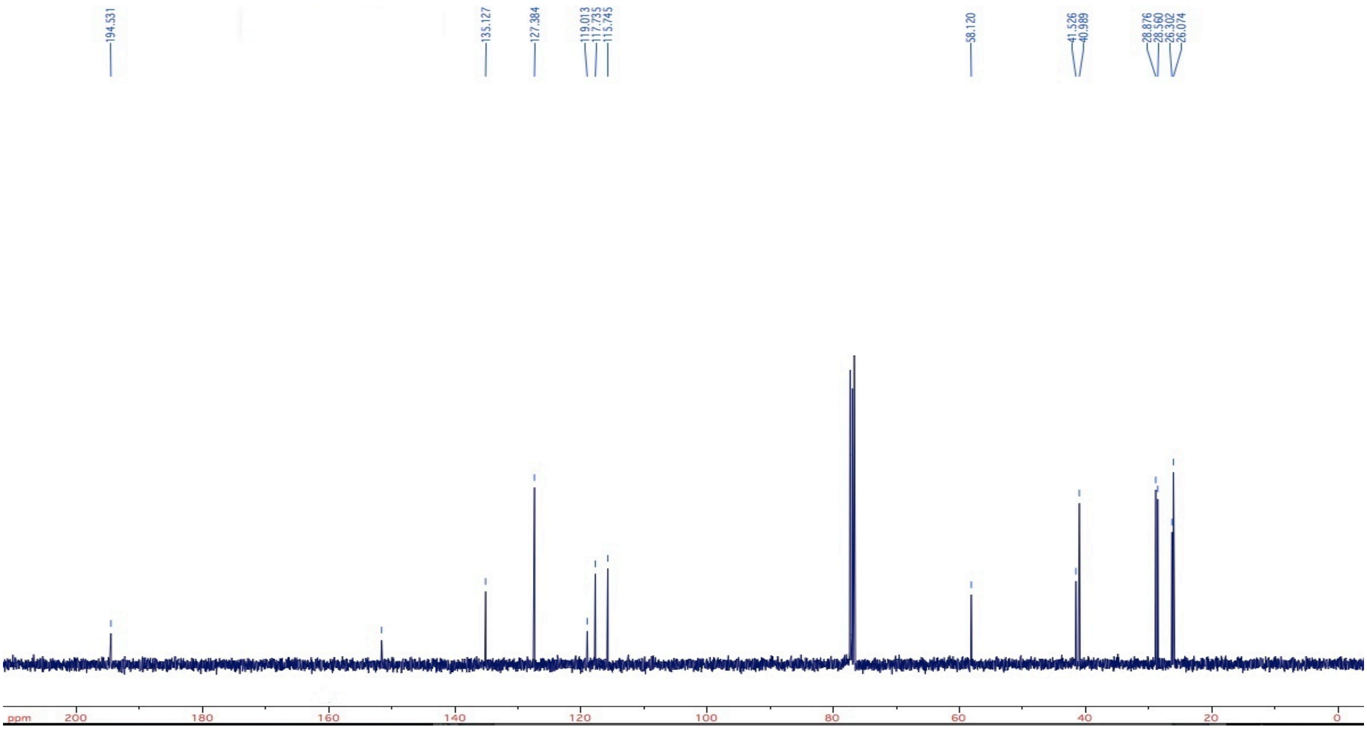

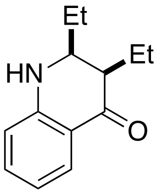

10d'

^{1}H NMR (400 MHz, $CDCl_3$)

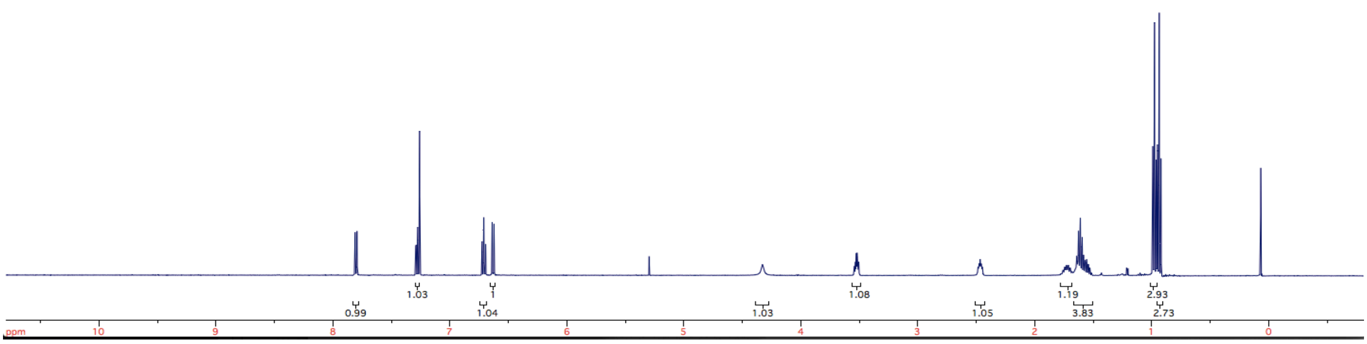

^{13}C NMR (100 MHz, $CDCl_3$)

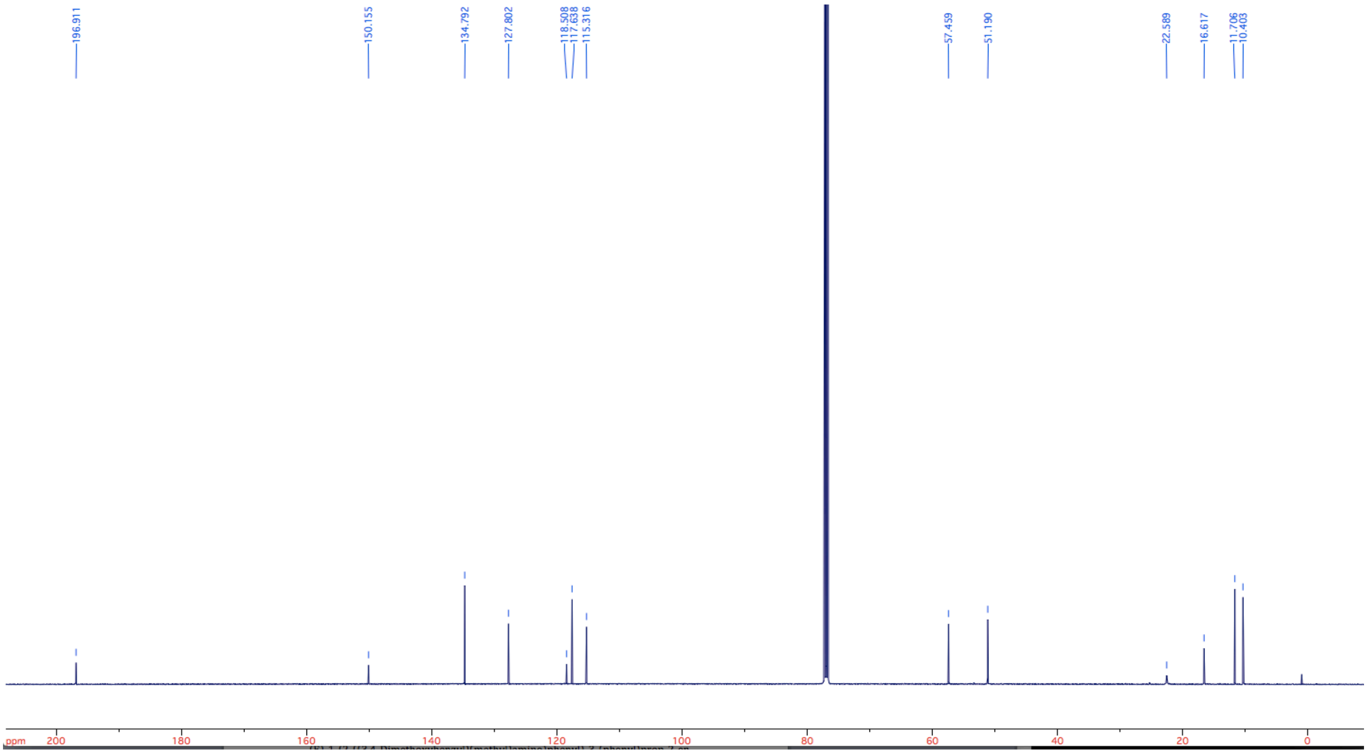

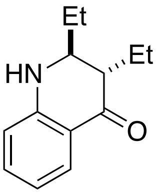

10d

^{1}H NMR (400 MHz, $CDCl_3$)

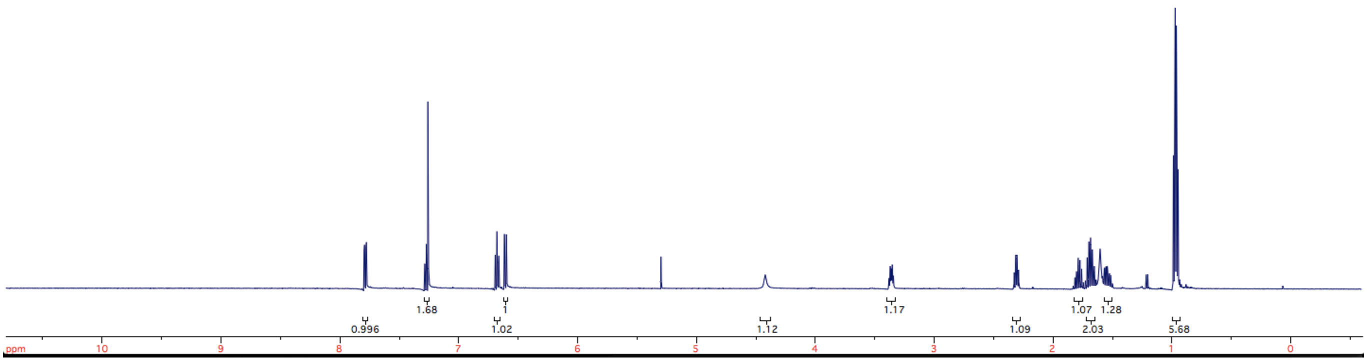

^{13}C NMR (100 MHz, $CDCl_3$)

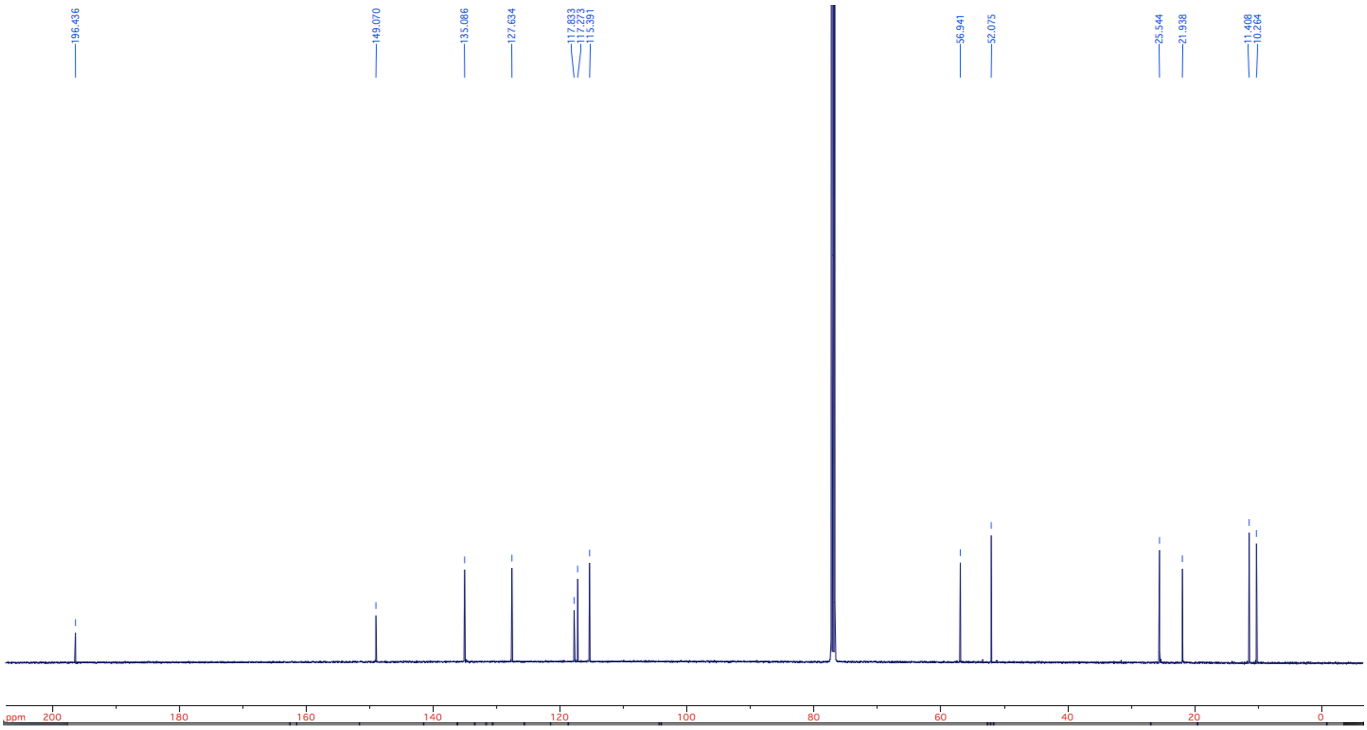

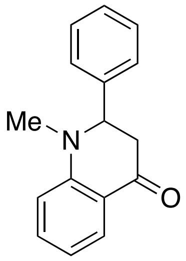

10e

^{1}H NMR (400 MHz, $CDCl_3$)

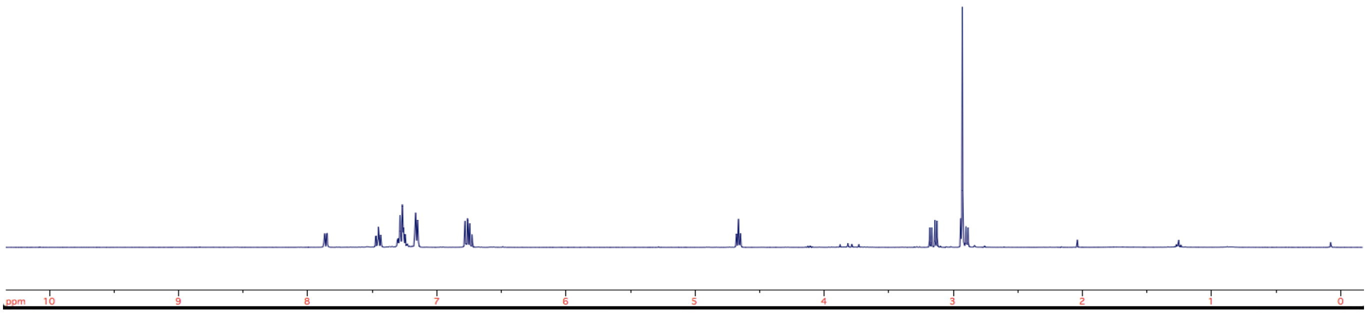

^{13}C NMR (100 MHz, $CDCl_3$)

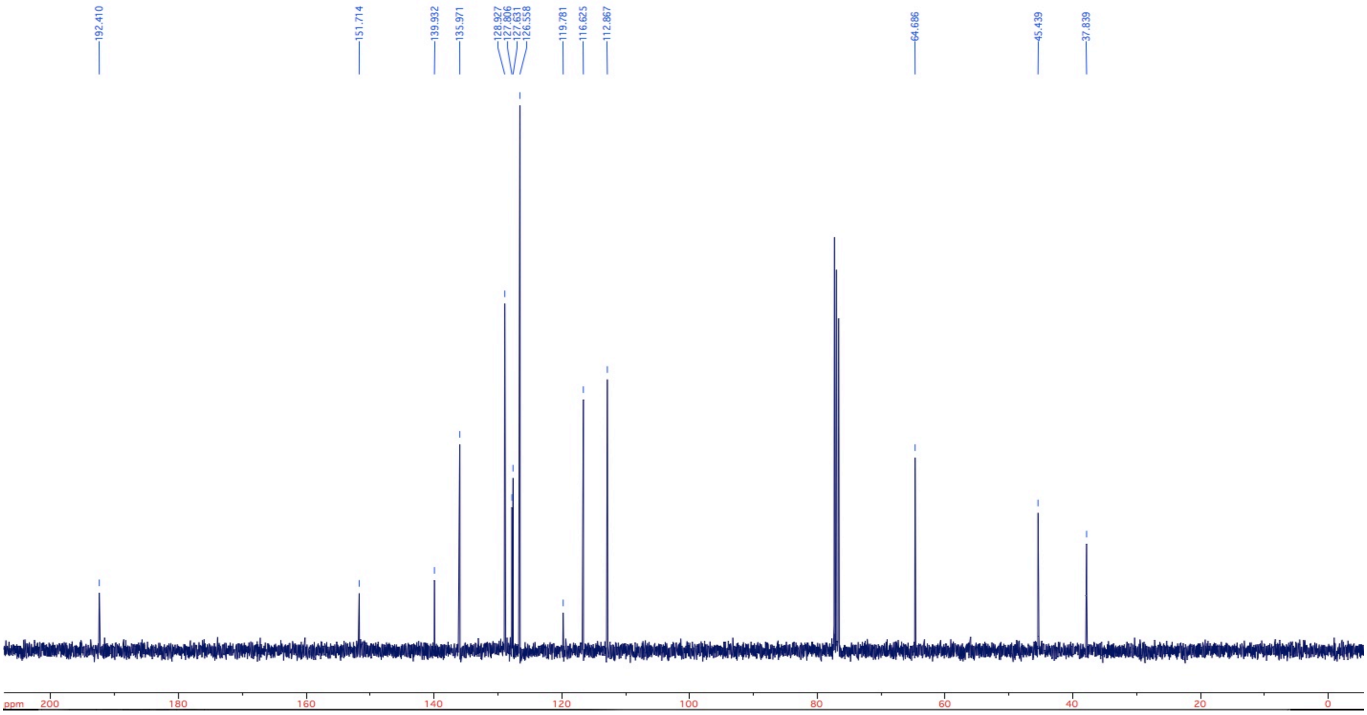

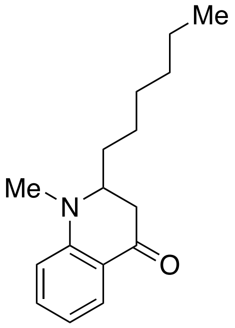

10f

^{1}H NMR (400 MHz, $CDCl_3$)

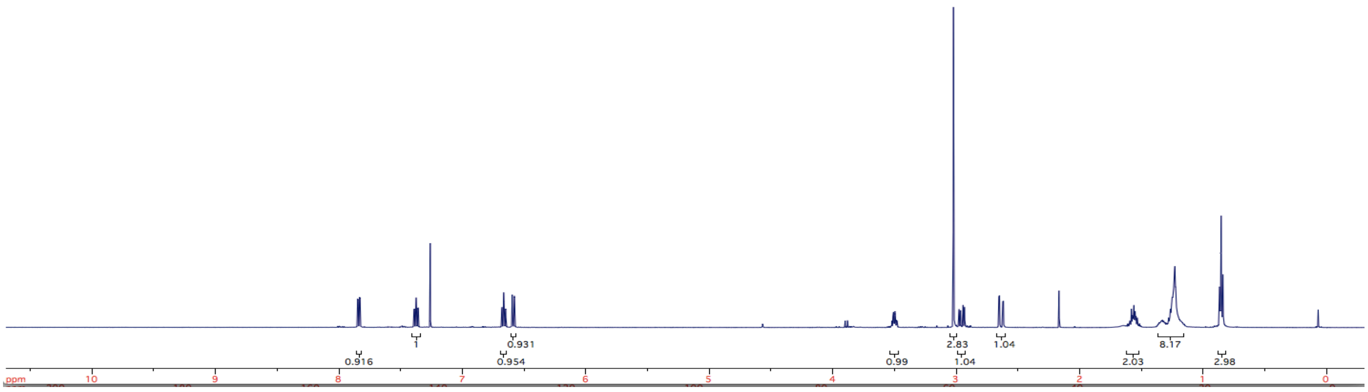

^{13}C NMR (100 MHz, $CDCl_3$)

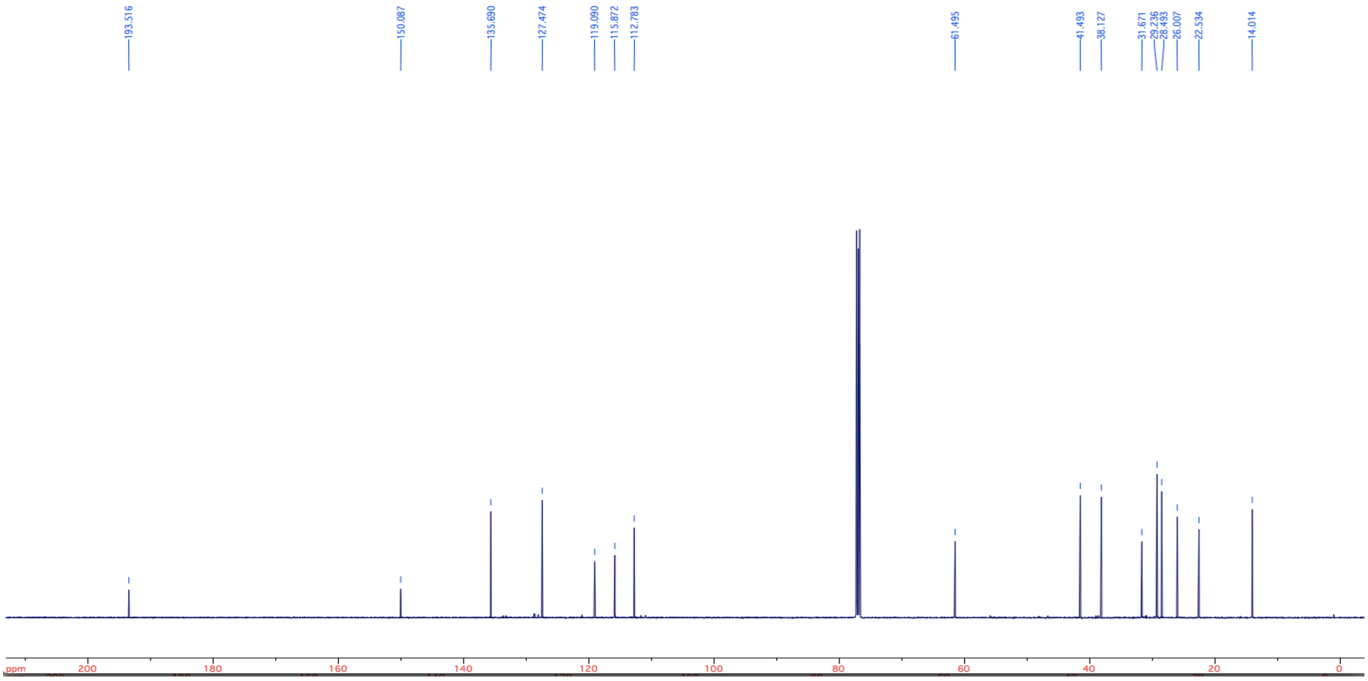

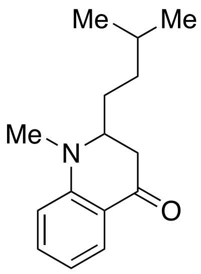

10g

^{1}H NMR (400 MHz, $CDCl_3$)

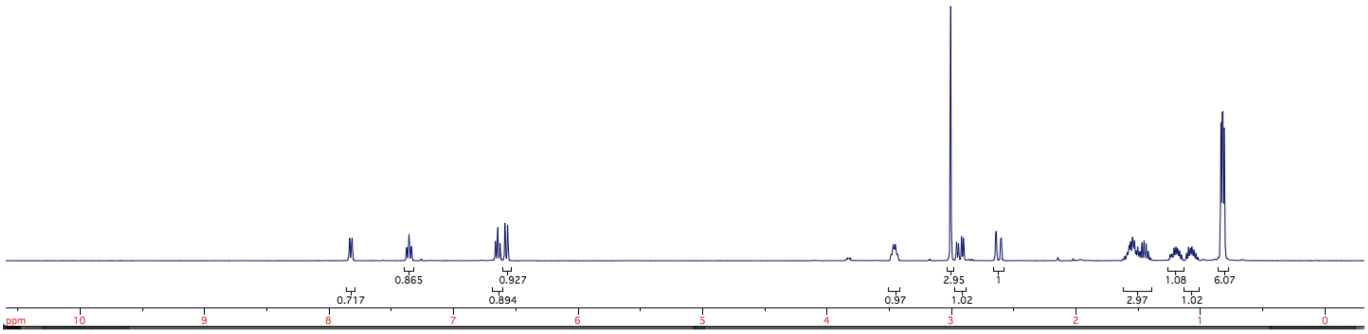

^{13}C NMR (100 MHz, $CDCl_3$)

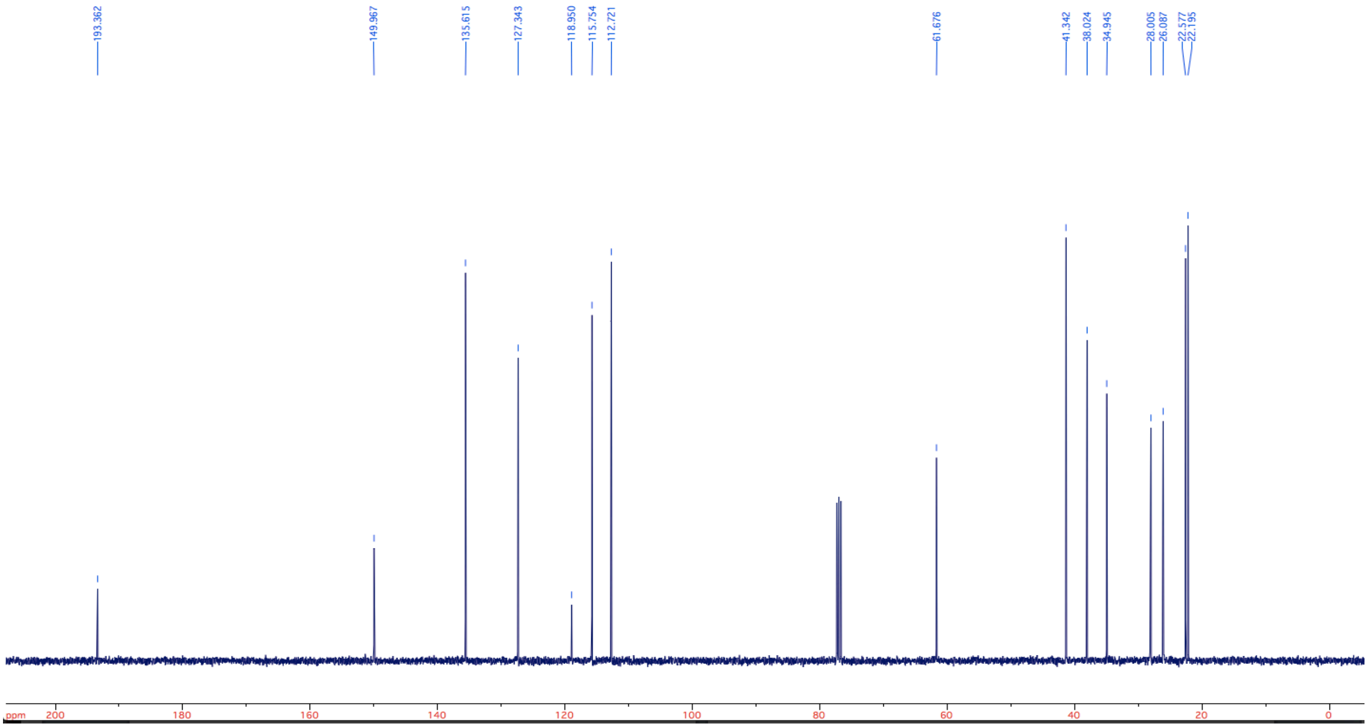

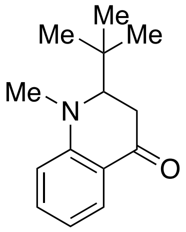

10h

^{1}H NMR (400 MHz, $CDCl_3$)

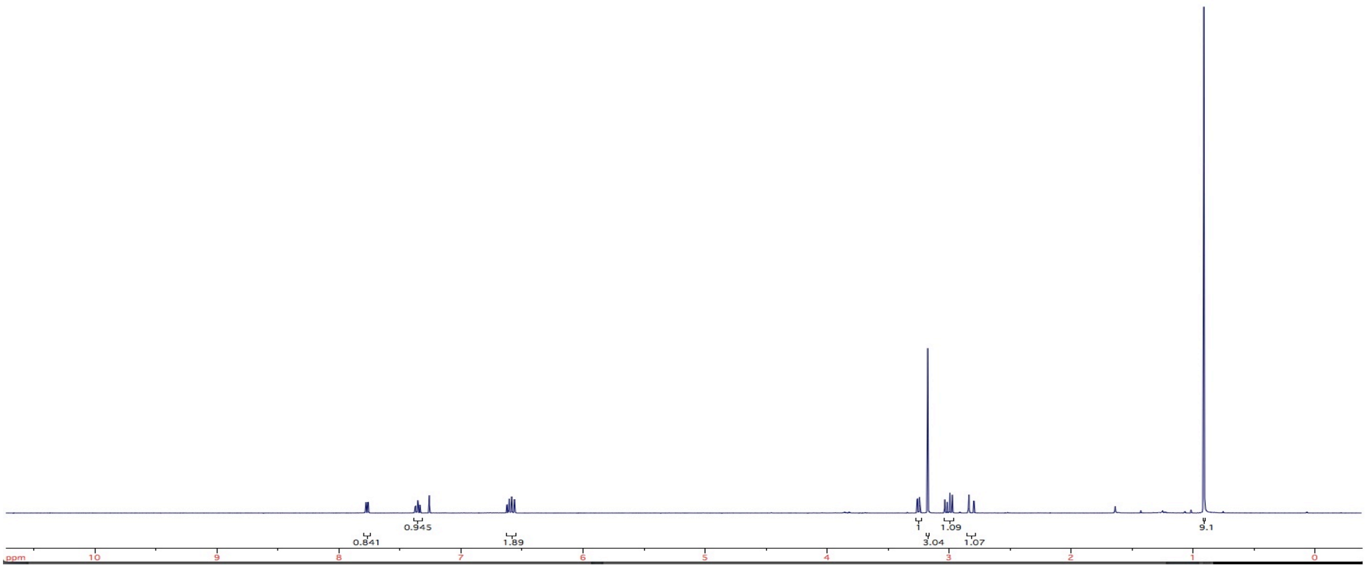

^{13}C NMR (100 MHz, $CDCl_3$)

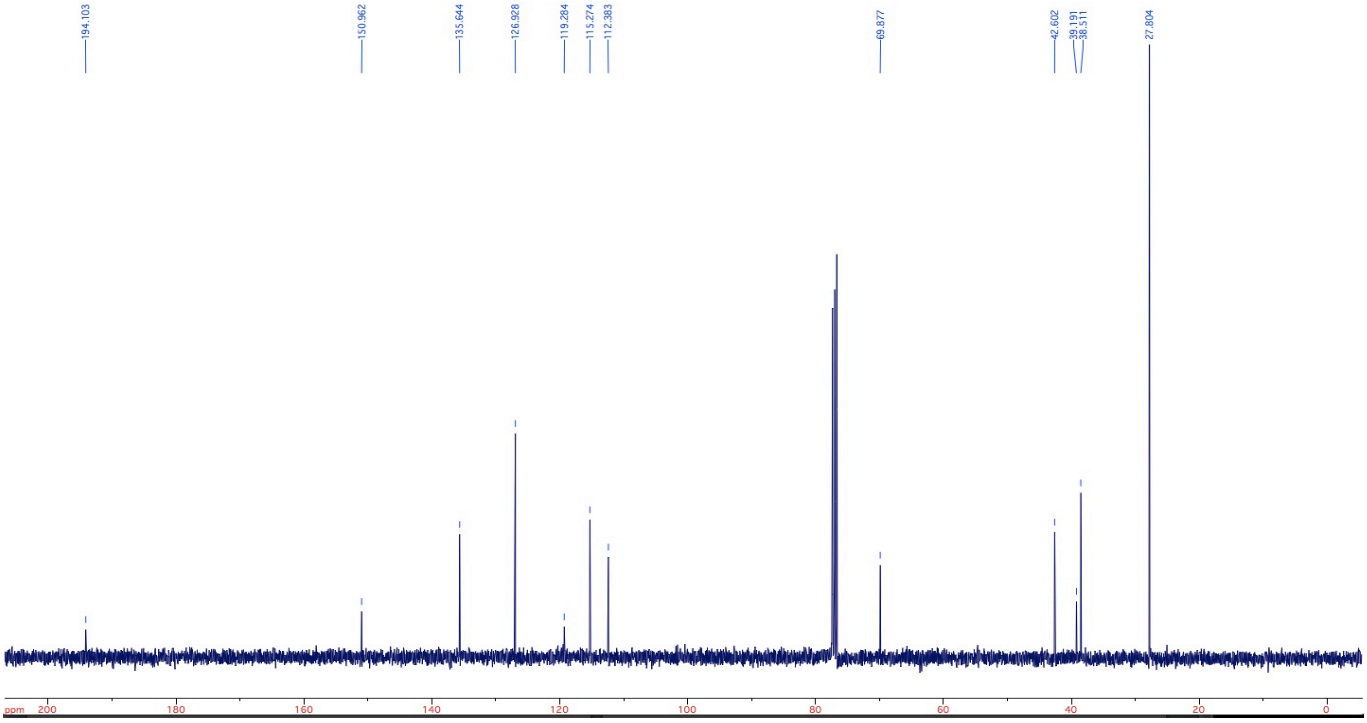

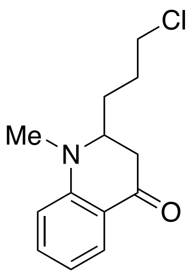

10i

^{1}H NMR (400 MHz, $CDCl_3$)

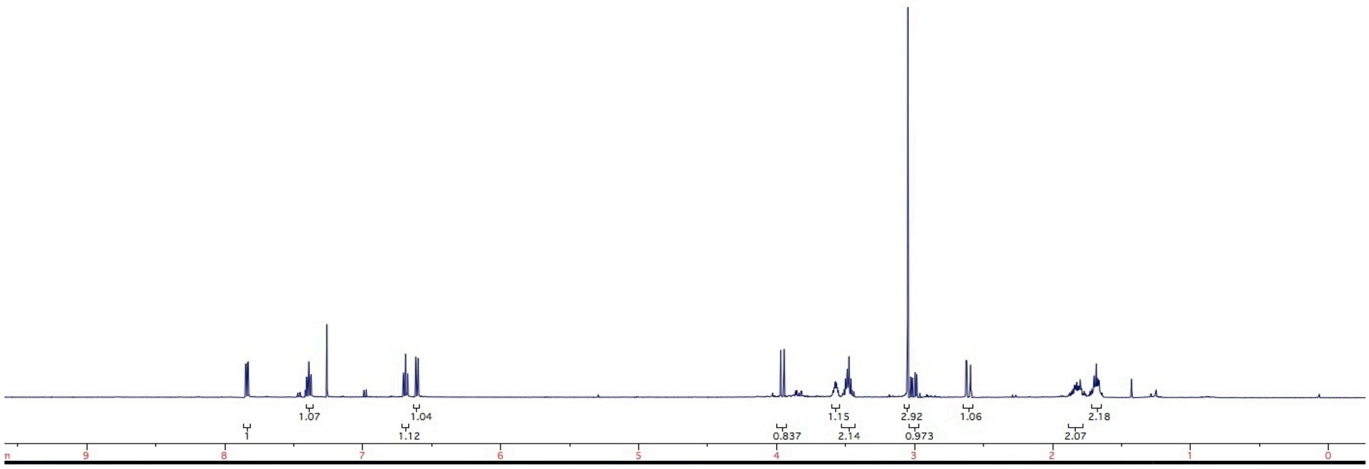

^{13}C NMR (100 MHz, $CDCl_3$)

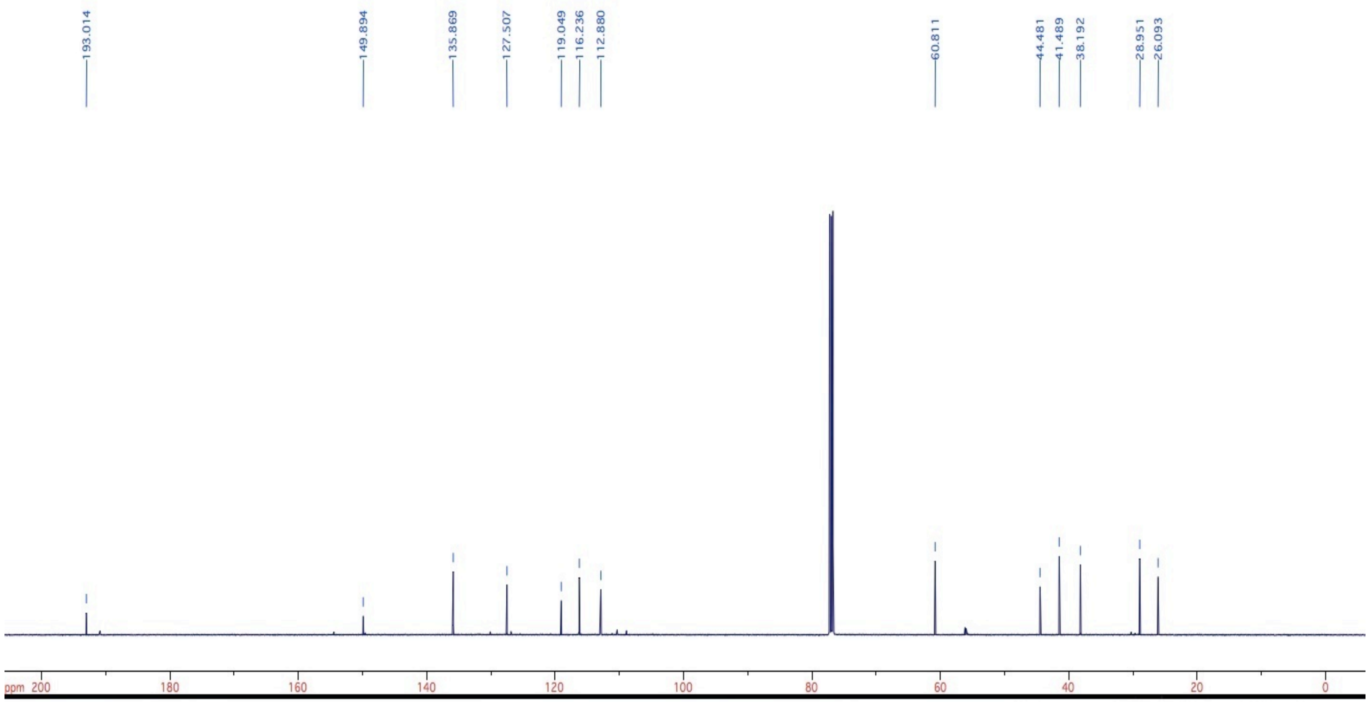

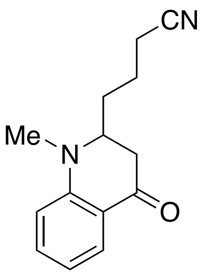

10j

^{1}H NMR (400 MHz, $CDCl_3$)

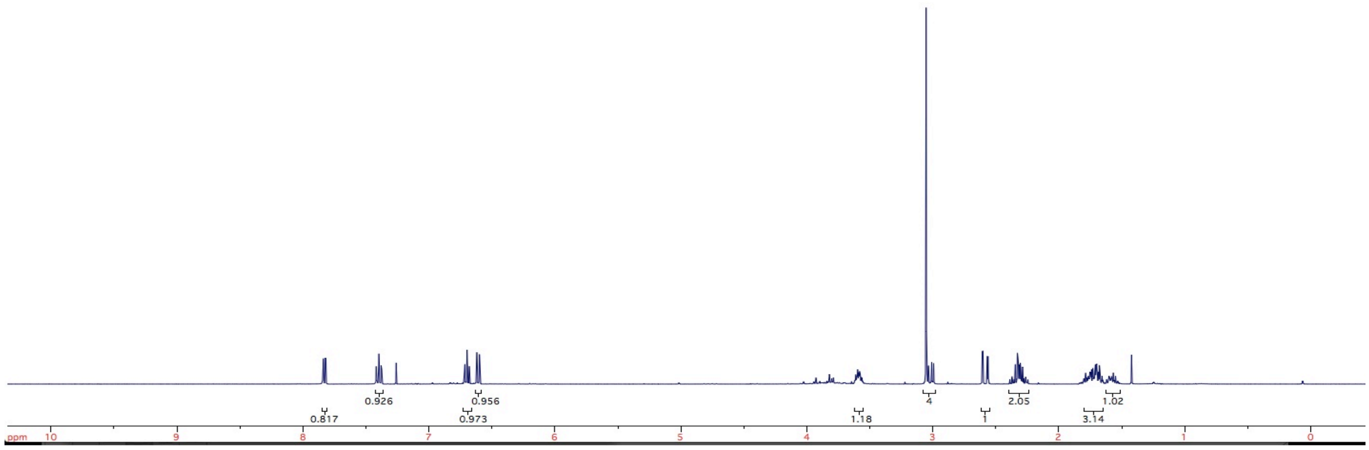

^{13}C NMR (100 MHz, $CDCl_3$)

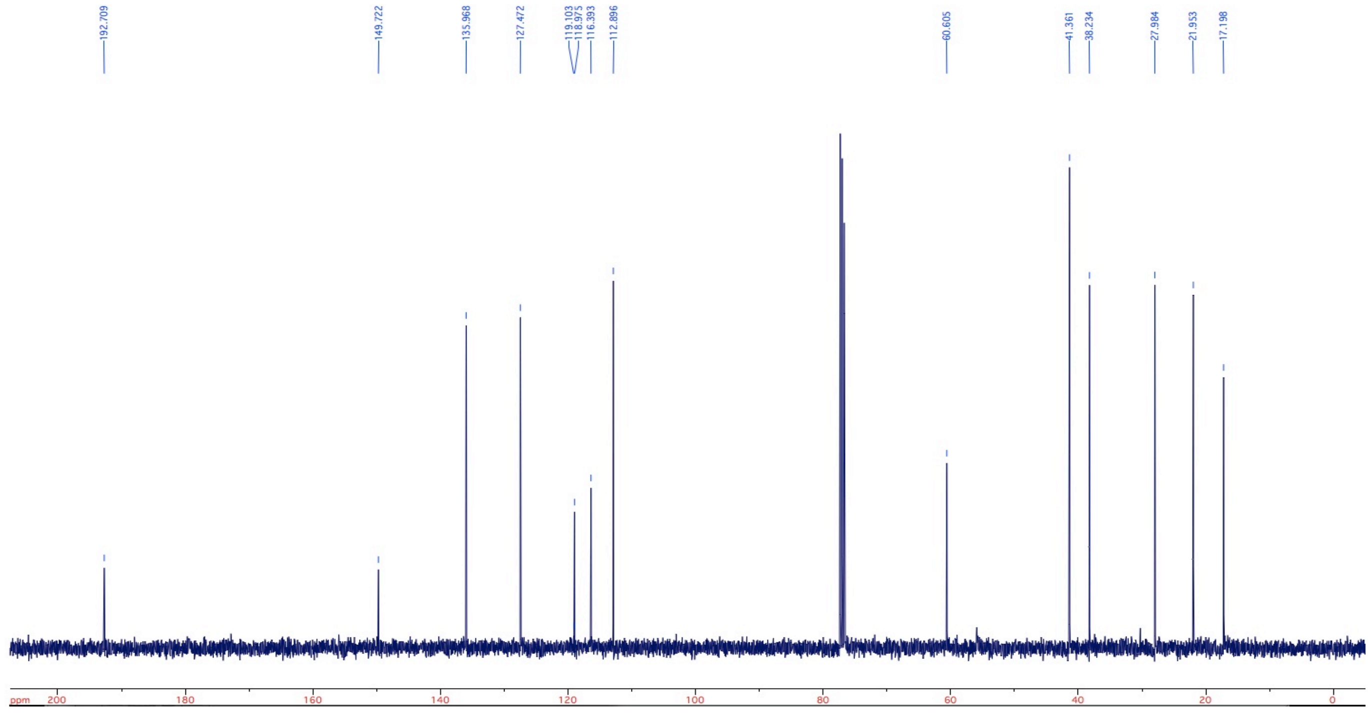

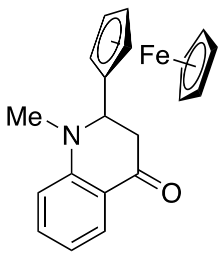

10l

^{1}H NMR (400 MHz, $CDCl_3$)

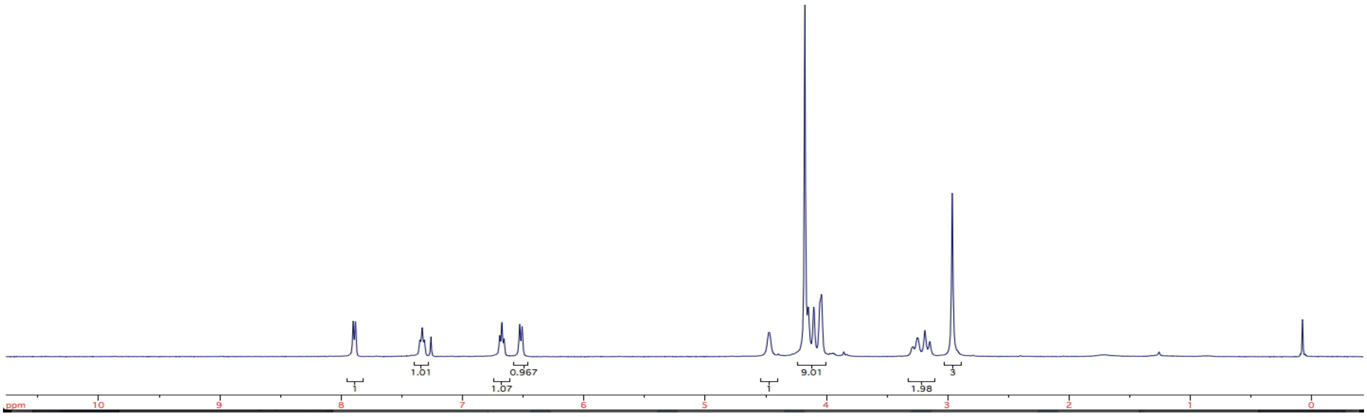

^{13}C NMR (100 MHz, $CDCl_3$)

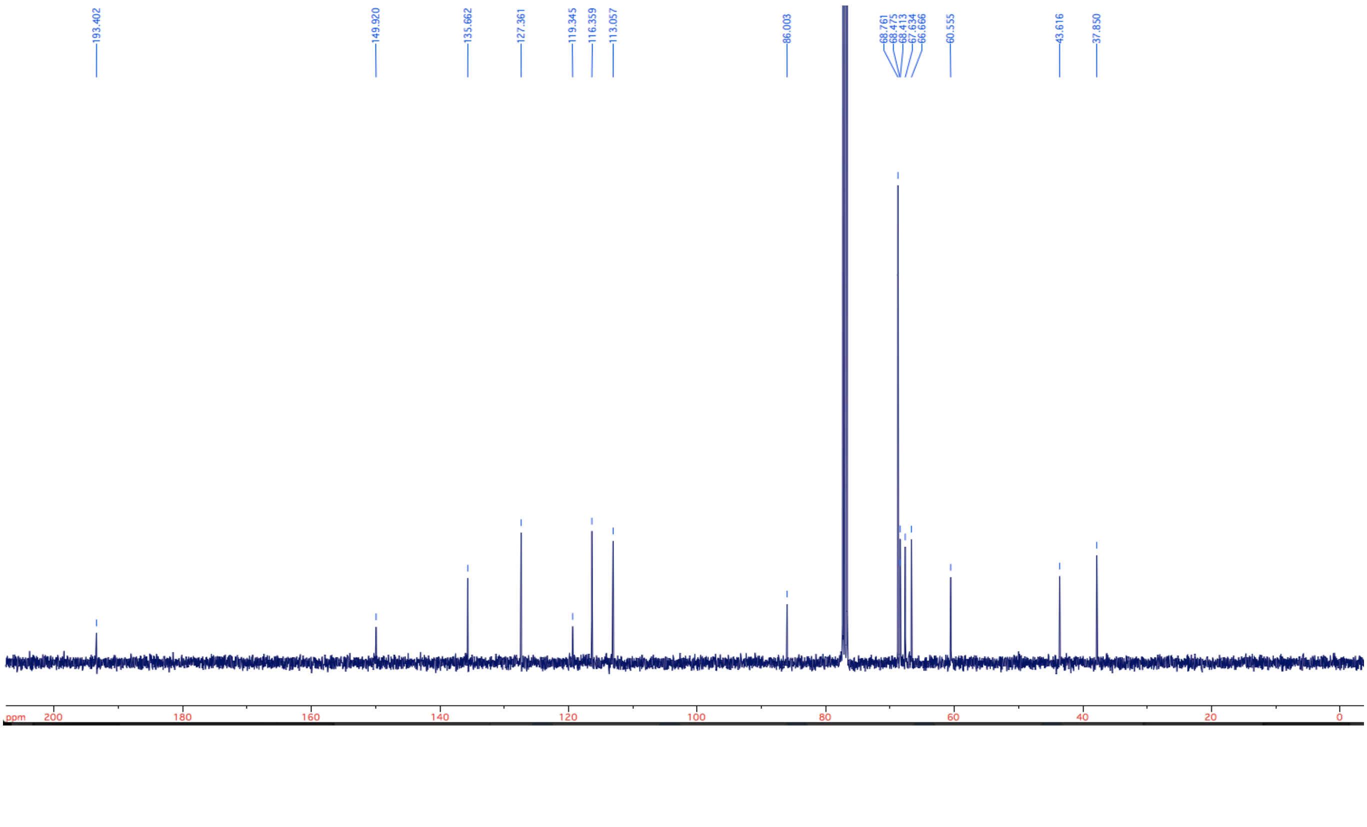

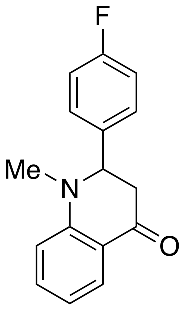

10m

^{1}H NMR (400 MHz, $CDCl_3$)

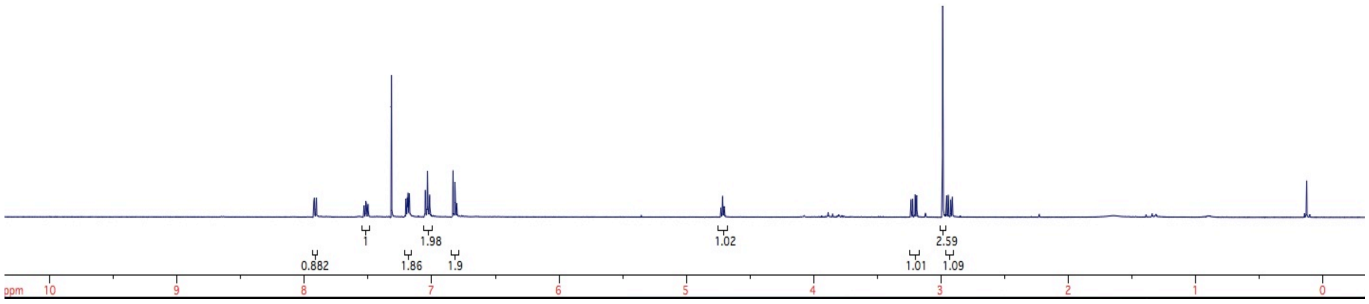

^{13}C NMR (100 MHz, $CDCl_3$)

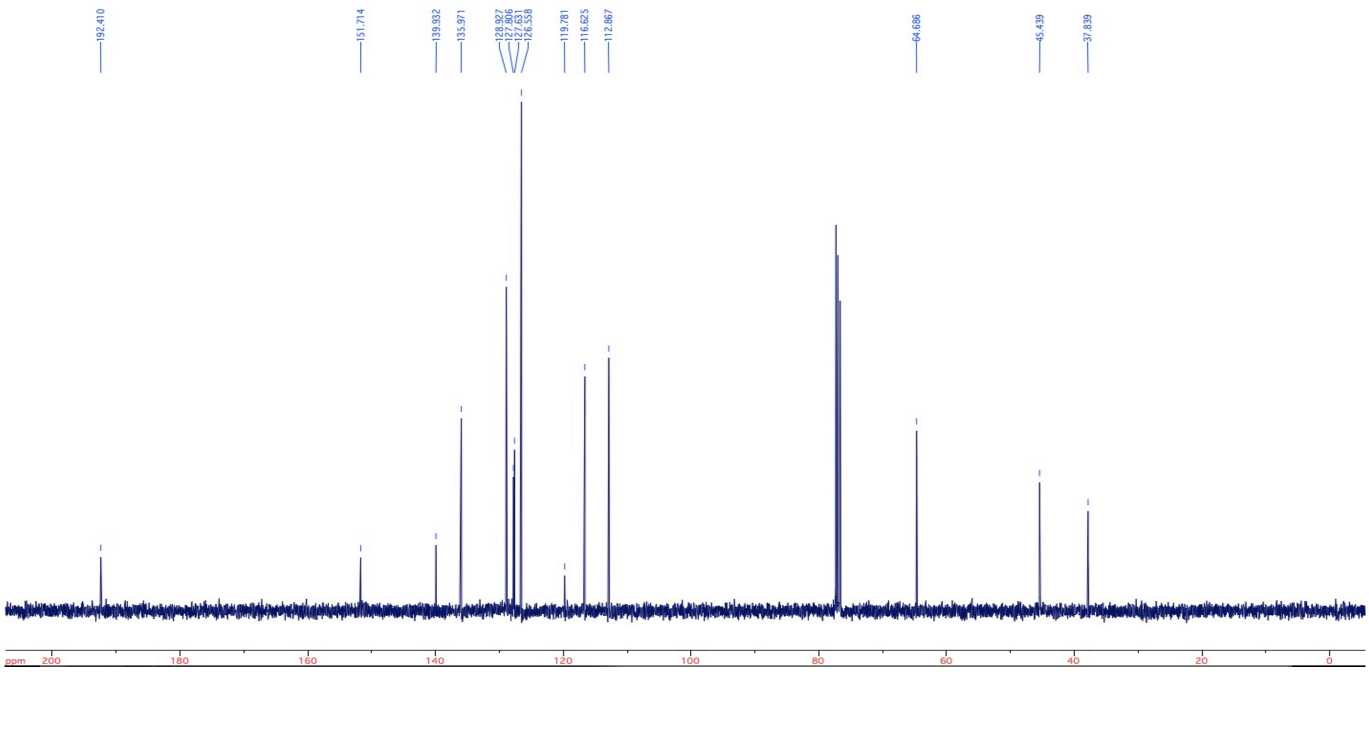

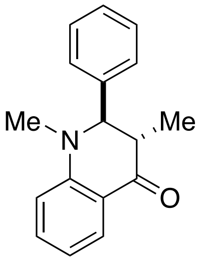

10n'

^1H NMR (400 MHz, $CDCl_3$)

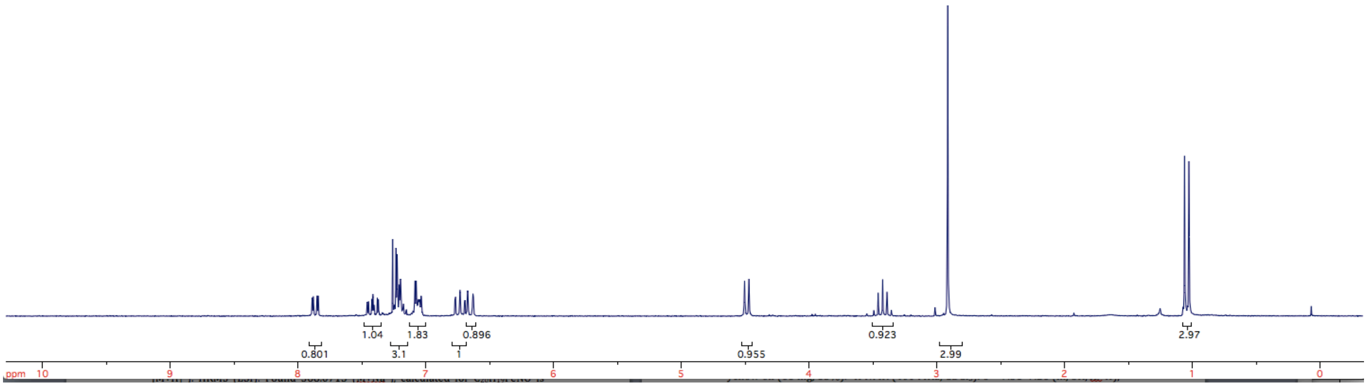

^{13}C NMR (100 MHz, $CDCl_3$)

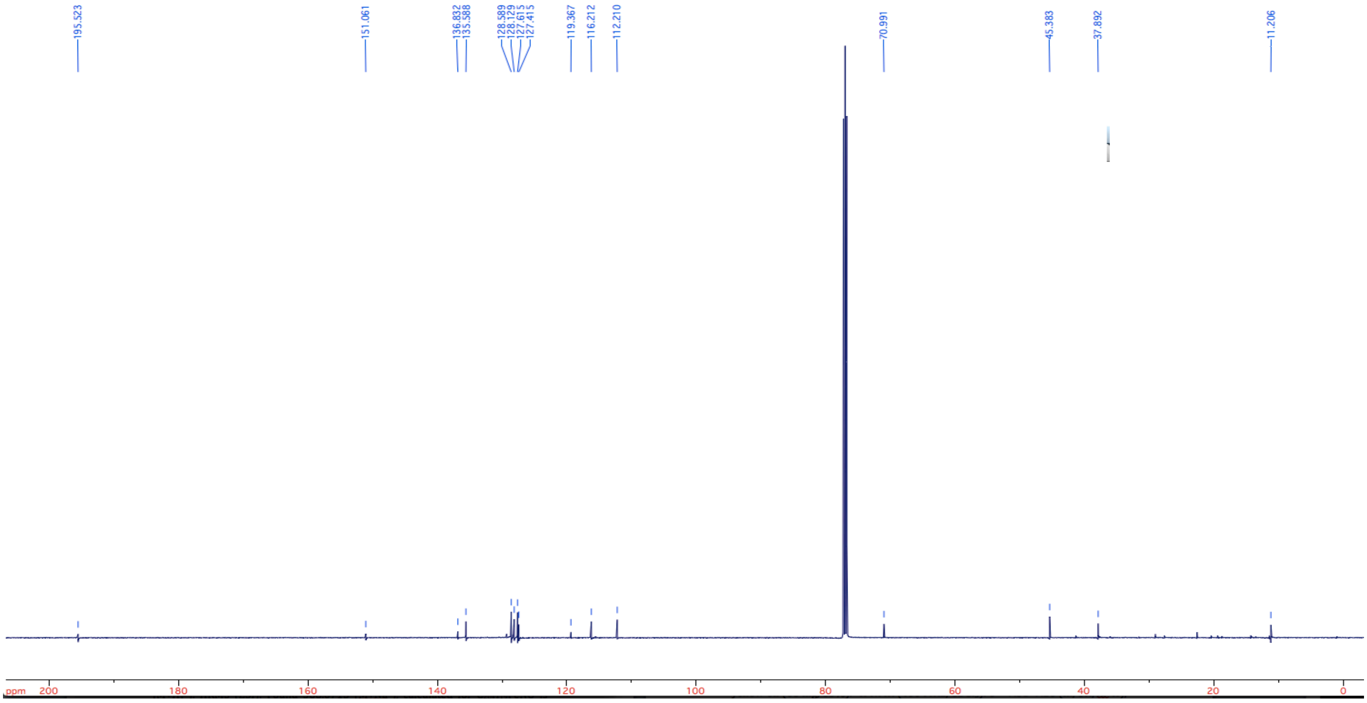

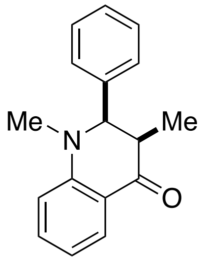

10n

^{1}H NMR (400 MHz, $CDCl_3$)

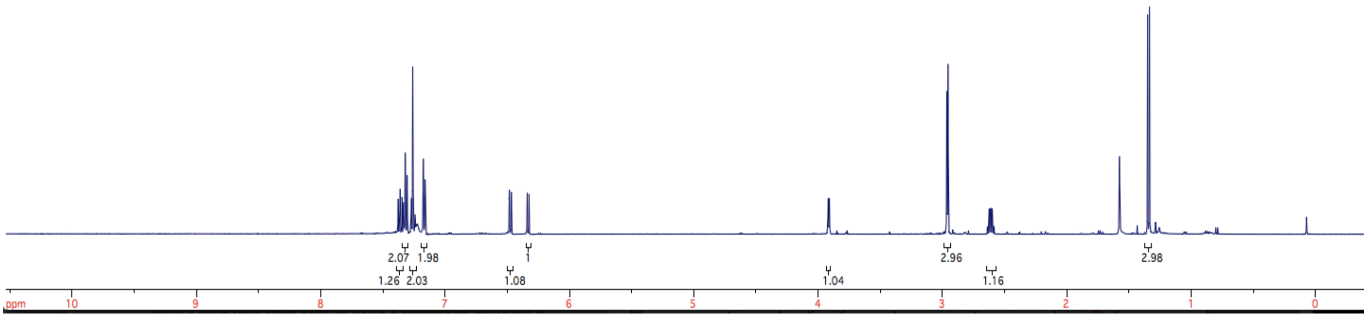

^{13}C NMR (100 MHz, $CDCl_3$)

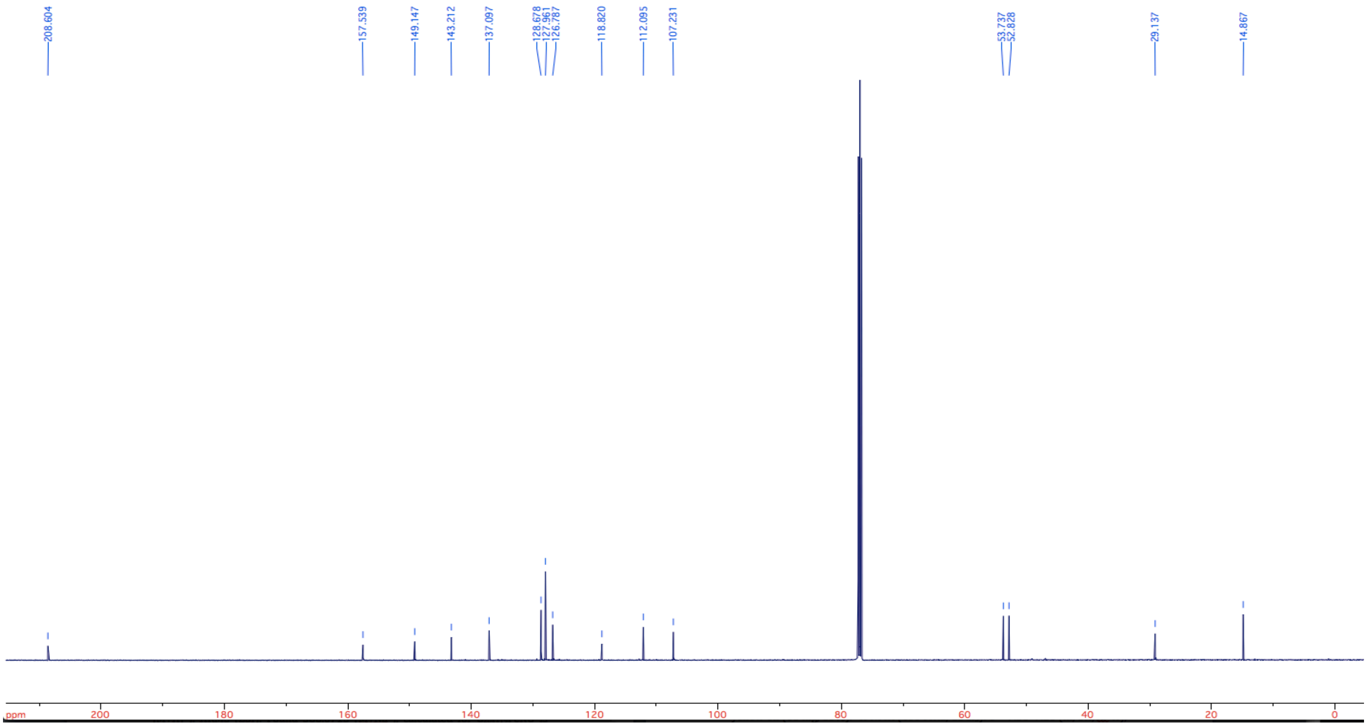

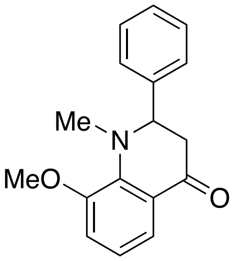

10o

^{1}H NMR (400 MHz, $CDCl_3$)

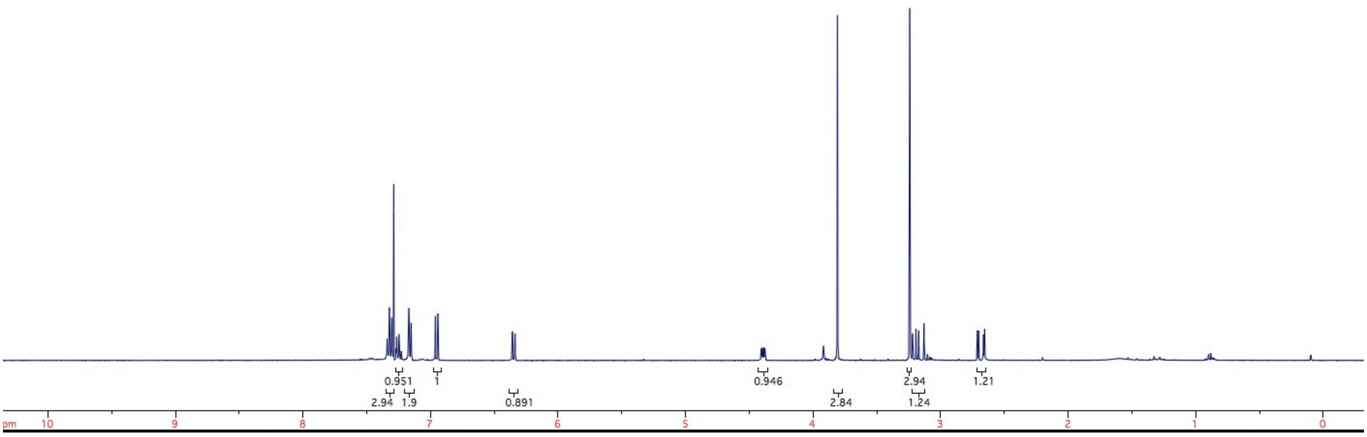

^{13}C NMR (100 MHz, $CDCl_3$)

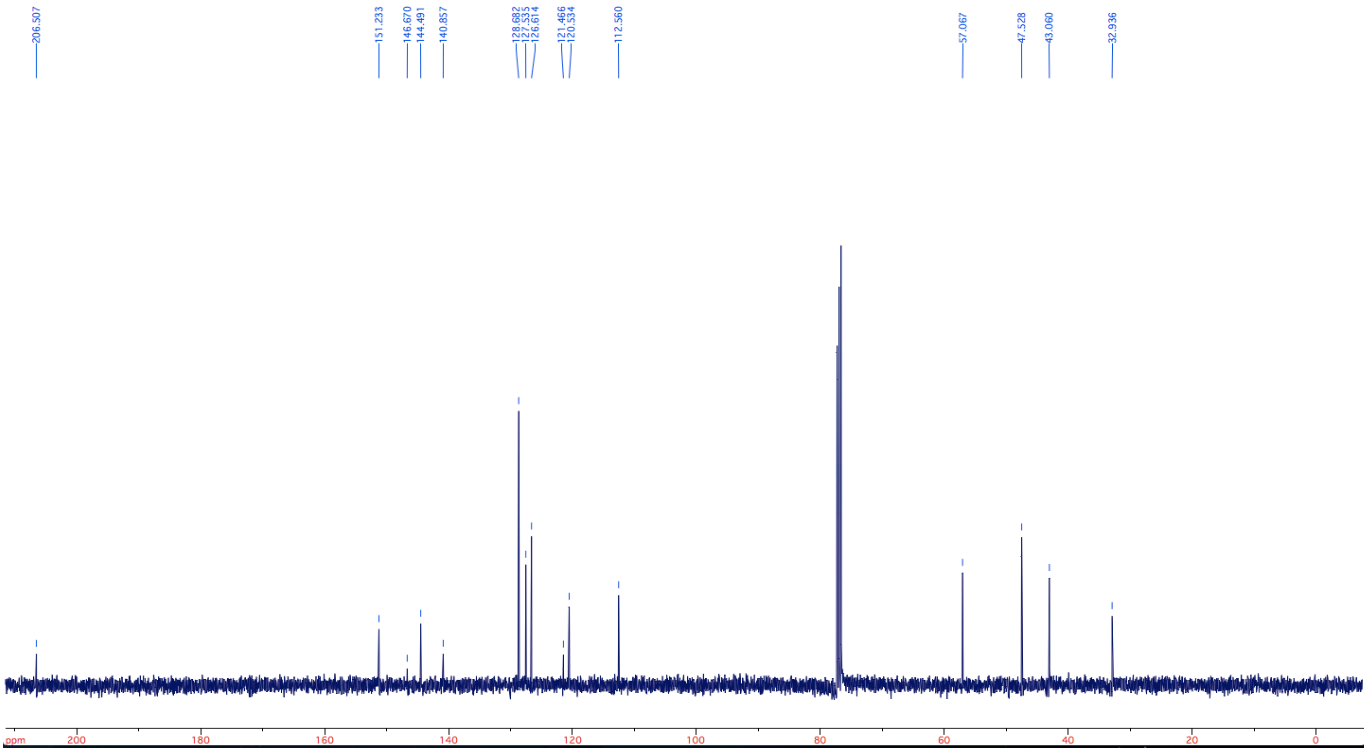

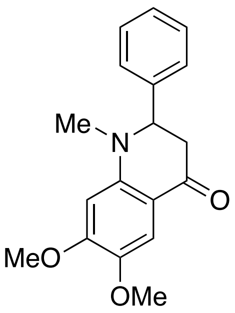

10p

^{1}H NMR (400 MHz, $CDCl_3$)

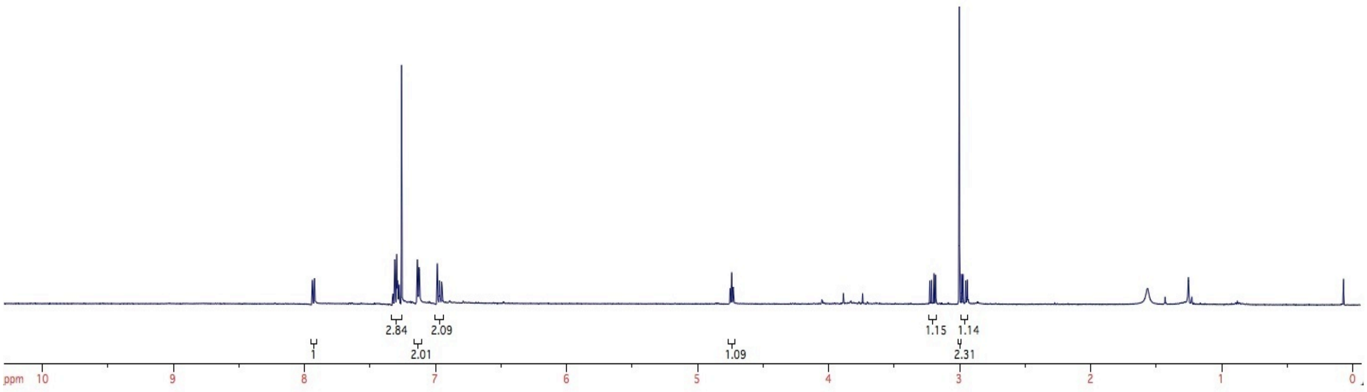

^{13}C NMR (100 MHz, $CDCl_3$)

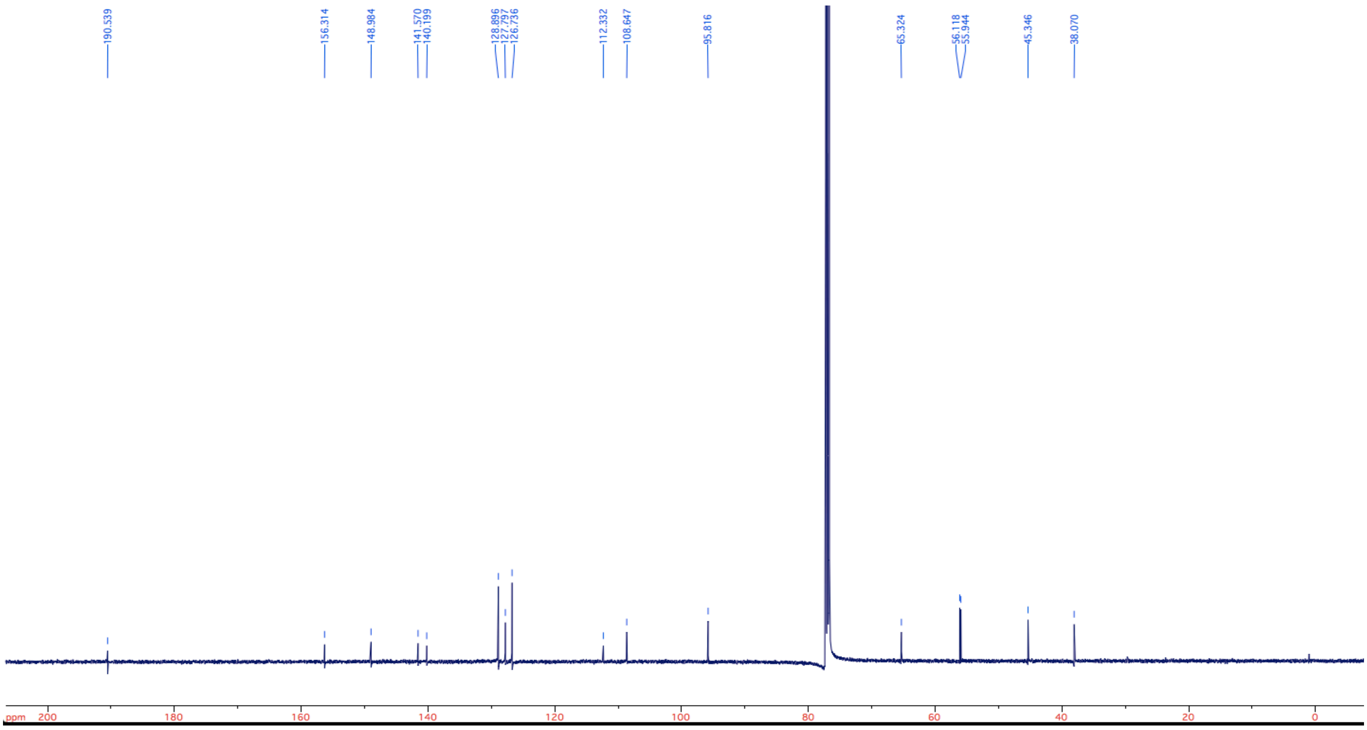

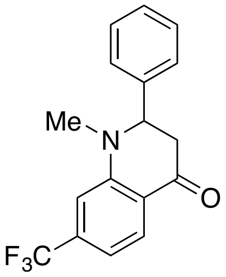

10q

^{1}H NMR (400 MHz, $CDCl_3$)

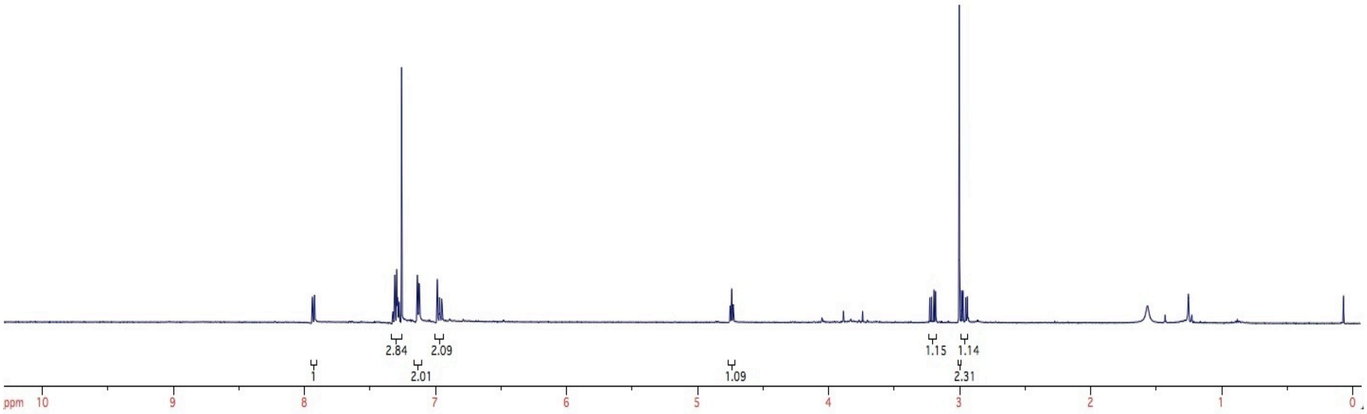

^{13}C NMR (100 MHz, $CDCl_3$)

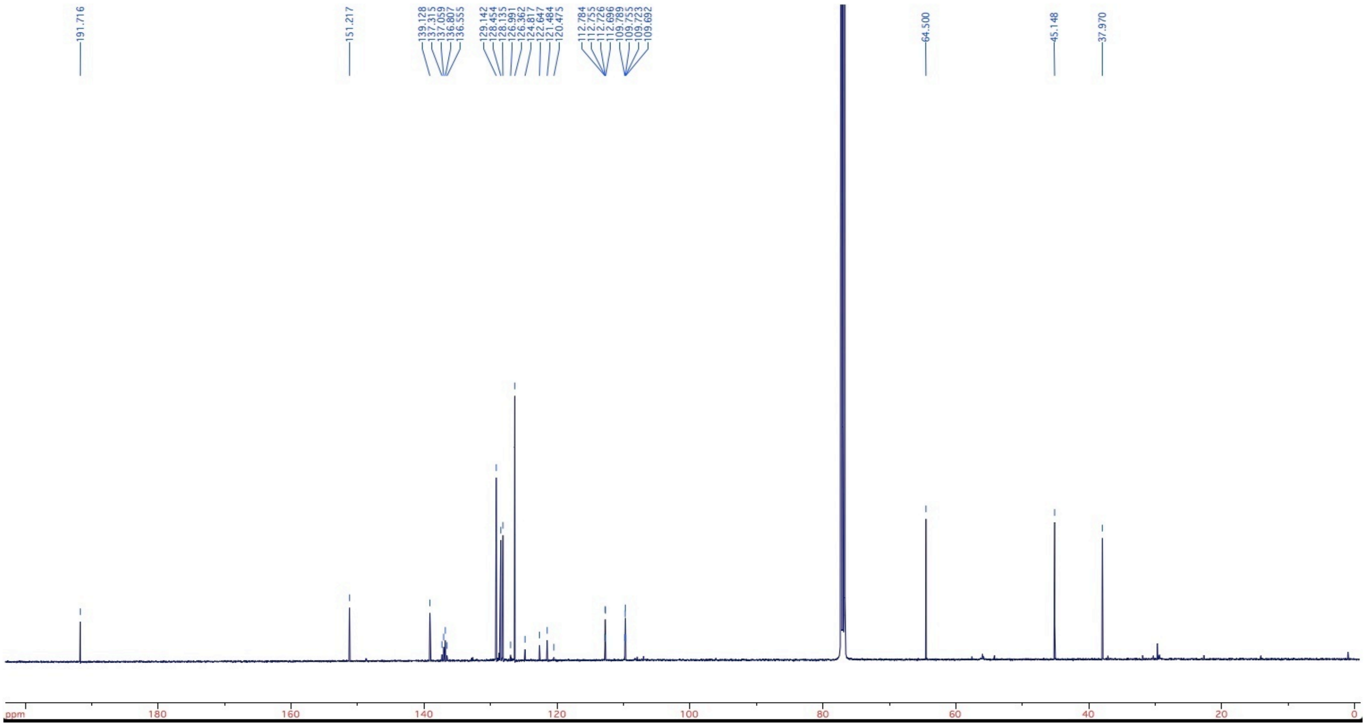

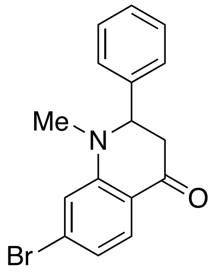

10r

^{1}H NMR (400 MHz, $CDCl_3$)

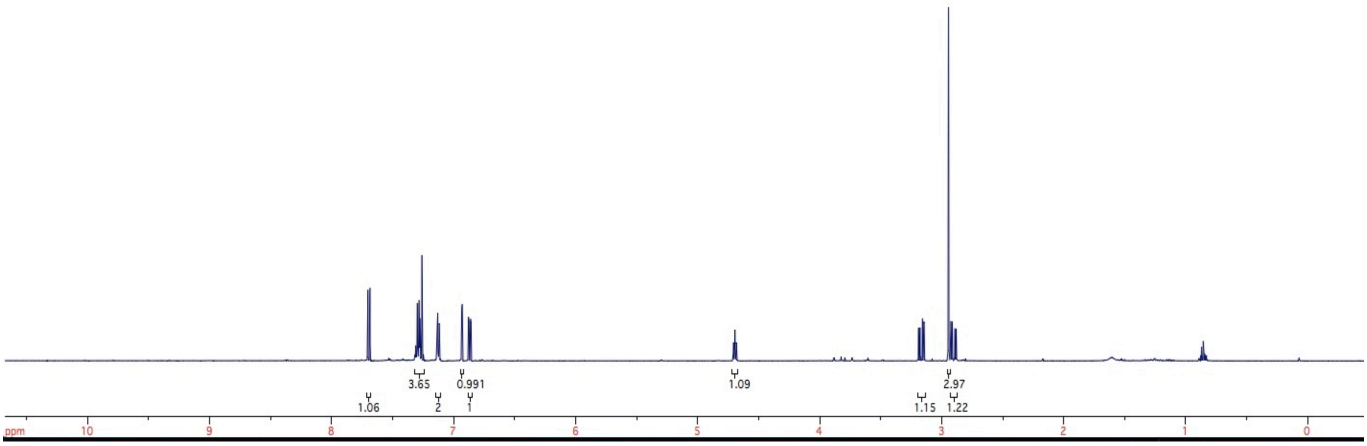

^{13}C NMR (100 MHz, $CDCl_3$)

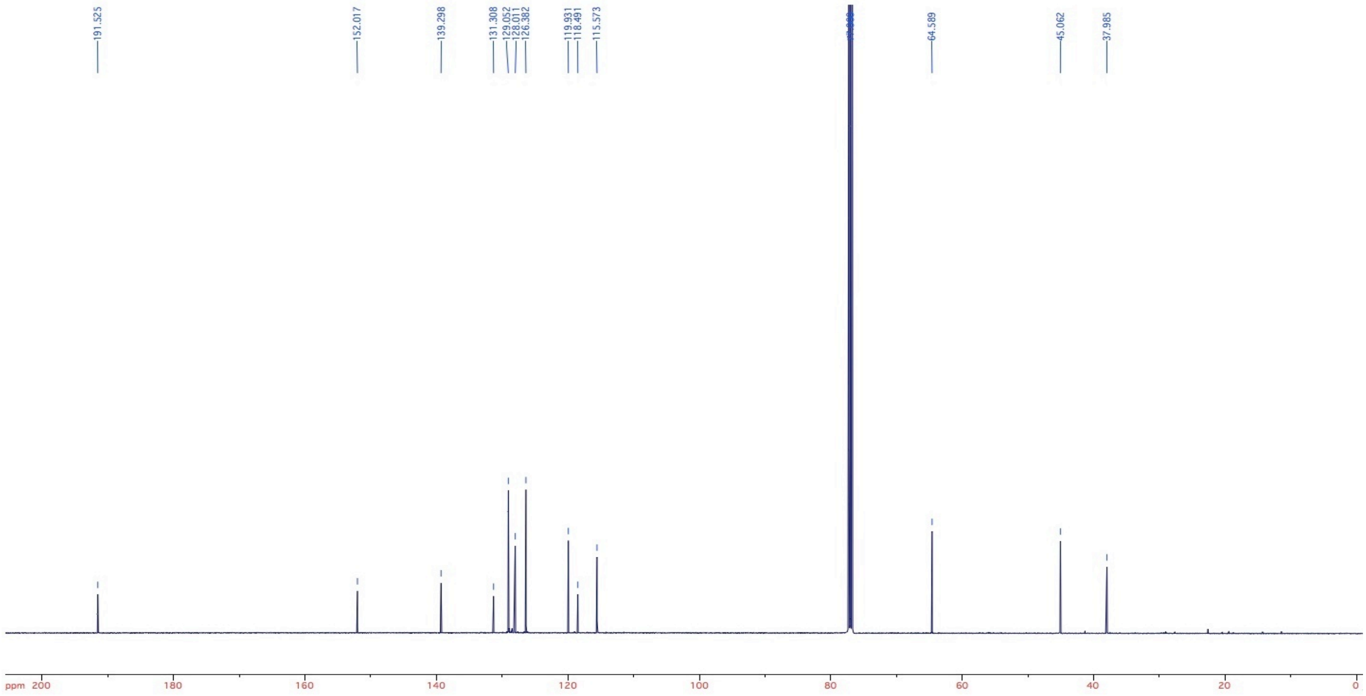

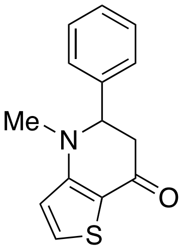

10s

^{1}H NMR (400 MHz, $CDCl_3$)

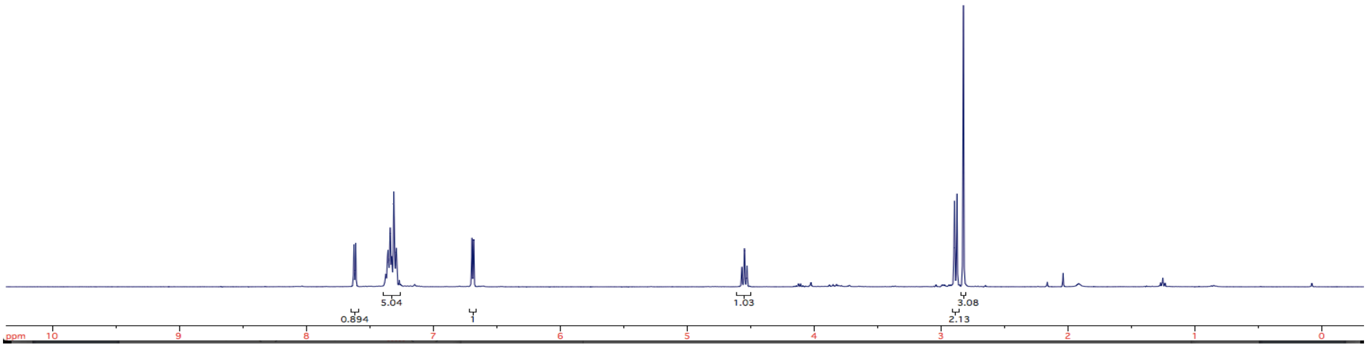

^{13}C NMR (100 MHz, $CDCl_3$)

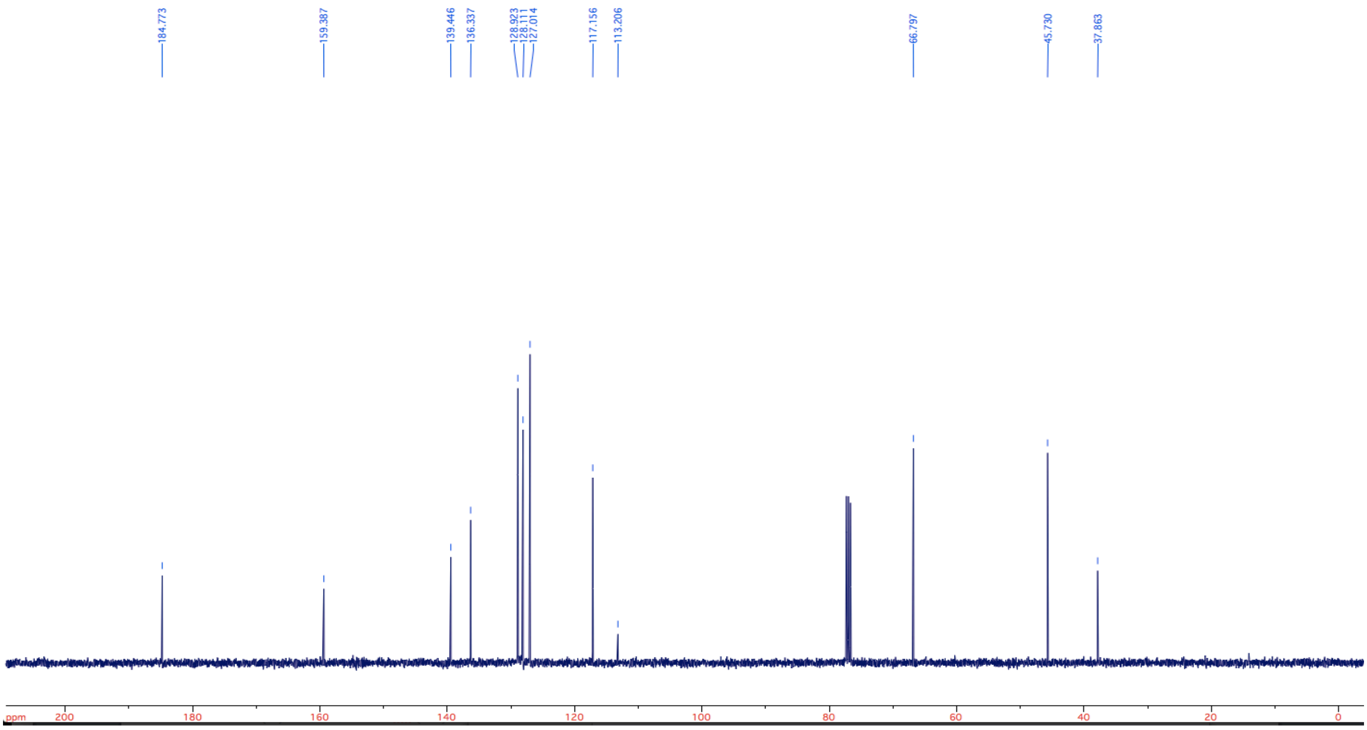

Supplement: Supplementary file 1 — miscellaneous_information [file anie0052-13280-SD1.pdf]
